# Road traffic noise affects annoyance during urban built and forest walks, but not repetitive negative thinking or connectedness with non-human nature: A randomized controlled trial

**DOI:** 10.1371/journal.pone.0342906

**Published:** 2026-03-18

**Authors:** Julia Schaupp, Jean-Marc Wunderli, Beat Schäffer, Karin Hediger, Nicole Bauer

**Affiliations:** 1 Swiss Federal Institute for Forest, Snow and Landscape Research (WSL), Birmensdorf, Switzerland; 2 Faculty of Psychology, Department of Clinical Psychology and Psychotherapy, University of Basel, Basel, Switzerland; 3 Empa, Swiss Federal Laboratories for Materials Science and Technology, Dübendorf, Switzerland; 4 Faculty of Behavioural Sciences and Psychology, Department of Child and Adolescent Psychology, University of Lucerne, Luzern, Switzerland; 5 Department of Epidemiology and Public Health, Swiss Tropical and Public Health Institute, Allschwil, Switzerland; 6 Faculty of Psychology, Open University, Heerlen, the Netherlands; Shenyang Jianzhu University, CHINA

## Abstract

Previous research has examined the potential of exposure to greenspaces to reduce maladaptive emotion regulation strategies, such as repetitive negative thinking, and to foster connectedness with non-human nature. However, research comparing green and urban built environments often primarily attributes the benefits of greenspace exposure to their positive features, without explicitly addressing road traffic noise as a potential stressor in urban settings. This study investigated how 30-minute walks in forests and urban built environments with varying levels of road traffic noise in Zürich, Switzerland, affect noise annoyance, connectedness with non-human nature and repetitive negative thinking. By accounting for traffic noise, the aim was to isolate the effects of environment (urban built. vs. forest) with minimal confounding. Data from 354 healthy adults were analyzed. Road traffic noise annoyance was generally lower (i) in forests than urban built settings, (ii) in lower road traffic noise than higher road traffic noise settings, and (iii) in areas with longer than in those with shorter relative quiet time. Notably, the robust association between road traffic noise and annoyance was particularly pronounced in forests. In contrast, neither walking in forests nor in environments with lower road traffic noise nor such with longer relative quiet time was associated with a greater reduction in repetitive negative thinking, a stronger increase in nature relatedness or love and care for nature. The results underscore the role of road traffic noise as a significant health-related stressor and support the need for targeted noise mitigation—particularly around urban forests— to preserve their restorative potential. Additionally, the results highlight the relevance of quiet intervals and temporal noise patterns in shaping perceived annoyance, extending beyond average noise measurements. This study has been registered in the ISRCTN registry (ISRCTN48943261). https://www.isrctn.com/ISRCTN48943261 Registered 23.11.2023.

## 1. Introduction

Urbanization is progressing rapidly around the globe. More than 50% of the world’s population live in cities, and this proportion is growing rapidly [1]. The shift from rural to urban living is accompanied by limited accessibility [2] and opportunities to spend time in greenspaces. In addition, urban areas are typically characterized by elevated traffic noise levels, which represents a significant environmental stressor [3,4]. Road traffic noise ranks among the most significant contributors to the environmental burden of disease in Western Europe, posing a substantial public health challenge [5]. The most common response in a population exposed to environmental noise is annoyance [6]. Previous research has indicated a considerable increase in noise annoyance with increasing road traffic noise [6–8].

Earlier studies have shown that exposure to greenspaces can promote psychological restoration and wellbeing [9–11]. Much of the research on the benefits of greenspaces draws from attention restoration theory (ART) [12]. According to ART, exposure to greenspaces facilitates restoration from a state of depleted direct attention by fostering a sense of being away (a sense of escape from daily demands), fascination (effortless attention drawn by natural elements), extent (a coherent, immersive setting), and compatibility (alignment with an individual’s needs and purposes) [13]. A related perspective is the salutogenic perspective outlined by Antonovsky [14] [cf. 15], proposing two central resources that support individuals in staying healthy despite the presence of stressors: generalized resistance resources (GRRs) and a sense of coherence. GRRs are factors that enable individuals to cope with stressors (i.e., internal ones such as personality traits or coping mechanisms, or external ones such as social support or financial security), while the sense of coherence is a person’s confidence that life is understandable, manageable and meaningful [14]. GRRs contribute to an individual’s sense of coherence.

There has been extensive research on the benefits of exposure to greenspaces on biomarkers of physiological stress [16,17], subjective restoration [9,18], affect [19], and attention [20,21]. By comparison, relatively few recent studies have compared the effects of exposure to greenspaces and urban built environments on maladaptive forms of emotion regulation, such as repetitive patterns of negative self-focused thinking, including rumination [22–24]. Besides the direct restorative benefits of exposure to greenspaces, other studies have compared the potential of exposure to greenspaces (vs. urban built environments) to change the relatedness of humans to non-human nature, leading to a more connected relationship. Results of such studies are mixed. Studies that have indicated differences in the effects of exposure to green and urban built environments have commonly attributed these to beneficial features of the greenspaces. However, it has been noted that differences between the benefits of greenspaces and urban built environments may occur due to additional stressors in the urban built environment rather than (only) the characteristics of the greenspaces, with one such (potentially important) stressor possibly being traffic noise [19]. Despite the general awareness of road traffic noise as an environmental stressor, previous research comparing the benefits of exposure to urban greenspaces and urban built environments on the relationship of humans with non-human nature and repetitive negative thinking (RNT) has rarely included explicit assessments of road traffic noise as a potential stressor.

The present study addresses this research gap. The aim of this work was to examine the effects of exposure to urban greenspaces vs. urban built environments during 30-minute walks on noise annoyance, on the relationship of humans with non-human nature, and on forms of RNT, while minimizing the impact of road traffic noise as an environmental stressor. In addition, the use of a factorial design allows for the separate assessment of the effects of both the walking environment (urban vs. forest) and road traffic noise. Further, many people go on walks in their everyday lives to reduce stress and experience calm [25], but few studies have explicitly investigated how people’s thoughts change when going for a walk. This study therefore additionally provides a qualitative analysis to gain a general deeper insight into whether and how walking influences thought patterns and problem-solving compared with daily life. The exact research questions and hypotheses guiding this investigation can be found in supplement S2. The studied health outcomes are further detailed in sections 2 and 3.4.

## 2. Background

### 2.1. Noise annoyance

Environmental noise is estimated to account for 24% of the total health loss due to environmental factors, underscoring its significant public health impact [26]. Exposure to traffic noise has been linked to adverse health effects, such as elevated blood pressure [27,28], hypertension [28,29], cardiovascular diseases [30] and depression [31]. Beyond the effects of objective noise exposure, it is important to understand how people subjectively perceive and react to it as disruptive or irritating [32]. Noise annoyance represents this subjective evaluation and serves as a key psychological factor; behind sleep disturbance, it is the second-most important cause of healthy life years lost in Europe [3]. This variable may mediate the connection between acoustic intensity (in decibels, dB) and related stress or health outcomes [33,34]. Research indicates that noise annoyance rises substantially with higher levels of road traffic noise [6–8]. In addition, longer quiet intervals between road traffic noise incidents have been linked to reduced noise annoyance [8,35]. Individuals who are annoyed by noise may react with a range of adverse responses, including feelings of anger, frustration, dissatisfaction, social withdrawal, helplessness, anxiety, depression, distraction, restlessness or fatigue [36–38].

### 2.2. The relationship of humans with non-human nature

Mayer et al. [39] point out that research on the wellbeing benefits of exposure to greenspaces should consider that people need to feel a sense of belonging to something larger than themselves, and that this need may be fulfilled through a sense of belonging or connectedness to the non-human natural world. Zylstra et al. [40] define such connectedness as a “stable state of consciousness comprising symbiotic cognitive, affective, and experiential traits that reflect, through consistent attitudes and behaviors, a sustained awareness of the interrelatedness between one’s self and the rest of nature.” Various scales have been developed to measure so-called nature connectedness. Some of these concepts tap into cognitive appreciation of being embedded in “nature” (e.g., *connectedness to nature,*  [41]), while others focus on the emotional aspects (*love and care for nature*, [42]). Despite their differences, all concepts seem to share the same core phenomenon: a relatively permanent connectedness with non-human nature on an individual level [43,44]. Since the nature connectedness perspective has faced criticism for defining “nature” in opposition to the human sphere, relying on a culturally specific nature–culture dichotomy that paradoxically reinforces the very separation it aims to bridge [45], we prefer the term “connectedness with non-human nature” [cf. 46] over “nature connectedness.” Accordingly, this term is used throughout the manuscript. However, in line with previous research [45,47], we acknowledge that the notion of “connectedness with non-human nature” still implies a relationship between conceptually distinct entities (humans and the broader biophysical world) and therefore does not fully escape this dichotomy.

Recent meta-analyses indicate that higher levels of connectedness with non-human nature are positively associated with wellbeing [48–50] and pro-environmental behavior [51,52]. Gál and Dömötör [53] showed that connectedness with non-human nature mediates the associations between nature contact and stress. Thus, if walking in greenspaces increases connectedness with non-human nature, it could in turn enhance human wellbeing and reduce stress, while promoting pro-environmental behavior.

Mayer et al. [39] found greater connectedness with non-human nature after walking in greenspaces for 15 minutes, compared with walking in an urban setting. Nisbet and Zelenski [54] found greater nature relatedness after 17-minute walks in urban nature, compared with indoor walks. Lumber et al. [55] compared 20-min group walks across three conditions: (1) a natural environment with activities focused on emotion-beauty (discussing thoughts and feelings about observed nature), meaning-beauty (writing about symbolic meanings in nature), and compassion-beauty (watching a video on creating wildlife-friendly gardens); (2) an urban environment with the same activities; and (3) a natural environment without any additional activities. Nature relatedness was assessed before and after the walks. In contrast to Mayer et al. [39], connectedness with non-human nature only increased in the condition in which participants walked in a natural environment with the additional activities, and not in the other two conditions. Thus, evidence on the effects of exposure to greenspaces on connectedness with non-human nature is mixed.

Previous research has concluded that different types of greenspaces may affect connectedness with non-human nature differently [55]. To date, several studies neglected to closely describe not only the setting they tended to generally call “nature,” but also possible environmental stressors potentially disparate between greenspaces and urban built reference settings. Such differences may partly explain variations in the benefits of greenspace exposure compared with urban built environments on individuals’ connectedness to the non-human world. While existing research on benefits of exposure to greenspaces has often involved using visual stimuli as a focus [56,57], sound is also an important pathway through which the environment is perceived by humans and can positively influence individuals’ connectedness to the non-human world [58,59]. Accordingly, links have been reported between both long- and short-term noise exposure and increased stress and reduced wellbeing [60–62]. However, the specific impact of road traffic noise on connectedness to the non-human world remains largely unexplored. To our knowledge, only one study has investigated this relationship, and participants with greater nature-relatedness were reported to be more likely to notice traffic sounds and to perceive them as unpleasant [63]. In line with the constrained restoration concept [64,65], which suggests that certain environmental factors can hinder restoration, road traffic noise may also constrain the beneficial effects of exposure to greenspaces on connectedness to the non-human world.

### 2.3. Repetitive negative thinking

Previous research has focused more on the promotion of positive mechanisms than on the attenuation of negative mechanisms through exposure to greenspaces. RNT is one such negative mechanisms that is possibly attenuated through exposure to greenspaces. RNT describes “negative, repetitive, and uncontrollable thoughts that are intrusive and difficult to disengage from” [66]. It is perceived as unproductive and requires mental capacity [67]. Two forms of RNT are worry and rumination. Heightened levels of RNT in the form of worry and/or rumination have been associated with various emotional problems [66,68,69]. A small number of studies have involved examining rumination in the context of exposure to natural environments. A cross-sectional study indicated a negative association between average weekly time spent in nature and self-reported rumination [70]. Two studies have examined the effect of a 50-minute [22] and a 90-minute [71] walk in a natural compared with a busy urban built environment, and both showed a greater decrease in rumination in the natural compared with the urban built environment. Another study [23] showed that a 30-minute walk in an urban park resulted in a stronger decrease in ruminative thinking than a walk along a city transect devoid of natural elements. In fact, participants in the urban built condition showed no changes in rumination from before to after the walk, while the walk in the park significantly reduced rumination [23]. However, the authors noted that the rumination-reducing effect of the walk in the park might be due to the sensorial experience in the park (e.g., sound reduction) and suggested future research that includes objective assessments of variables like traffic (noise) in both the greenspace and the comparison setting [23]. We are not aware of any studies on the effects of road traffic noise on RNT, neither in the form of worry, nor in the form of rumination. To help fill this knowledge gap, the present study was conducted to explore the effects of exposure to greenspaces compared with urban built environments on rumination, while minimizing the confounding influence of road traffic noise.

## 3. Methods

This paper builds on a large study assessing facilitators and barriers to psychological restoration during walks in different noise-exposed urban forests and urban built environments [72]. The overall study assesses different aspects, some of which have been published previously. A previous paper [11] concentrates on how environment (forest vs. urban built) and objectively measured road traffic noise influence outcomes of psychological restoration (salivary cortisol, feelings of restoration and stress recovery, perceived restorativeness of the environment, affect and attention) during brief walks. This paper builds on the same study design, but, rather than outcomes of psychological restoration, its fucus is on the effects of environment and road traffic noise on individuals’ subjective perception and reaction to noise (annoyance), as well as on the potential of exposure to greenspaces vs. urban built environments to change the connectedness of humans with non-human nature and maladaptive forms of emotion regulation (RNT).

### 3.1. Participants

A total of 354 participants (66.1% females) between 18 and 86 years of age (M = 41.24, SD = 18.57) were included in the analysis. Of the participants, 39.1% were employed part-time and/ or 33.5% were studying, while 18.6% were pensioners and 17.8% worked full-time. A power analysis was conducted for the previously published study with the same design. As to our knowledge, no previous study with a similar design has used any of the outcome measures considered in the present study, the same estimates and analysis as applied in Schaupp et al. [11] were utilized for this study (based on Restoration Outcome Scores in a study by Tyrväinen et al. [9]). The power analysis used a simulation-based approach to determine the sample size for a linear mixed-effects model with fixed effects for environment and time and random effects for participant groups who completed the walk together, and the number of simulations with a significant interaction effect between environment and time was calculated. Assuming a power of ≥ 0.8, an alpha level of 0.05 and rounding up to an equal number of groups in urban built environments and forests, the analysis showed that a minimum of 10 groups in urban built environments and 10 in forests were needed. In total, 97 groups were analyzed across the four experimental conditions: 19 in urban built areas with high traffic noise, 22 in urban built areas with low traffic noise, 20 in forests with high traffic noise, and 36 in forests with low traffic noise. The uneven group distribution reflects an intentional oversampling in the forest low traffic noise condition, which was necessary to explore an additional research objective embedded within the broader study. This disproportionate sampling did not appear to introduce any statistical bias or compromise the validity of the analyses.

**Fig 1:**
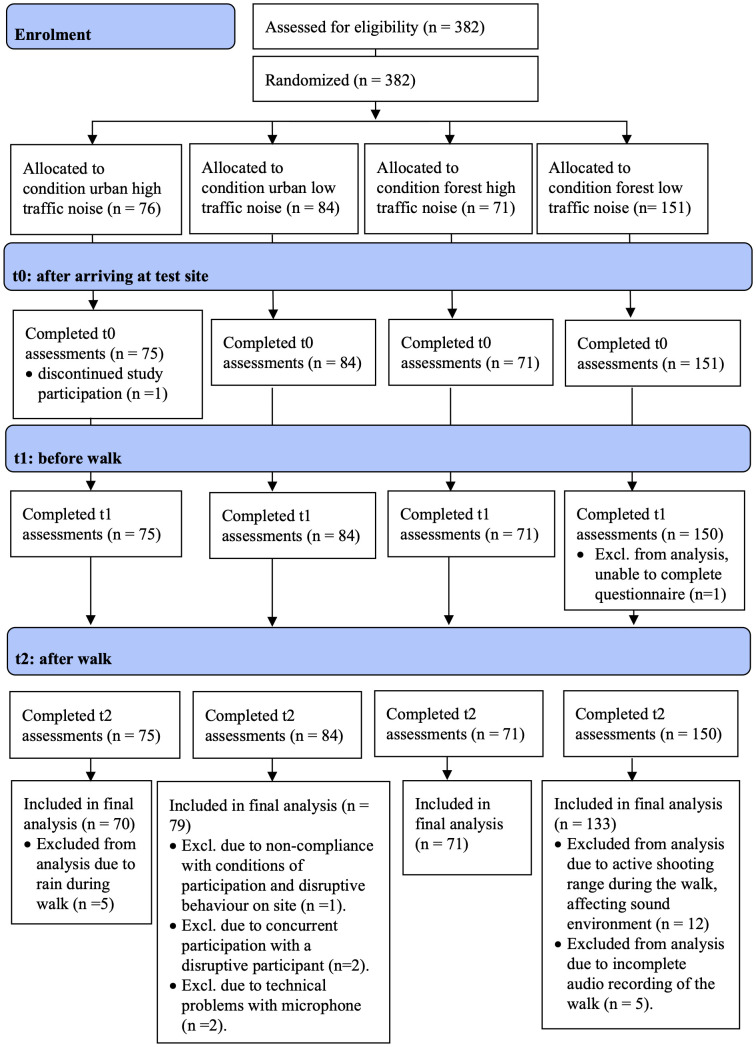
Participant flow diagram with number of included and excluded participants. Adapted and modified, from Schaupp et al. [11], licenced under CC BY 4.0.

Eligible participants were aged 18 years or older, were physically able to walk for 30 minutes at a moderate pace, had a BMI < 35, and had adequate knowledge of German (the questionnaire and test instructions were in German). Participants were excluded if they had diagnosed hearing problems. Participants were recruited using a random sample of 7000 inhabitants of Zürich, stratified into four age groups (18–35 years, 35–50 years, 50–65 years and > 65 years), drawn from the city of Zürich’s residents’ registration office. Additional participants were recruited via the universities in Zürich, local newspapers, public invitation flyers, and the researchers’ social networks. For a participant flow diagram, see Fig 1.

Participants registered for the study via a link to an online tool and were randomly allocated to one of the study conditions by the random generator within the online questionnaire tool SoSci Survey. This initial assignment followed a simple randomization protocol. After the 2022 and 2023 data collection phase, the distribution of participants across conditions was evaluated. To correct for observed imbalances, subsequent participants were assigned using an adaptive allocation strategy, in which more timeslots for underrepresented conditions were scheduled to achieve more balanced conditions. This ensured both initial randomness and overall balance in condition allocation across the full sample period (2022–2024).

Participants were informed that the study involved a walk in Zürich, but neither which conditions the experiment entailed nor which condition they had been assigned to. Each participant received a meeting point—either at the forest edge or in an urban built area—which may have given them a general idea of the walk’s setting prior to the walk on site. The investigators were not blinded; based on the meeting location, they were aware of the environment and thus the assigned condition for each group. Participation was compensated with 50 CHF.

### 3.2. Procedure

The study was approved by the Ethics Committee of the Swiss Federal Institute of Technology Zürich, Switzerland (Ref No. EK 2021-N-211 of 27.01.2022). It was pre-registered (trial registration number ISRCTN48943261) and a study protocol with a detailed description of the methods of the RCT was published [72]. A maximum of six participants were invited as a group for a guided walk. The participants were randomly allocated to one of four conditions: (1) an urban built environment with high road traffic noise, (2) an urban built environment with low road traffic noise, (3) a forest with high road traffic noise, or (4) a forest with low road traffic noise. Data were collected in three waves in the years 2022, 2023 and 2024. Data collection started on 05/2022 and ended 06/2024. All data were collected in the afternoon (between 2:00 p.m. and 5.30 p.m.) of weekdays (Monday to Friday) between May and October.

Upon arrival at the test location, participants were given a short overview of the study’s research topic, the study procedure*,* and the privacy guidelines, they provided written informed consent by signing an informed consent form, and responded to a series of questionnaires (see section 3.4.1; full questionnaire available from Schaupp et al. [73]). During the walk, participants were guided along a predetermined route, walking at an approximate speed of 4.7 km/h. Throughout the walk, the experimenter continuously measured ambient sound levels using a class 1 sound level meter (model XL2, NTI Audio, Schaan, Liechtenstein) equipped with a free-field measurement microphone, compliant with IEC 61672 environmental standards (see Schaupp et al., [11]). Participants were asked to remain silent during the walk and to keep a minimum distance of 6 meters from the experimenter to ensure that their footsteps did not interfere with the noise measurements. After the walk, participants completed another questionnaire (see section 3.4.1).

### 3.3. Test sites

A within subject design was applied in the study, with participants walking in one of the four environment types in the city of Zürich, Switzerland (urban built environment with low or high traffic noise, urban forest with low or high traffic noise). For each condition type, three distinct settings were chosen, leading to a total of 12 different test sites.

The test sites were preselected according to simulated road traffic noise levels [74] and vegetation cover (vegetation height model [VHM] [75] in meters and normalized difference vegetation index [NDVI] [76], ranging from −1 to 1). Satellite imagery was reviewed to evaluate the size of the sites, the presence of sidewalks, and the density and height of surrounding buildings. Sites containing bodies of water or steep terrain were excluded. To verify sufficient variation in noise levels across the selected sites, sound pressure levels were recorded during 30-minute walks conducted at different times in the afternoon. A visual impression of all test sites can be found in Table 1 of the study protocol [72]. After the test site selection process, a post-hoc classification of the amount of vegetation in the test sites was performed by calculating the NDVI and VHM along each walking route with 50-m-wide buffer zones on both sides of the lines. In addition, forest composition and acoustic properties were calculated across all test sites. A description of the assessment of the acoustic parameters is provided in sections 3.4.1 and 3.4.2. For a quantitative characterization of the vegetation characteristics and acoustical properties by condition, as well as forest composition across test sites, see Table 1 and supplement C in Schaupp et al. [11]. The test sites represent typical urban built and urban forest settings in Zürich and were selected for their practical relevance as common walking areas for the city’s residents. However, they may not fully represent forests and urban built environments more broadly.

**Table 1:** Demographics by condition.

	UBHT	UBLT	FHT	FLT	Total
	n = 70	n = 79	n = 71	n = 133	n = 353
Age in years, M (SD)	42.13 (18.71)	45.75 (19.11)	41.17 (18.15)	38.13 (18.00)	41.24 (18.57)
Gender n (%)					
Female	54 (77.1%)	55 (69.6%)	47 (66.2%)	78 (58.6%)	234 (66.3%)
Male	14 (20.0%)	24 (30.4%)	23 (32.4%)	54 (40.6%)	115 (32.6%)
Other	2 (2.9%)	0 (0%)	0 (0%)	1 (0.8%)	3 (0.9%)
Main occupation n (%)*					
Employed (full-time)	9 (12.9%)	15 (19.0%)	15 (21.1%)	20 (15.0%)	59 (16.7%)
Employed (part-time)	33 (47.1%)	34(43.1%)	24 (33.8%)	50 (37.6%)	141 (39.9%)
In pension	13 (18.6%)	16 (20.3%)	14 (19.7%)	23 (17.3%)	66 (18.7%)
Homemaker	1 (1.4%)	3(3.8%)	1 (1.4%)	6 (4.5%)	11 (3.1%)
Unemployed	2 (2.9%)	2(2.5%)	5 (7.0%)	3 (2.3%)	12 (3.4%)
In training/ studying	22 (41.4%)	25(31.6%)	18 (25.4%)	55 (41.4%)	120 (34.0%)
Highest education n (%)					
Primary/ secondary	1 (1.4%)	3 (3.8%)	1 (1.4%)	3 (2.3%)	8 (2.3%)
Vocational school	10 (14.3%)	15 (19.0%)	9 (12.7%)	14 (10.5%)	48 (13.6%)
High school	20 (28.6%)	6 (7.6%)	15 (21.1%)	43 (32.3%)	84 (23.8%)
Higher technical or vocational	10 (14.3%)	11 (13.9%)	8 (11.3%)	7 (5.3%)	36 (10.2%)
Applied univ./teacher training	9 (12.9%)	10 (12.7%)	9 (12.7%)	19 (14.3%)	47 (13.3%)
Technical college/ univ.	20(28.6%)	34 (43.0%)	27 (38.0%)	46 (34.6%)	127 (36.0%)
Greenspace visits n (%)					
daily	11 (15.7%)	9 (11.4%)	5 (7.04%)	14 (10.5%)	39 (11.0%)
several times a week	32 (45.7%)	38 (48.1%)	37 (52.1%)	60 (45.1%)	167 (47.3%)
once a week	17 (24.3%)	24 (30.4%)	14 (19.7%)	36 (27.1%)	91 (25.8%)
2–3 times a month	9 (12.9%)	6 (7.59%)	10 (14.1%)	19 (14.3%)	44 (12.5%)
Less than 2–3 times a month	1 (1.43%)	2 (2.53%)	4 (5.63%)	4 (3.0%)	11 (3.1%)

*Note.* UBHT = urban built high traffic noise, UBLT = urban built low traffic noise, FHT = forest high traffic noise, FLT = forest low traffic noise. *Multiple answers possible. For all demographics n = 1 missing in condition FLT. Adapted and modified from Schaupp et al. [11] licenced under CC BY 4.0.

### 3.4. Measures

The questionnaires were provided in German, Cronbach’s alpha (α) and McDonald’s omegas (ω) values stem from the current sample at time point T1 (before the walk). Different response scale ranges were used across the assessed measures (i.e., 0–4 for RNT, 1–7 for love and care for nature, 1–5 for nature relatedness, and 0–10 for noise annoyance), reflecting the original validated instruments. Maintaining the original response ranges preserves their established psychometric properties and allows for direct comparability with previous research using the same measures.

#### 3.4.1. Questionnaires.

**Repetitive negative thinking:** RNT (e.g., rumination and worry) was assessed with an adapted German version of the Perseverative Thinking Questionnaire (PTQ) [67]. The PTQ consists of 15 items (e.g., “the same thoughts keep going through my mind again and again,” “thoughts come to my mind without me wanting them to,” etc.), which participants rate on a Likert scale ranging from 0 (*never*) to 4 (*almost always*). Before the walk, participants were asked to rate how they *typically* think about negative experiences or problems in everyday life. After the walk, individuals stated how they thought about negative experiences or problems *during the walk* they just took*.* A high internal consistency for a nonclinical sample (Cronbach’s α = .94) and convergent validity of the PTQ have been shown previously [67]. For the current sample, a Cronbach’s α = 0.92 and McDonald’s ω = 0.92 were obtained at T1.

**Nature relatedness:** Nature relatedness was measured with the German version [77] of the short form of the Nature Relatedness Scale (NR-6) [78]. Nature relatedness is assumed to reflect a person’s interest in, fascination with and desire for contact with nature [79]. Items (e.g., “I always think about how my actions affect the environment” and “My relationship to nature is an important part of who I am,” etc.) are rated on a five-point Likert scale ranging from 1 (*strongly disagree*) to 5 (*strongly agree*). For the current sample, a Cronbach’s α = 0.77 and McDonald’s ω = 0.78 for T1 were obtained at T1.

**Love and care for nature:** Love and care for nature was measured with the Love and Care for Nature Scale (Perkins, 2010). This scale assesses love and deep caring for nature as an expression of affective components of people’s relationship with nature. Perkins [42] conceptualizes love and care for nature as a profound affection and sense of responsibility to protect nature, grounded in an acknowledgment of its inherent worth. The five-item version (e.g., “I feel a deep love for nature”) was employed with a seven-point Likert scale ranging from 1 (*strongly disagree*) to 7 (*strongly agree*). Good internal consistency has been shown for the scale [42]. For the current sample, Cronbach’s α = 0.83 and McDonald’s ω = 0.84 were obtained at T1.

**Noise annoyance:** Noise annoyance was assessed with the 11-point scale developed by the International Commission on Biological Effects of Noise (ICBEN) [37], as recommended by the International Organization for Standardization (ISO/TS 15666) [80]. Participants rated their annoyance regarding road traffic noise by answering the following question (adapted from ISO/TS15666): “*During your walk, how much did the following sources of noise bother or annoy you? (Road traffic (cars, trucks)).”* Responses were given on the numerical 11-point scale from 0 (*not at all*) to 10 (*extremely*). This standardized noise annoyance scale is widely applied in noise research, providing internationally comparable measures of annoyance reactions across studies [37].

**Additional subjective acoustical assessments:** The acoustic environment was evaluated according to the ISO/TS 12913−2 standard (annex C.3.1.2 in [81]). Participants rated their perception of traffic noise, human sounds, natural sounds and other sound types on a five-point Likert scale ranging from 1 (*not at all*) to 5 (*dominates completely*). Additionally, they rated the overall sound environment quality during the walk on a 10-point Likert scale, ranging from 1 (*very pleasant)* to 10 (*very unpleasant*) (adapted from annex C.3.1.3).

**Open-ended questions:** To get a better insight into how walking affects people’s thoughts, the following two open-ended questions were integrated into the questionnaire: *“How do your thoughts change when you go for a walk?”* and *“How does going for a walk help you to think through personal problems?”*

#### 3.4.2. Assessment of road traffic noise.

Audio recordings captured during the walks were analyzed to determine the predominant sound source for each one-second time interval. Time segments were classified based on whether road traffic noise was the dominant source or not. To quantify objective noise exposure, the corrected sound exposure level (*L*_AE_) was computed for all time periods in which traffic noise was dominant. Periods when road traffic noise was not dominant were included using an equivalent continuous A-weighted sound pressure level (*L*_Aeq_) of 30 dBA.

The cumulative exposure to the dominant traffic noise was calculated as


$$L_{AE}=L_{Aeq}+{log}_{\text{1}\text{0}}(T),$$

where the logarithmic scale of time *T* to derive the *L*_AE_ from an *L*_Aeq_ (during time interval T) is given by the logarithmic scale of sound pressure levels (in dB) used in acoustics, which is meaningful also insofar as the sensation of the human ear follows basically a logarithmic law. The complete formulas for calculating the *L*_AE_—including periods with and without dominant traffic noise—are provided in Supplement S3. The relative quiet time (RQT), which represents the proportion of the time during which traffic noise was not dominant (T_TrafficNoiseNotDom_) relative to the total duration of the walk (T_Walk_), was calculated as


$$\text{RQT }(\%)=\text{100}*\frac{T_{TrafficNoiseNotDom}}{T_{Walk}}$$

### 3.5. Data analyses

#### 3.5.1. Quantitative analyses.

Outcomes were analyzed using linear mixed-effects modeling in R version 4.4.2 [82] with the lme4 package [83]. Evaluations of the pattern of missing values revealed no variables with ≥ 5% missing values. Missing data were handled using maximum likelihood estimation within the linear mixed models, which allowed the inclusion of all available data without the need for data imputation. All models included fixed effects for the walking environment, road traffic noise and the environment × traffic noise interaction. To account for the hierarchical structure of the data, the group participants walked in was included as a random intercept. The models for RNT, nature relatedness, and love and care for nature were performed using the delta scores (before-after) as outcomes, while annoyance was only assessed after the walk. Positive RNT, nature relatedness and love and care for nature delta scores indicate a decrease in the outcome, while negative delta scores reflect an increase. Three models were computed for each outcome variable. The first model incorporated walking environment (forest vs. urban built) and road traffic noise level (high vs. low) as categorical predictors. The second model treated noise as a continuous predictor, using the sound exposure level (*L*_AE_) to capture variations in noise exposure. In the third model, RQT replaced *L*_AE_ and was used as an alternative parameter to assess traffic noise exposure during the walk. The model results with road traffic noise as a continuous variable (*L*_AE_) as well as the model results with RQT can be found in supplement S4. All models were initially calculated without covariates and then recalculated with covariates, including age, gender, frequency of greenspace visits in the past year and highest level of education (i.e., model adjustment). Occupational status was not included as a covariate because this variable allowed multiple responses (e.g., respondents could indicate both studying and working), which precluded a clear categorical coding suitable for inclusion in the models. Treating such data as a single categorical or dummy-coded predictor would have required collapsing categories or imposing arbitrary coding decisions, potentially biasing parameter estimates and complicating model interpretation. Participants identifying as ‘other’ gender (n = 3) were excluded from models that included noise as a dummy-coded predictor due to their small sample size and lack of presence in certain conditions. The model results without covariates are reported below, while the results for models with covariates can be found in supplement S5.

In models with continuous noise predictors, post-hoc marginal effects were estimated to compare forest and urban built environments at the mean noise level, and to compare high vs. low noise level or RQT conditions across the environmental settings. Additionally, the relationships between *L*_AE_ or RQT and the outcomes were evaluated separately within each environment type (urban vs. forest) by computing marginal effects while setting environment to a specific level (0 = urban, 1 = forest).

Tested effects were considered significant if the probability (*p*) of the observed results, or more extreme results, under the null hypothesis was ≤ 0.05. However, the interpretation of the confidence intervals instead of the *p*-values is emphasized, following current recommendations that prioritize effect sizes and estimation over reliance on fixed significance thresholds [84,85].

#### 3.5.2. Qualitative analysis.

To gain a deeper insight into how participants’ thoughts change when going for a walk and how walking helps them to think through personal problems, responses to the above-mentioned open-ended questions were analyzed using qualitative content analysis [86]. The qualitative analysis followed an inductive approach, relating to the step model of inductive category development [87]. After the first author coded all answers for the first time, the developed category system was revised and re-rechecked during a second round of coding of all answers by the first author. The original language of the material was German.

## 4. Results

### 4.1. Quantitative results

The demographic characteristics of the participant by condition are displayed in Table 1. A one-way analysis of variance (ANOVA) showed a significant effect of condition on age (*F*(3, 348) = 2.90, *p* = .035). A Fisher’s exact test was conducted to examine the association between condition and gender (female, male, and other). The test revealed a significant association, *p* = .028.

Table 2 displays the means and standard deviations for the outcome variables by condition across measurement time points for the models with noise level as a categorical predictor. Descriptively, noise annoyance due to road traffic noise decreased in the order: urban built with high traffic noise condition > forest with high traffic noise condition > urban built with low traffic noise condition > forest with low traffic noise condition. Descriptively, RNT and love and care for nature tended to decrease from t1 to t2 in all conditions, while nature relatedness did not show consistent trends for the change from before to after the walk.

**Table 2:** Descriptive statistics across measurement points by condition.

Measure	Before walk				After walk			
	UBHT	UBLT	FHT	FLT	UBHT	UBLT	FHT	FLT
	M(SD)/ *n*	M(SD)/ *n*	M(SD)/ *n*	M(SD)/ *n*	M(SD)/ *n*	M(SD)/ *n*	M(SD)/ *n*	M(SD)/ *n*
Noise annoyance					9.00 (2.05)/ 70	6.28 (3.18)/ 78	8.87 (2.30)/ 69	2.11 (1.92)/ 132
Repetitive negative thinking	1.93 (0.72)/ 70	1.89 (0.63)/ 79	1.77 (0.67)/ 71	1.83 (0.76)/ 133	1.23 (0.86)/ 70	1.18 (0.83)/ 78	1.14 (0.86)/ 70	1.23 (0.75)/ 132
Nature relatedness	3.86 (0.72)/ 70	3.79 (0.64)/ 79	3.71 (0.76)/ 71	3.62 (0.79)/ 133	3.86 (0.78)/ 70	3.75 (0.78)/ 78	3.74 (0.77)/ 70	3.66 (0.83)/ 132
Love and care for nature	6.09 (1.09)/ 70	6.11 (0.89)/ 79	6.12 (0.84)/ 71	5.86 (0.96)/ 133	5.98 (1.22)/ 70	5.99 (0.99)/ 78	6.04 (1.03)/ 70	5.81 (1.03) 132

*Note. n* = number of participants with available data for the outcome and time point, *M* = mean, *SD* = standard deviation. UBHT = urban built environment with high traffic noise, UBLT = urban built environment with low traffic noise, FHT = forest with high traffic noise, FLT = forest with low traffic noise. Noise annoyance scores ranged from 1 to 11. Repetitive negative thinking scores ranged from 0 to 4. Nature relatedness scores ranged from 1 to 5. Love and Care for nature scores ranged from 1 to 7.

**Fig 2:**
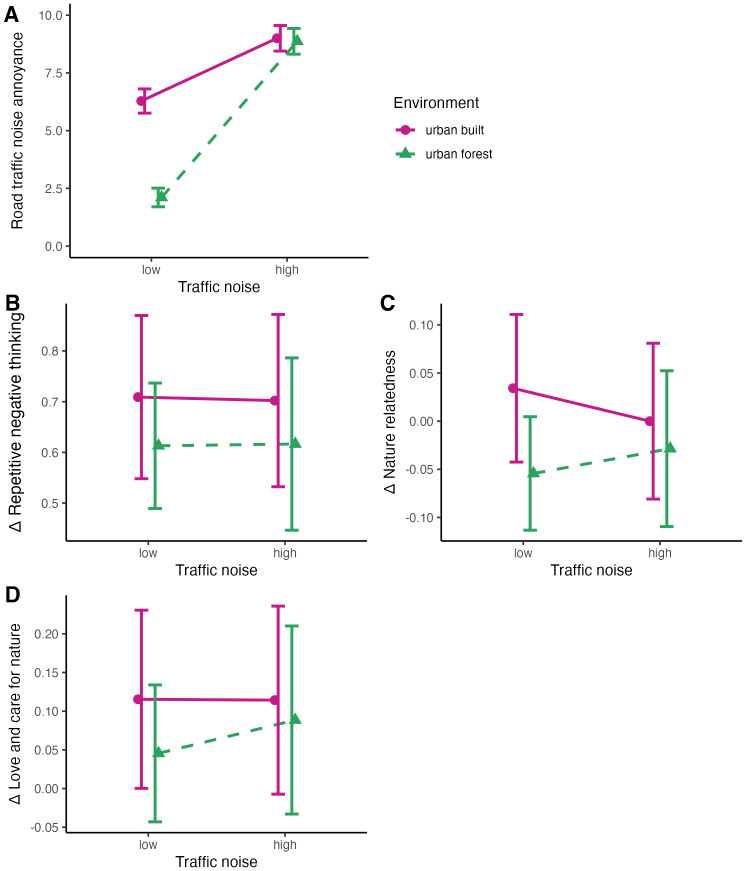
Estimates for noise annoyance (range 0-10), repetitive negative thinking (range 0-4), nature relatedness (range 1-5) and love and care for nature (range 1-7). Panels B—D depict differences (delta before minus after). Positive delta repetitive negative thinking scores reflect a decrease in repetitive negative thinking; positive nature relatedness scores indicate a decrease in nature relatedness and positive love and care for nature scores depict a decrease in love and care for nature.

Table 3 and Fig 2A show the results of the linear mixed-effects models with road traffic noise included as a categorical predictor (high vs. low exposure) for noise annoyance. Table 3 demonstrates strong evidence for an association between environment averaged across noise conditions and noise annoyance, as well as for a link between road traffic noise averaged across environment conditions and noise annoyance. Road traffic noise annoyance was lower in forest settings than in urban built settings, and lower in settings with low compared to such with high road traffic noise. The confidence intervals exclude zero and are relatively narrow, supporting the robustness and precision of the associations. Additionally, strong evidence was found for an interaction between environment and road traffic noise on noise annoyance. The increasing effect of road traffic noise on noise annoyance was considerably greater in forests than in urban built environments.

**Table 3:** Associations with road traffic noise annoyance.

	Estimate^2^	SE^3^	Lower CI^4^	Upper CI^4^	p
Effect of environment averaged across road traffic noise conditions^1^	−2.177	0.265	−2.698	−1.656	<.001
Effect of road traffic noise averaged across environment conditions^1^	4.685	0.265	4.164	5.207	<.001
Effect of road traffic noise in urban built conditions	2.718	0.388	1.955	3.481	<.001
Effect of road traffic noise in forest conditions	6.741	0.357	6.039	7.442	<.001
Environment: traffic noise interaction	4.046	0.522	3.018	5.073	<.001

^1^Calculated using Custom Contrasts, reference category for environment = urban, reference category for road traffic noise = low traffic noise. ^2^ Unstandardized estimate. ^3^Standard error, ^4^ CI = 95% confidence interval. *N = 341*.

Table 4 and Fig 2B present the results of the linear mixed-effects models using road traffic noise as a categorical predictor (high vs. low exposure) for change in RNT. Table 4 shows considerable statistical uncertainty for an association between environment and road traffic noise with change in RNT and for an interaction between environment and road traffic noise. While the point estimates suggest that RNT decreased in both conditions, the decrease was less pronounced in forests than in urban built environments. RNT was hardly affected by road traffic noise; Table 4 shows that the confidence intervals are very large and include zero, indicating that the association is negligible or even absent.

**Table 4:** Associations with change in repetitive negative thinking.

	Estimate^2^	SE^3^	Lower CI^4^	Upper CI^4^	p
Effect of environment averaged across road traffic noise conditions^1^	−0.096	0.082	−0.257	0.065	0.241
Effect of road traffic noise averaged across environment conditions^1^	0.002	0.082	−0.159	0.163	0.984
Effect of road traffic noise in urban built conditions	−0.014	0.121	−0.252	0.225	0.910
Effect of road traffic noise in forest conditions	0.017	0.110	−0.200	0.234	0.878
Environment: traffic noise interaction	0.031	0.164	−0.292	0.353	0.852

^1^Calculated using Custom Contrasts, reference category for environment = urban, reference category for road traffic noise = low traffic noise. ^2^ Unstandardized estimates. ^3^Standard error, ^4^ CI = 95% confidence interval. *N = 341*.

Table 5 and Fig 2C show the results of the linear mixed-effects models with road traffic noise included as a categorical predictor (high vs. low exposure) for change in nature relatedness. Table 5 indicates substantial statistical uncertainty for an association of environment and noise with change in nature relatedness. Point estimates suggest that nature relatedness increased in forests, compared with a marginal decrease in urban built environments, and tended to decrease more in environments with high traffic noise than in those with low traffic noise. Nonetheless, Table 5 shows that the confidence intervals include zero, indicating that the association may be negligible or absent. Also, no (strong) evidence for an interaction between environment and road traffic noise on nature relatedness was found.

**Table 5:** Associations with change in nature relatedness.

	Estimate^2^	SE^3^	Lower CI^4^	Upper CI^4^	p
Effect of environment averaged across road traffic noise conditions^1^	−0.057	0.039	−0.133	0.019	0.141
Effect of road traffic noise averaged across environment conditions^1^	0.005	0.039	−0.071	0.080	0.905
Effect of road traffic noise in urban built conditions	−0.020	0.057	−0.132	0.093	0.732
Effect of road traffic noise in forest conditions	0.029	0.052	−0.073	0.131	0.580
Environment: traffic noise interaction	0.048	0.077	−0.103	0.200	0.532

^1^Calculated using Custom Contrasts, reference category for environment = urban, reference category for road traffic noise = low traffic noise. ^2^ Unstandardized estimates. ^3^Standard error, ^4^ CI = 95% confidence interval. *N = 341.*

Finally, Table 6 and Fig 2D show the results of the linear mixed-effects models with road traffic noise included as a categorical predictor (high vs. low exposure) for change in the Love and Care for Nature Scale. As for RNT and nature relatedness, Table 6 reveals substantial statistical uncertainty for an association of environment and road traffic noise with changes in the Love and Care for Nature Scale. While point estimates suggest that love and care for nature decreased slightly in all conditions, the decrease was less pronounced in forests, compared with urban built environments and love and care for nature decreased slightly more in environments with high traffic noise than in those with low traffic noise. Table 6 shows that the confidence intervals include zero, indicating that the true association may be negligible or absent. Also, no (clear) evidence for an interaction between environment and road traffic noise on love and care for nature was found.

**Table 6:** Associations with change in Love and Care for Nature Scale.

	Estimate^2^	SE^3^	Lower CI^4^	Upper CI^4^	p
Effect of environment averaged across road traffic noise conditions^1^	−0.041	0.057	−0.153	0.072	0.479
Effect of road traffic noise averaged across environment conditions^1^	0.046	0.057	−0.067	0.158	0.424
Effect of road traffic noise in urban built conditions	0.030	0.085	−0.136	0.196	0.724
Effect of road traffic noise in forest conditions	0.062	0.077	−0.090	0.213	0.424
Environment: traffic noise interaction	0.032	0.114	−0.193	0.256	0.782

^1^Calculated using Custom Contrasts, reference category for environment = urban, reference category for road traffic noise = low traffic noise. ^2^ Unstandardized estimates. ^3^Standard error, ^4^ CI = 95% confidence interval. *N = 341.*

The results of the linear mixed-effects models with road traffic noise as a continuous variable (*L*_AE_) (S1–S4 Tables and S1 Fig in supplement S4), as well as the results of the mixed models with RQT (S5–S8 Tables and S2 Fig in supplement S4) were largely consistent with those reported for the models with dummy-coded noise regarding the direction of the effects for all outcomes. One inconsistency was that, compared with the model with dummy-coded noise and the model with RQT, the model with *L*_AE_ indicated less evidence for an association between environment and noise annoyance. Another slight inconsistency was the differing signs of the interaction between environment and road traffic noise on love and care for nature; however, the interaction term was non-significant in both cases. Further, the associations of *L*_AE_ and RQT with nature relatedness, love and care for nature and RNT were close to zero. Table 7 gives an overview of results of three models with the different noise predictors. The results of all models including covariates can be found in supplement S5. They are consistent with those without covariates regarding the direction of the effects for all outcomes. Overall, estimates become slightly stronger, indicating that covariates account for part of the effect but do not make a large difference.

**Table 7:** Overview of the results of three models with different noise predictors.

	Noise (low vs. high)			*L*_AE_^3^			RQT^4^		
	Environment (urban vs. forest)^1^	Road traffic noise (low vs. high)^2^	Environment: traffic noise interaction	Environment (urban vs. forest)	*L*_AE_	Environment: traffic noise interaction	Environment (urban vs. forest)	RQT	Environment: traffic noise interaction
Noise annoyance	-*	+*	+*	–	+*	+*	-*	-*	–
Δ repetitive negative thinking	–	+	+	–	0	–	–	0	0
Δ nature relatedness	–	+	+	–	0	+	–	–	+
Δ love and care for nature	–	+	+	–	+	–	+	–	+

^1^Urban built as reference category. ^2^Low traffic noise as reference category. ^3^Sound exposure level. ^4^Relative quiet time. *p < 0.05. Effects are rounded to three decimal places. 0 = zero effect (0.000); - = negative effect (< 0.000); *+* = positive effect (> 0.000).

### 4.2. Results from open-ended questions

When participants were asked to express their thoughts change when going for a walk, various topics were mentioned. Eleven different main categories with various subcategories were identified: (1) changes in the flow and intensity of thoughts; (2) change in the valence of thoughts (i.e., positive vs. negative); (3) change in the flow of thoughts; (4) change of perspective and relativization of thoughts; (5) space for thoughts; (6) creativity and solution-orientation; (7) gaining mental and emotional distance; (8) inner peace and relaxation; (9) thoughts become more structured and clearer; (10) change in thought content/focus; (11) unspecified categories. The complete final coding scheme can be found in supplement S6. The most frequently mentioned changes in participants' thoughts when going for a walk are indicated in Table 8.

**Table 8:** Most frequently mentioned changes in thoughts when going for a walk.

Mentioned topic	Frequency mentioned
1.Thoughts become calmer or more relaxed	63
2.Thoughts become clearer, more organized, or more sorted	38
3.Thoughts become more positive or focused on nice things	37
4.No change in thoughts	35
5.Thoughts focus on the surroundings	33
6.Thoughts are fleeting, flow naturally, come and go at will or can spread naturally	30
7.Thoughts become relativized, less important or less serious	22
8.Thoughts become more diverse, free, relaxed, creative or open	21
9.Thoughts become less frequent, fade into the background, or are less intrusive	20
10.Solutions and answers emerge; thoughts become more solution-oriented and constructive	19
11.Influence of new or different environments	18

When asked to express how going for a walk helps them think through personal problems, participants stated a variety of topics that are described below. Nine different main categories were identified: (1) clear the mind and gain some distance; (2) promotion of clarity, order and reflection; (3) gaining new perspectives and creative thinking; (4) providing emotional relief and stress reduction; (5) by experiencing nature and the surrounding area; (6) self-awareness and inner balance; (7) putting problems into perspective and finding solutions, (8) dependency on context and other influencing factors; and (9) no or limited effect of walking on thinking through personal problems. The complete final coding scheme can be found in supplement S7. The most frequently mentioned topics on how going for a walk helps one to think through personal problems are indicated in Table 9.

**Table 9:** Most frequently mentioned topics on how walking helps think through personal problems.

Mentioned topic	Frequency mentioned
1.Helps me (not further specified whereby)	44
2.Provides me with time and space to think	31
3.I become calmer/ can think more calmly/ have my peace	31
4.Creates distance from my everyday life, problems, or situations	28
5.Helps me gain a different or more distanced perspective/ another point of view	27
6.I find solutions to problems or answers	26
7.Reduces distractions (e.g., from mobile phone)	23
8.Physical exercise	22
9.I can gain clarity and clear my mind	21
10.Other (e.g., focusing on sensations like warmth or cold)	18
11.Encourages more diverse, flexible, creative, or complex thoughts and ideas	17
12.Helps me organize my thoughts, or my thoughts become more structured	17
13.Promotes relaxation	17
14.Improves concentration and focus	15

## 5. Discussion

### 5.1. Quantitative results

#### 5.1.1. Noise annoyance.

Consistent with our hypotheses, walking in environments with lower road traffic noise and more relative quiet time (free from road traffic noise) was associated with reduced noise annoyance. These findings align with previous findings on exposure–response relationships for road traffic noise and annoyance [8,88,89]. The result of greater noise annoyance in environments with higher road traffic noise and less relative quiet time is important, since noise annoyance potentially mediates the association between noise exposure and health outcomes [33,34]. This is in line with the constrained restoration concept and the assumption that certain environmental factors can hinder restoration. Road traffic noise may thus constrain beneficial effects of exposure to greenspaces, possibly via a mediation pathway by inducing noise annoyance. If road traffic noise constrains restorative experiences in urban environments over an extended period, this may undermine opportunities for restoration [cf. 11,64,65] and thus result in chronic stress. Road traffic noise may thus act as both a constraint to restoration and a contributor to chronic stress. Chronic stress in turn negatively impacts health outcomes [90–92].

The association between road traffic noise on noise annoyance was particularly large in forest settings. This aligns with research on the congruence between environmental visuals and sound [93]. Individuals expect correspondence between auditory and visual features, and incongruence is generally perceived negatively [94], implying that noise may be regarded more adversely in greenspaces than in urban built areas. Our findings thus underscore the importance of implementing road traffic noise mitigation strategies, particularly around urban forests and urban greenspaces. Despite the important role of road traffic noise in recreational areas, however, current noise abatement laws still do not extend legal protection to recreational areas in various countries. Previous Swiss research has suggested that daytime transportation noise should not exceed a threshold of 45 dB LAeq for urban green areas compatible with relaxation [95], which also lies in the range defined by the European Environment Agency [96]). In Swedish guidelines, a threshold of L_day_ < 50 dB(A) for traffic noise has been suggested for suburban greenspaces and urban parks [97]. Conversely to the relevance of noise mitigation and in line with previous research [2,96], our findings underscore the importance of incorporating and protecting quiet areas, undisturbed by unwanted noise, into urban planning to mitigate noise annoyance and to promote more liveable cities.

Similar to road traffic noise exposure, the finding that environments with more relative quiet time are associated with lower noise annoyance supports earlier studies indicating that noise intermittency can influence annoyance reactions [8,35]. This has been attributed to the role of noise-free intervals between noise events in moderating annoyance [8]. Consistent with previous research [11], this finding underscores the importance of relative quiet time and noise-free intervals for explaining noise annoyance, extending beyond average noise measurements.

The model with dummy-coded noise and RQT showed an association between environment and noise annoyance, with forests being associated with decreased noise annoyance, compared with urban built environments. No such association was shown in the model including traffic noise as *L*_AE._ The finding of decreased noise annoyance in forests, demonstrated through models using both dummy-coded noise predictors and RQT, aligns with prior findings linking residential green with noise annoyance [89,98,99]. Kawai et al. [2] reported that increasing greenness, reflected by a rise in NDVI from the 5^th^ to the 95^th^ percentile, was linked to a decrease in annoyance from road traffic noise—comparable in effect to lowering the sound level by 4 dB. These findings underscore the public health value of urban greenspaces. Other research has shown that increased greenspace availability in urban environments has the potential to reduce noise annoyance [2]. For example, meeting WHO recommendations for access to ≥ 0.5-hectare greenspaces within 300 meters or a 5-minute walk from home has the potential to reduce the proportion of people highly annoyed by traffic noise by 1.1% [2]. Moreover, a 10% increase in greenspace in cities with over 100,000 residents has the potential to lower the number of highly annoyed individuals by 9.6% [2]. Notably, dense vegetation can also function as a physical barrier that attenuates road traffic noise [100]. This emphasizes the importance of integrating greenspaces into city design to enhance public health and wellbeing.

#### 5.1.2. Repetitive negative thinking.

Contrary to our hypotheses, walking in forests did not lead to a stronger decrease in RNT compared with walking in urban built environments. This contrasts with previous studies showing a greater decrease in rumination after walks in natural environments compared with walks in busy urban built settings [22,23,71]. However, in line with our results, other studies did not find significant differences in the reduction of rumination when comparing various conditions: a 6-minute video of nature and an urban video [101], 1-minute awe-evoking nature clips vs. mundane nature scenes viewed daily for a week [102], and a 45-minute walk in a large park vs. in a busy shopping street [103]. In the past, rumination has been considered as both a trait-like response to negative mood states and a temporary, state-like cognitive response [104]. In investigating a 50-minute [22] and a 90-minute [71] walk, two of the previous studies that indicated differences in the change in rumination between urban and natural environments examined the effect of longer walks on rumination using a trait-like measure, the Rumination Reflection Questionnaire. Lopes et al. [23] focused on a 30-minute walk, using a state measure of rumination (Brief State Rumination Inventory). In our study, a trait measure of RNT (perseverative thinking questionnaire) was used, as the aim was to assess differences between how participants typically think about negative experiences or problems *in everyday life* and how they thought about them during a 30-minute walk. A state-like measure was not suitable to assess what typically applies in daily life. However, given the brief, one-time walk in the present study, capturing changes in trait RNT can be challenging, requiring a cautious interpretation of the results. A potential change in RNT might be more likely to affect state measures, which are more dependent on situational cues. Thus, a state rumination measure like the Brief State Rumination Inventory [104] may be more useful in the context of short-term interventions like the walking intervention applied here.

Interestingly, RNT decreased among participants in both forest and urban built environments. The decrease ranged from 32.79% to 37.57% across conditions, indicating that even trait questionnaires can capture changes resulting from a 30-minute walk. Consistent with this result, Golding et al. [105] did not find a reduction in rumination after exposure to photographs of nature scenes and found state rumination to decrease over time in all three compared conditions (nature images vs. urban images vs. no images), regardless of the type of environment. It is likely that in our study, RNT decreased in both environments due to the general benefits of walking. This aligns with prior research showing that physical activity negatively predicts rumination [106] and that exercise reduces rumination [107]. However, since an inactive control was not included in this study, the assumption that RNT decreased in all conditions due to walking itself remains tentative. Future studies should include an inactive control to isolate the effects of physical activity due to walking from other potentially influential factors.

Previous research has reported reductions in rumination following in situ exposure to greenspaces lasting between 30 [23] and 90 minutes [71]. In terms of exposure duration, the time might have been rather short to elicit pronounced effects (although Lopes et al. [23] did find effects for the same exposure time). Nevertheless, our finding of decreased RNT in both forest and urban built environments after the walk suggests that the absence of environmental differences in our study is unlikely to be attributable to insufficient exposure duration. Our findings contrast those of Lopes et al. [23], who observed greater reductions in rumination after 30-minute walks in urban parks compared to walking along a city transect devoid of natural elements. Thus, future studies should examine whether longer exposure periods yield stronger effects. In summary, no significant effects were observed in our study, which involved a 30-minute exposure and the use of trait-like measures. Nevertheless, these findings should be interpreted with caution given the study’s limitations (see next chapter).

Contrary to our hypotheses, we also did not find an association of road traffic noise exposure (or relative quiet time) with change in RNT during the walk. While road traffic noise has been shown to negatively affect the perceived restorativeness of environments people walk in and recovery-related factors like restoration [11] on noise annoyance as found here, an effect of the same magnitude of road traffic noise on RNT was not found in the present study. This is the first study involving an assessment of the effects of road traffic noise and relative quiet time (free from road traffic noise) on RNT and a trait-like measure of repetitive negative thinking. Future research should investigate whether this finding holds when a state-based measure of RNT is used.

#### 5.1.3. Connectedness with non-human nature.

In the current paper, connectedness with non-human nature encompasses nature relatedness and love and care for nature. Contrary to our expectations, walking in forests did not result in a greater increase in nature relatedness or love and care for nature compared with walking in urban built environments. This contrasts with findings of Weinstein et al. [108], who reported greater connectedness to nature after participants were immersed in a virtual natural environment compared with a non-natural built one. The findings further contrast with results of Mayer et al. [39], who found that participants who spent 15 minutes in a natural environment reported greater state connectedness to nature than those in an urban setting, after accounting for participants trait connectedness. However, both studies used a different scale (connectedness to nature scale) to measure connectedness than applied in the present study (NR-6). Notably, Mayer et al. [39] adapted the scale for the post-exposure measurement to reflect state connectedness (right now..., at this moment...). In our study, participants were instructed to evaluate statements pre-walk based on general applicability. Post-walk, the NR-6 instruction was slightly altered to emphasize the current moment (“...*currently* apply to you?”). The items themselves were not reformulated for either scale. This approach likely reflects a more trait-like connectedness rather than state connectedness. Capturing changes in trait connectedness over a short, 30-minute exposure may be inherently challenging, as changes are more likely to manifest in state connectedness. This is supported by studies where, like in our study, the Nature Relatedness Scale was used and no difference in connectedness changes was found between participants exposed to natural and built environments for 20 minutes [55] or no increase in nature relatedness was found after participants viewed a video of a forest or were immersed in a virtual reality forest for under 7 minutes [109]. To explore this further, future studies should incorporate both trait and state measures of connectedness to facilitate direct comparisons.

When comparing our results on connectedness with non-human nature with literature, one should also consider that (i) longer or more frequent exposures might produce measurable effects, and (ii) Lumber et al. [55] reported that connectedness increased only when participants engaged in additional immersive activities while walking in nature. It thus appears that nature exposure may be more effective at enhancing connectedness when paired with such activities. For examples of such immersing activities, see, e.g., Weinstein et al. [108].

Contrary to our hypotheses, we also found no significant association between road traffic noise or relative quiet time and an increased sense of nature relatedness or love and care for nature. Previous studies have primarily been focused on identifying environmental factors that facilitate connectedness, rather than those that hinder it. By examining the effect of road traffic noise in the present study, we addressed only one of many potential constraints on nature connectedness. Following the constrained restoration concept [64,65], future research should explore additional environmental factors, such as litter or weather, as well as non-environmental aspects.

### 5.2. Open-ended questions

While many people go on walks in their everyday life to reduce stress and experience calm [25], only a few studies have involved explicitly investigating how people’s thoughts change when they go for a walk. Our study revealed that participants most frequently reported a calming effect, followed by notions that their thoughts became clearer, more organized or more sorted and shifted positively. Reports of thoughts becoming calmer or more relaxed, clearer, more structured and less intrusive align with ART and its premise that focused attention is regenerated. Mentions of calmer or more relaxed thoughts suggest a mental shift away from effortful cognition, consistent with the ART component of *being away*, which denotes psychological distance from everyday demands and stressful aspects of everyday life. This suggests that walking can facilitate mental disengagement from routine concerns, consistent with the study’s quantitative findings of reduced RNT in both forest and urban environments, regardless of the setting. Some participants also reported that their attention shifted toward their surroundings, aligning with the ART component of *soft fascination*, where natural or visually pleasing environments effortlessly capture attention. Since participants responded to the open question independently from the environment they walked in, this may also hint at a potential of pleasant urban built environments to promote restorative processes.

Mentions of thoughts becoming more positive, more focused on nice things, more solution-oriented and more constructive suggest a shift in the valence of thoughts and a positive cognitive shift associated with more flexibility and openness. Further, descriptions of thoughts flowing freely, drifting, expanding and fading with natural ease indicate that walking enables a state in which the mind can wander without constraint, offering mental space and the freedom to reflect without a specific goal. Reports that walking led to a relativization of thoughts, indicating that problems or concerns began to feel smaller or less absolute, suggest that the act of walking provides mental distance from immediate concerns, allowing individuals to reframe their thoughts in a broader context and see them as part of a larger picture, rather than as overwhelming or central. However, it is also important to highlight that several participants reported no change in thoughts, indicating that the effects of walking vary between individuals.

From a salutogenic perspective, our findings indicate that walking may function as a generalized resistance resource, as conceived by Antonovsky [14]. By enhancing the manageability of demands, walking appears to support self-regulation and contribute to people’s ability to maintain a sense of coherence.

Similar findings were found for the influence of walking on thought patterns and problem-solving compared with daily life. Some participants noted that walking led to practical problem-solving, suggesting that it may actively aid in solution finding. Others reported that walking let their problems seem less severe, promoting a shift in perspective. Additionally, participants mentioned that walking calmed their thoughts and provided distance from everyday issues, helping them process problems more effectively. Beyond the above-mentioned explanations, this highlights that walking can support restoration by providing a relative absence of demands (e.g., everyday problems and social demands like mobile phones), which opens a space for reflection.

### 5.3. Strengths and limitations

This trial has several methodological strengths, including the use of an active control group (walking in urban built environments) and a longitudinal and rigorous study design. Further, the study is among the first conducted in real-world settings to systematically account for road traffic noise across different environments as a potential influence on both connectedness with non-human nature and RNT. Nevertheless, some limitations should be acknowledged.

First, the measures of RNT and connectedness with non-human nature were more trait-like than state-like. For RNT, this choice was made because the primary interest was in the difference between how participants typically think about negative experiences or problems *in everyday life* and how they thought about them during a 30-minute walk; here, a state-like measure was not suitable. However, given the brief, one-time walk in our study, changes may have been more likely to manifest in state measures. For the connectedness measures, instructions were partly altered to emphasize the current moment, but the items themselves were not reformulated. Second, we acknowledge the challenge of defining an appropriate comparison environment for evaluating the effects of greenspace exposure, as there is no truly neutral environmental baseline. Further, our study did not include an inactive control condition (e.g., sitting on a bench). This makes it difficult to disentangle the effects of physical activity from those of environmental exposure. However, it is unlikely that potential benefits were masked by disturbing road traffic noise. If this were the case, we would expect a greater reduction in RNT and a greater increase in connectedness measures under low-road traffic noise conditions compared to high road traffic noise conditions, which is not supported by the data. Nevertheless, future research should incorporate an inactive control to disentangle the effects of physical activity from those of environmental exposure. Third, to our knowledge, no previous study with a comparable design has used the same outcome measures as those in the present study, making it difficult to estimate expected effect sizes. In the absence of comparable studies assessing these outcomes, we based our power calculations and analytical approach on Schaupp et al. [11], who used Restoration Outcome Scores from a study by Tyrväinen et al. [9]. However, since no established effect sizes are available for RNT, nature relatedness or love and care for nature, it is possible that the true effect sizes are smaller than those for Restoration Outcome Scores. If this is the case, the present study may have been underpowered to detect such smaller effects, and a larger sample size would be required to achieve sufficient statistical power. Nonetheless, given that the current sample was already relatively large (n = 354), any additional effects detectable only with substantially larger samples would likely be small in magnitude and of limited practical relevance. Fourth, the 30-minute exposure duration may have been relatively short to produce pronounced effects. Although previous research has demonstrated benefits for comparable exposure lengths (e.g., Lopes et al. [23]), it remains possible that a longer duration would amplify restorative outcomes or reveal differences between environments. Future studies should therefore explore the effects of extended exposure periods. Finally, other potential confounders such as humidity, air quality and the quality of social interactions during the experiment were not captured, although the seasonal setting of the experiments (afternoons between May and October) should have kept this confounding moderate.

## 6. Conclusion

Overall, this study provided strong evidence for lower road traffic noise annoyance in forests than in urban built environments, in lower road traffic noise vs. higher road traffic noise settings, and in areas with more compared with less relative quiet time. These findings emphasize (1) the importance of limiting road traffic noise as a key acoustic requirement for restorative greenspaces and (2) the relevance of incorporating and protecting quiet areas, undisturbed by unwanted noise, into urban planning to mitigate noise annoyance and promote more liveable cities. Moreover, the study highlights the relevance of quiet intervals and temporal patterns in shaping perceived annoyance, extending beyond average noise measurements. On a practical level, the findings underscore the need for targeted noise mitigation strategies, especially around urban forests, to preserve their restorative potential.

Neither walking in forests, nor walking in environments with lower road traffic noise or more relative quiet time led to a greater reduction in RNT or a stronger increase in nature relatedness or love and care for nature compared with walks in urban built environments. However, methodological refinements—such as incorporating immersive activities during walks in greenspaces, employing state measures and extending the exposure duration—may more effectively facilitate changes. Nevertheless, RNT decreased among participants in both forests and urban built environments, likely due to the general benefits of walking, independent of the environment. This observation aligns with the findings from the open-ended questions on how walking influences thought patterns and problem-solving, specifically with mentions of calmer, more relaxed, more positive, more solution-oriented and less intrusive thoughts. In conclusion, while walking in greenspaces may generally improve wellbeing and mental health, these benefits may be enhanced by adequate noise control in restorative environments.

## Supporting information

S1 File CONSORT checklist. (PDF)

S2 File Research questions and hypotheses. (PDF)

S3 File Formulas for noise calculations. (PDF)

S4 File Results of the linear mixed-effects models with traffic noise included as sound exposure level and as relative quiet time. (PDF)

S5 File Results of the linear mixed-effects models including covariates. (PDF)

S6 File Coding scheme for “How thoughts change when you go for a walk”. (PDF)

S7 File Coding scheme for “How does going for a walk help you to think through personal problems?”. (PDF)

S8 File Original protocol. (PDF)
