# Supplementary material for: Road traffic noise affects annoyance during urban built and forest walks, but not repetitive negative thinking or connectedness with non-human nature: A randomized controlled trial
Source: PLoS One. 2026 Mar 18;21(3):e0342906. doi: 10.1371/journal.pone.0342906 (PMC12998852; doi:10.1371/journal.pone.0342906)
Supplement: S2 File — (PDF) [file pone.0342906.s002.pdf]

S2. Research questions and hypotheses

Research question RQ1: Compared with walking in an urban built environment, is walking in a forest associated with lower noise annoyance due to road traffic noise, a stronger increase in nature relatedness and in love and care for nature, and a stronger decrease in repetitive negative thinking (RNT)?

Hypothesis H1: Compared with walking in an urban built environment, walking in a forest is associated with lower noise annoyance due to road traffic noise, a stronger increase in nature relatedness and in love and care for nature, and a stronger decrease in RNT.

RQ2: Compared with walking in an environment with high traffic noise, is walking in an environment with low traffic noise associated with lower noise annoyance due to road traffic noise, a stronger increase in nature relatedness and in love and care for nature, and a stronger decrease in RNT?

H2: Compared with walking in an environment with high traffic noise, walking in an environment with low traffic noise is associated with lower noise annoyance due to road traffic noise, a stronger increase in nature relatedness and in love and care for nature, and a stronger decrease in RNT.

RQ3: Compared with walking in urban built environments with high road traffic noise, is walking in urban built environments with low road traffic noise associated with lower noise annoyance, a stronger increase in nature relatedness and in love and care for nature, and a stronger decrease in RNT?

H3: Compared with walking in urban built environments with high road traffic noise, walking in urban built environments with low road traffic noise is associated with lower noise annoyance, a stronger increase in nature relatedness and in love and care for nature, and a stronger decrease in RNT.

RQ4: Compared with walking in urban forests with high road traffic noise, is walking in urban forests with low road traffic noise associated with lower noise annoyance, a stronger increase in nature relatedness and in love and care for nature, and a stronger decrease in RNT?

H4: Compared with walking in urban forests with high road traffic noise, walking in urban forests with low road traffic noise is associated with lower noise annoyance, a stronger increase in nature relatedness and in love and care for nature, and a stronger decrease in RNT.

RQ5: What is the effect of 30-minute walks in urban forests on the outcome variables compared with walking in urban built settings, depending on the noise in the settings?

RQ6: How do participants' thoughts change when going for a walk?

RQ7: How does walking help individuals to think through personal problems?
