# Supplementary material for: Road traffic noise affects annoyance during urban built and forest walks, but not repetitive negative thinking or connectedness with non-human nature: A randomized controlled trial
Source: PLoS One. 2026 Mar 18;21(3):e0342906. doi: 10.1371/journal.pone.0342906 (PMC12998852; doi:10.1371/journal.pone.0342906)
Supplement: S3 File — (PDF) [file pone.0342906.s003.pdf]

S3. Formulas for noise calculations

The sound exposure level (L_{AE}) during a period with dominant traffic noise was calculated from the equivalent continuous A-weighted sound pressure level (L_{Aeq}) during that period with dominant road traffic noise as:

$$L_{AE\_tnd\_s} = L_{Aeq\_tnd\_s} + log_{10}(T),$$

with

T= Duration of a single period in which traffic noise was dominant in seconds

$L_{AE\_tnd\_s}$ = sound exposure level of a single period with dominant road traffic noise

$L_{Aeq\_tnd\_s}$ = equivalent continuous A-weighted sound pressure level of a single period with dominant road traffic noise

The cumulative L_{AE} for all periods with dominant traffic noise during a walk was calculated from the individual L_{AE} of each period with dominant road traffic noise using the energetic sum, as:

$$L_{AE\_tnd\_c} = 10 * log_{10}(10^{L1/10} + 10^{L2/10} + \ldots 10^{Li/10}),$$

with

$L_{AE\_tnd\_c}$ = cumulative sound exposure level of all periods with dominant road traffic noise

L1= L_{AE} of a single period 1 in which road traffic noise was dominant

Li= L_{AE} of a single period i in which road traffic noise was dominant

i = number of periods with dominating road traffic noise

Similarly, the cumulative L_{AE} during the cumulative time when road traffic noise was not dominant (sum of all respective periods) was calculated as

$$L_{AE\_quiet\_c} = 30 + log_{10}(T),$$

with

$L_{AE\_quiet\_c}$ = cumulative sound exposure level of all periods when road traffic noise was not dominant

T = Cumulative duration in seconds for times when traffic noise was not dominant within a walk

30 = L_{Aeq} of the time periods when road traffic noise was not dominant, set to 30 dBA.

Further, the L_{AE} of the cumulative periods with dominant road noise ($L_{AE\_tnd\_c}$) and cumulative periods when traffic noise was not dominant ($L_{AE\_quiet\_c}$) was calculated as

$$L_{AE\_tot} = 10 * log_{10}(10^{L_{AE\_tnd\_c/10}} + 10^{L_{AE\_quiet\_c/10}}).$$

With

L_{AE_tot} = L_{AE} cumulative sound exposure lof the whole walk, including times with and without dominant road traffic noise

Finally, the L_{Aeq} of the cumulative periods with dominant road noise and cumulative periods when traffic noise was not dominant (L_{Aeq_tot}) was calculated from L_{AE_tot} as:

$$L_{Aeq\_tot} = L_{AE\_quiet\_c} - 10 * log_{10}(T)$$

with T= total duration of walk in seconds
