# Supplementary material for: Road traffic noise affects annoyance during urban built and forest walks, but not repetitive negative thinking or connectedness with non-human nature: A randomized controlled trial
Source: PLoS One. 2026 Mar 18;21(3):e0342906. doi: 10.1371/journal.pone.0342906 (PMC12998852; doi:10.1371/journal.pone.0342906)
Supplement: S4 File — (PDF) [file pone.0342906.s004.pdf]

S4. Results of the linear mixed-effects models with traffic noise included as sound exposure level and as relative quiet time

Results of the linear mixed-effects models with traffic noise included as sound exposure level (L_{AE})

S1–S4 Tables and S1 Fig. show the results of the linear mixed-effects models with traffic noise included as a continuous predictor (L_{AE}). Estimates show the change in the outcomes per dB increase/decrease in L_{AE} (except for noise annoyance, which was only assessed after the walk).

S1 Table. Effects of the model with L_{AE} on noise annoyance

	Estimate	SE[1]	Lower CI[2]	Upper CI[2]	p
Effect of environment averaged across L_{AE}	-0.237	0.408	-1.038	0.563	0.720
Effect of L_{AE} averaged across environment conditions	0.261	0.020	0.221	0.300	< 0.001
Effect of L_{AE} in urban built conditions	0.199	0.038	0.125	0.274	< 0.001
Effect of L_{AE} in forest conditions	0.306	0.021	0.265	0.347	< 0.001
Environment: L_{AE} interaction	0.107	0.043	0.022	0.192	0.014

[1]SE = standard error. [2]CI = 95% confidence interval

S1 Table and S1A Fig. indicate little evidence for an association between environment and noise annoyance, with confidence intervals including zero. However, strong evidence for an association between L_{AE} averaged across environment conditions and road traffic noise annoyance was found, showing higher road traffic noise to be associated with increased noise annoyance. Additionally, S1 Table shows strong evidence for an interaction between environment and noise on noise annoyance. The association between traffic noise and noise annoyance was considerably greater in forests than in urban built environments.

S2 Table. Effects of the model with L_{AE} on change in repetitive negative thinking

	Estimate	SE[1]	Lower CI[2]	Upper CI[2]	p
Effect of environment averaged across L_{AE}	-0.098	0.112	-0.317	0.121	0.381
Effect of L_{AE} averaged across environment conditions	0.000	0.005	-0.010	0.011	0.985
Effect of L_{AE} in urban built conditions	0.001	0.010	-0.020	0.021	0.947
Effect of L_{AE} in forest conditions	-0.001	0.006	-0.012	0.010	0.904
Environment: L_{AE} interaction	-0.001	0.012	-0.025	0.022	0.908

[1]SE = standard error. [2]CI = 95% confidence interval

According to S2 Table and S1B Fig., considerable statistical uncertainty is found for an association of environment and road traffic noise with RNT and for an interaction between environment and traffic noise. While the point estimates suggest that RNT decreased in both conditions, the decrease was less pronounced in forests than in urban built environments and confidence intervals included zero. Given the imprecision of the estimate, there is no evidence of an association. The point estimates for the association of L_{AE} with RNT averaged across environment conditions, as well as the association between traffic noise and RNT in the urban built and forest conditions were zero or close to zero, suggesting a negligible or absent association. Given the imprecision of the estimate, there is no evidence of an association of L_{AE} with RNT, or of an interaction between environment and traffic noise on RNT.

S3 Table. Effects of the model with L_{AE} on change in nature relatedness

	Estimate	SE[1]	Lower CI[2]	Upper CI[2]	p
Effect of environment averaged across L_{AE}	-0.065	0.053	-0.168	0.038	0.219
Effect of L_{AE} averaged across environment conditions	0.000	0.003	-0.005	0.005	0.953
Effect of L_{AE} in urban built conditions	-0.001	0.005	-0.011	0.008	0.796
Effect of L_{AE} in forest conditions	0.001	0.003	-0.004	0.006	0.656
Environment: L_{AE} interaction	0.002	0.006	-0.008	0.013	0.659

[1]SE = standard error. [2]CI = 95% confidence interval

S3 Table and S1C Fig. indicate substantial statistical uncertainty for an association of environment with nature relatedness. While the point estimates suggest that nature relatedness increased marginally in forests, compared with a marginal decrease in urban built environments, the confidence intervals include zero, indicating that the true association may be negligible or absent. Given the imprecision of the estimate, there is no evidence of an association. Little evidence of an association between L_{AE} and nature relatedness of of an interaction between environment and traffic noise on nature relatedness was found.

S4 Table. Effects of the model with L_{AE} on change in love and care for nature

	Estimate	SE[1]	Lower CI[2]	Upper CI[2]	p
Effect of environment averaged across L_{AE}	-0.008	0.078	-0.161	0.144	0.914
Effect of L_{AE} averaged across environment conditions	0.003	0.004	-0.005	0.010	0.458
Effect of L_{AE} in urban built conditions	0.005	0.007	-0.009	0.019	0.492
Effect of L_{AE} in forest conditions	0.001	0.004	-0.007	0.009	0.750
Environment: L_{AE} interaction	-0.004	0.008	-0.020	0.013	0.654

[1]SE = standard error. [2]CI = 95% confidence interval

S4 Table and S1D Fig. indicate substantial statistical uncertainty for an association of environment and traffic noise with changes in the Love and Care for Nature Scale. The point estimates suggest that love and care for nature decreased slightly in all conditions, that the decrease was less pronounced in forests, compared with urban built environments, and that love and care for nature decreased slightly with increasing traffic noise. However, the confidence intervals include zero, indicating that the association is be negligible or absent. Given the imprecision of the estimate, there is no evidence of an association. Additionally, no evidence for an interaction between environment and traffic noise on love and care for nature was found.

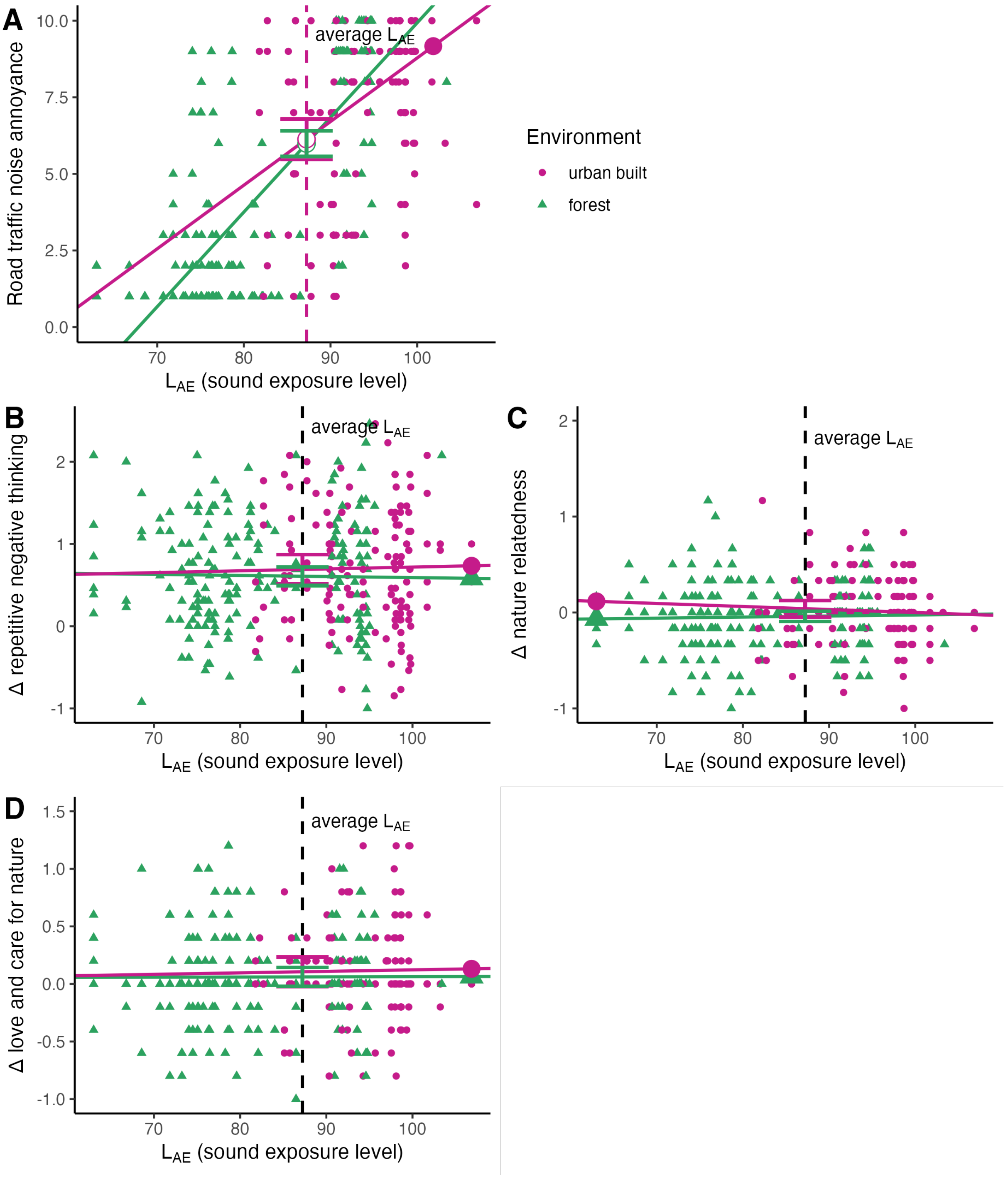

S1 Fig. Results for noise annoyance (range 0–10), repetitive negative thinking (range 0–4), nature relatedness (range 1–5), and love and care for nature (range 1–7). Effect of environment and noise exposure (L_{AE}) with a post-hoc test at mean L_{AE}. Panels B–D depict differences (delta before minus after). A positive delta score for repetitive negative thinking reflects a decrease in repetitive negative thinking; a positive delta score for nature relatedness indicates a decrease in nature relatedness; and positive delta score for love and care for nature reflects a decrease in love and care for nature.

Results of the linear mixed-effects models with traffic noise included as relative quiet time

S5–S8 Tables and S2 Fig. show the results of the linear mixed-effects models with traffic noise included as relative quiet time (RQT).

S5 Table. Effects of the model with RQT on noise annoyance

	Estimate	SE[1]	Lower CI[2]	Upper CI[2]	p
Effect of environment averaged across RQT[3]	-1.537	0.378	-2.277	-0.797	< 0.001
Effect of RQT averaged across environment conditions	-0.064	0.006	-0.076	-0.051	< 0.001
Effect of RQT in urban built conditions	-0.049	0.013	-0.073	-0.024	< 0.001
Effect of RQT in forest conditions	-0.075	0.006	-0.087	-0.063	< 0.001
Interaction environment: RQT	-0.026	0.014	-0.054	0.001	0.058

[1]SE = standard error. [2]CI = 95% confidence interval. [3]Reference category for environment = urban. N=341.

S5 Table and S2A Fig. show strong evidence for an association of environment and traffic noise with noise annoyance. Road traffic noise annoyance was moderately lower in forests settings, than in urban built environments and noise annoyance decreased with increasing RQT. The confidence intervals for the interaction effect between environment and RQT include zero, but the upper confidence interval is very close to zero. Thus, while the interaction between environment and RQT on noise annoyance is not statistically significant at the 0.05 level ($p = 0.058$), it approaches significance, and the direction of the association suggests that RQT may have a stronger association with reduced noise annoyance in forests than in urban environments. The confidence interval narrowly includes zero, which indicates some uncertainty but still alignment with the hypothesized effect.

S6 Table. Effects of the model with RQT on change in repetitive negative thinking

	Estimate	SE[1]	Lower CI[2]	Upper CI[2]	p
Effect of environment averaged across RQT[3]	-0.086	0.097	-0.276	0.104	0.373
Effect of RQT averaged across environment conditions	0.000	0.002	-0.004	0.003	0.797
Effect of RQT in urban built conditions	-0.001	0.003	-0.007	0.006	0.858
Effect of RQT in forest conditions	-0.000	0.002	-0.003	0.003	0.843
Interaction environment: RQT	0.000	0.004	-0.007	0.007	0.939

[1]SE = standard error. [2]CI = 95% confidence interval. [3]Reference category for environment = urban. N=341.

S6 Table and S2B Fig. indicate little evidence for an association of environment and RQT with RNT. Additionally, little evidence for an interaction between environment and RQT on RNT was found.

S7 Table. Effects of the model with RQT on change in nature relatedness

	Estimate	SE[1]	Lower CI[2]	Upper CI[2]	p
Effect of environment averaged across RQT[3]	-0.031	0.046	-0.120	0.058	0.494
Effect of RQT averaged across environment conditions[4]	-0.001	0.001	-0.002	0.001	0.217
Effect of RQT in urban built conditions	-0.002	0.002	-0.005	0.001	0.308
Effect of RQT in forest conditions	-0.001	0.001	-0.002	0.001	0.484
Interaction environment: RQT	0.001	0.002	-0.002	0.004	0.535

[1]SE = standard error. [2]CI = 95% confidence interval. [3]Reference category for environment = urban. N=341.

S7 Table and S2C Fig. indicate little evidence for an association of environment and RQT

with nature relatedness. Furthermore, little evidence for an interaction between environment and RQT on nature relatedness was found.

S8 Table. Effects of the model with RQT on change in love and care for nature

	Estimate	SE[1]	Lower CI[2]	Upper CI[2]	p
Effect of environment averaged across RQT[3]	0.016	0.067	-0.117	0.148	0.818
Effect of RQT averaged across environment conditions	-0.002	0.001	-0.004	0.000	0.081
Effect of RQT in urban built conditions	-0.003	0.002	-0.008	0.001	0.136
Effect of RQT in forest conditions	-0.001	0.001	-0.003	0.001	0.362
Interaction environment: RQT	0.002	0.002	-0.003	0.007	0.340

[1]SE = standard error. [2]CI = 95% confidence interval. [3]Reference category for environment = urban. N=341.

S8 Table and S2D Fig. show little evidence for an association between environment and Love and care for nature. Love and care for nature was hardly affected by RQT. Some statistical uncertainty was found for this marginal association of RQT with love and care for nature. The point estimates indicate that love and care for nature increased marginally with increasing RQT. However, the upper confidence interval for this association was zero, suggesting that the true association may also go in the other direction or be negligible. This result should therefore be interpreted with caution and rather be seen as negligible. Additionally, little evidence for an interaction between environment and RQT was found.

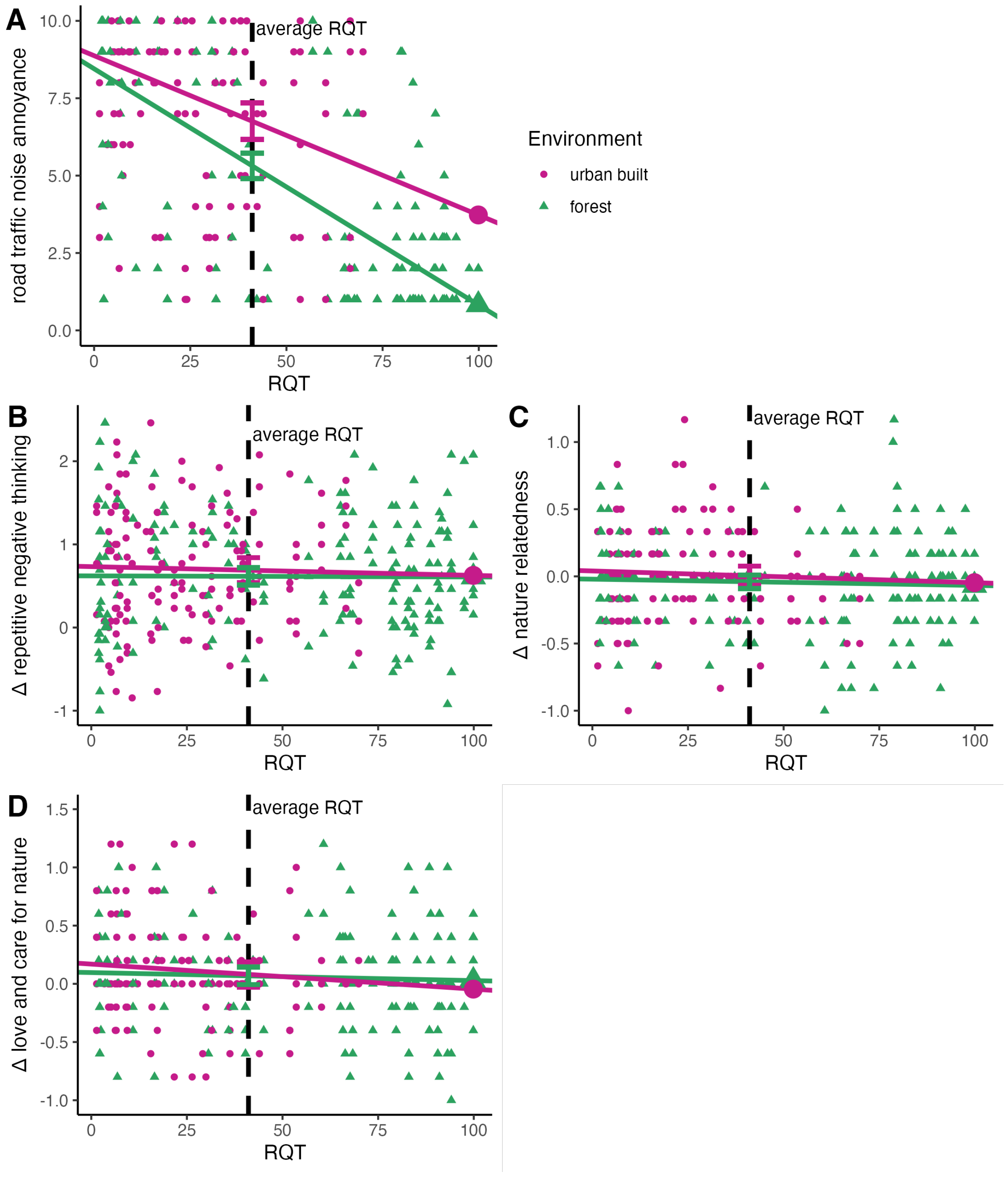

S2 Fig. Results for noise annoyance (range 0–10), repetitive negative thinking (range 0–4), nature relatedness (range 1–5), and love and care for nature (range 1–7). Effect of environment and relative quiet time with a post-hoc test at mean RQT. Panels B–D depict differences (delta before minus after). A positive delta score for repetitive negative thinking reflect a decrease in repetitive negative thinking; a positive delta score for nature relatedness indicates a decrease in nature relatedness; and positive delta score for love and care for nature reflects a decrease in love and care for nature.
