# Supplementary material for: Road traffic noise affects annoyance during urban built and forest walks, but not repetitive negative thinking or connectedness with non-human nature: A randomized controlled trial
Source: PLoS One. 2026 Mar 18;21(3):e0342906. doi: 10.1371/journal.pone.0342906 (PMC12998852; doi:10.1371/journal.pone.0342906)
Supplement: S5 File — (PDF) [file pone.0342906.s005.pdf]

S5. Results of the linear mixed-effects models including covariates

Results of the linear mixed-effects models with dummy-coded traffic noise

S9 Table indicates strong evidence for an association of environment averaged across traffic noise conditions with noise annoyance and a strong association between road traffic noise averaged across environment conditions and noise annoyance. Road traffic noise annoyance was lower in forest settings, than in urban built environments and higher in environments with high traffic noise, compared with that in settings with low traffic noise. Additionally, strong evidence for an interaction between environment and noise on noise annoyance was found. Road traffic noise showed a stronger association with noise annoyance in forest environments compared with urban built environments. Frequency of greenspace visits was a significant covariate, with participants indicating more frequent greenspace visits showing greater noise annoyance. Little evidence was found for an effect of the other covariates.

S9 Table. Effects of the model with dummy-coded noise on noise annoyance

	Estimate	SE[1]	Lower CI[2]	Upper CI[2]	p
Effect of environment averaged across noise conditions[3]	-2.165	0.270	-2.696	-1.634	< 0.001
Effect of noise averaged across environment conditions[3]	4.706	0.267	4.181	5.231	< 0.001
Effect of noise in urban built conditions	2.548	0.399	1.762	3.333	< 0.001
Effect of noise in forest conditions	6.864	0.358	6.159	7.569	< 0.001
Age	-0.013	0.008	-0.030	0.003	0.115
Gender (male)[4]	0.174	0.279	-0.375	0.724	0.533
Highest education high school, teacher training college[5]	0.643	0.485	-0.311	1.598	0.186
Highest education higher technical or vocational education, art school[5]	0.876	0.513	-0.134	1.885	0.089
Highest education University of Applied Sciences or University of Teacher Education[5]	0.676	0.401	-0.113	1.465	0.093
Highest education other[5]	1.302	1.742	-2.126	4.729	0.456
Frequency of greenspace visits 2–3 times a month[6]	1.711	0.802	0.134	3.289	0.034
Frequency of greenspace visits once a week[6]	2.253	0.753	0.771	3.735	0.003
Frequency of greenspace visits several times a week[6]	2.267	0.736	0.820	3.715	0.002
Frequency of greenspace visits daily[6]	2.430	0.818	0.820	4.040	0.003
Interaction environment: noise	4.316	0.539	3.256	5.377	< 0.001

[1]SE = standard error. [2]CI = confidence interval. [3]Calculated using Custom Contrasts, reference category for environment = urban, reference category for noise = low traffic noise. [4]Reference for gender = female. [5]Reference for highest education = primary/secondary modern/district school/lower grammar school & apprenticeship, vocational school, business school; trade school. [6]Reference for frequency of greenspace visits = fewer than 2–3 times a month.

S10 Table shows considerable statistical uncertainty for an association of environment and road traffic noise with RNT. The point estimates suggest that RNT decreased less during forest walks than in urban built environments. RNT was hardly affected by road traffic noise; the confidence intervals are very large and include zero, indicating that the association is negligible or even absent. S10 Table further indicates substantial statistical uncertainty for an interaction between environment and noise. Little evidence was found for effects of all the covariates.

S10 Table. Effects on change in repetitive negative thinking

	Estimate	SE[1]	Lower CI[2]	Upper CI[2]	p
Effect of environment averaged across noise conditions[3]	-0.075	0.085	-0.242	0.092	0.379
Effect of noise averaged across environment conditions[3]	0.002	0.084	-0.164	0.167	0.983
Effect of noise in urban built conditions	-0.004	0.126	-0.252	0.243	0.973
Effect of noise in forest conditions	0.008	0.113	-0.214	0.230	0.945
Age	-0.001	0.003	-0.006	0.004	0.778
Gender (male)[4]	-0.118	0.088	-0.291	0.055	0.179
Highest education high school, teacher training college[5]	-0.060	0.153	-0.360	0.241	0.696
Highest education higher technical or vocational education, art school[5]	0.064	0.162	-0.254	0.382	0.692
Highest education University of Applied Sciences or University of Teacher Education[5]	0.082	0.126	-0.166	0.331	0.515
Highest education other[5]	0.324	0.549	-0.755	1.403	0.555
Frequency of greenspace visits 2–3 times a month[6]	-0.013	0.252	-0.510	0.483	0.959
Frequency of greenspace visits once a week[6]	0.076	0.237	-0.391	0.542	0.749
Frequency of greenspace visits several times a week[6]	0.064	0.232	-0.392	0.519	0.784
Frequency of greenspace visits daily[6]	0.061	0.258	-0.446	0.568	0.812
Interaction environment: noise	0.012	0.170	-0.322	0.346	0.943

[1]SE = standard error. [2]CI = confidence interval. [3]Calculated using Custom Contrasts, reference category for environment = urban, reference category for noise = low traffic noise. [4]Reference for gender = female. [5]Reference for highest education = primary/secondary modern/district school/lower grammar school & apprenticeship, vocational school, business school; trade school. [6]Reference for frequency of greenspace visits = fewer than 2–3 times a month.

S11 Table shows substantial statistical uncertainty for an association of environment and noise with nature relatedness. The point estimates suggest that nature relatedness increased more in forests than urban built environments and decreased more in environments with high traffic noise than in those with low traffic noise. The confidence intervals include zero, indicating that the association may be negligible or absent. Given the imprecision of the estimate, there is no evidence of an association. Additionally, no evidence for an interaction between environment and noise on nature relatedness was found. No evidence was found for the effects of all the covariates.

S11 Table. Effects on change in nature relatedness

	Estimate	SE[1]	Lower CI[2]	Upper CI[2]	p
Effect of environment averaged across noise conditions[3]	-0.064	0.040	-0.142	0.015	0.110
Effect of noise averaged across environment conditions[3]	0.003	0.039	-0.074	0.080	0.941
Effect of noise in urban built conditions	-0.031	0.059	-0.147	0.084	0.592
Effect of noise in forest conditions	0.037	0.053	-0.066	0.141	0.480
Age	-0.000	0.001	-0.002	0.002	0.962
Gender (male)[4]	0.055	0.041	-0.026	0.136	0.181
Highest education high school, teacher training college[5]	0.101	0.071	-0.039	0.241	0.157
Highest education higher technical or vocational education, art school[5]	0.115	0.075	-0.034	0.263	0.129
Highest education University of Applied Sciences or University of Teacher Education[5]	0.052	0.059	-0.063	0.168	0.374
Highest education other[5]	-0.459	0.256	-0.962	0.045	0.074
Frequency of greenspace visits 2–3 times a month[6]	-0.106	0.118	-0.338	0.125	0.367
Frequency of greenspace visits once a week[6]	-0.144	0.111	-0.362	0.074	0.194
Frequency of greenspace visits several times a week[6]	-0.149	0.108	-0.362	0.063	0.168
Frequency of greenspace visits daily[6]	-0.125	0.120	-0.362	0.111	0.297
Interaction environment: noise	0.069	0.079	-0.087	0.224	0.386

[1]SE = standard error. [2]CI = confidence interval. [3]Calculated using Custom Contrasts, reference category for environment = urban, reference category for noise = low traffic noise. [4]Reference for gender = female. [5]Reference for highest education = primary/secondary modern/district school/lower grammar school & apprenticeship, vocational school, business school; trade school. [6]Reference for frequency of greenspace visits = fewer than 2–3 times a month.

S12 Table indicates substantial statistical uncertainty for an association of environment and noise with the Love and Care for Nature Scale. The point estimates suggest that love and care

for nature decreased slightly less in forests than in urban built environments and decreased slightly more in environments with high traffic noise than in those with low traffic noise. The confidence intervals include zero, indicating that the association may be negligible or absent. Given the imprecision of the estimate, there is no evidence of an association. Also, no (clear) evidence for an interaction between environment and noise on love and care for nature was found. Strong evidence was found that participants indicating higher technical or vocational education or art school as their highest level of education reported a greater decrease in love and care for nature, compared with those indicating primary or secondary school, modern or district school, lower grammar school, apprenticeship, vocational school, business school or trade school as their highest level of education.

S12 Table. Effects on change in love and care for nature

	Estimate	SE[1]	Lower CI[2]	Upper CI[2]	p
Effect of environment averaged across noise conditions[3]	-0.047	0.058	-0.162	0.067	0.417
Effect of noise averaged across environment conditions[3]	0.061	0.057	-0.052	0.174	0.293
Effect of noise in urban built conditions	0.050	0.086	-0.119	0.219	0.561
Effect of noise in forest conditions	0.071	0.077	-0.081	0.223	0.357
Age	0.003	0.002	-0.001	0.006	0.152
Gender (male)[4]	0.064	0.060	-0.054	0.183	0.285
Highest education high school, teacher training college[5]	0.183	0.104	-0.022	0.389	0.080
Highest education higher technical or vocational education, art school[5]	0.265	0.110	0.048	0.482	0.017
Highest education University of Applied Sciences or University of Teacher Education[5]	0.133	0.086	-0.036	0.303	0.123
Highest education other[5]	-0.365	0.375	-1.103	0.372	0.331
Frequency of greenspace visits 2–3 times a month[6]	0.001	0.173	-0.338	0.341	0.993
Frequency of greenspace visits once a week[6]	0.038	0.162	-0.280	0.357	0.813
Frequency of greenspace visits several times a week[6]	-0.032	0.158	-0.344	0.279	0.838
Frequency of greenspace visits daily[6]	-0.006	0.176	-0.353	0.340	0.973
Interaction environment: noise	0.021	0.116	-0.207	0.249	0.856

[1]SE = standard error. [2]CI = confidence interval. [3]Calculated using Custom Contrasts, reference category for environment = urban, reference category for noise = low traffic noise. [4]Reference for gender = female. [5]Reference for highest education = primary/secondary modern/district school/lower grammar school & apprenticeship, vocational school, business school; trade school. [6]Reference for frequency of greenspace visits = fewer than 2–3 times a month.

Results of the linear mixed-effects models with traffic noise included as sound exposure level (L_{AE})

S13–S16 Tables depict the results of the linear mixed-effects models with traffic noise included as sound exposure level (L_{AE}) with covariates. Estimates show the change in the outcomes per dB increase/decrease in L_{AE} (except for noise annoyance, which was only assessed after the walk). S13 Table indicates strong evidence for a strong association between road traffic noise averaged across environment conditions and noise annoyance. Noise annoyance increased with increasing L_{AE}. No evidence was found for an association of environment with noise annoyance. Strong evidence for an interaction between environment and road traffic noise on noise annoyance was found. The increasing effect of traffic noise on noise annoyance was greater in forests than in urban built environments. Several covariates had a significant effect on noise annoyance: participants giving technical education, vocational education, or art school as their highest level of education reported significantly greater noise annoyance than those giving primary, secondary modern, district school, lower grammar school, apprenticeship, vocational school, business school, or trade school as highest education level. Further, participants who reported that they visit greenspaces more often showed greater noise annoyance during the walk, compared with those who reported that they visit greenspaces fewer than 2-3 times a month.

S13 Table. Effects of model with L_{AE} on noise annoyance

	Estimate	SE[1]	Lower CI[2]	Upper CI[2]	p
Effect of environment averaged across L_{AE}	-0.147	0.412	-0.953	0.660	0.722
Effect of L_{AE} averaged across environment conditions	0.267	0.020	0.227	0.306	< 0.001
Effect of L_{AE} in urban built conditions	0.198	0.038	0.124	0.272	< 0.001
Effect of L_{AE} in forest conditions	0.317	0.021	0.276	0.358	< 0.001
Age	-0.005	0.009	-0.024	0.013	0.565
Gender (male)[3]	-0.098	0.312	-0.712	0.516	0.754
Gender (other)[3]	0.531	1.537	-2.492	3.554	0.730
Highest education high school, teacher training college[4]	0.797	0.538	-0.261	1.854	0.139
Highest education higher technical or vocational education, art school[4]	1.175	0.573	0.047	2.302	0.041
Highest education University of Applied Sciences or University of Teacher Education[4]	0.769	0.445	-0.106	1.645	0.085
Highest education other[4]	2.543	1.952	-1.296	6.382	0.194
Visits to greenspaces 2–3 times a month	1.903	0.899	0.134	3.672	0.035
Visits to greenspaces once a week[5]	2.843	0.848	1.174	4.512	< 0.001
Visits to greenspaces several times a week[5]	2.888	0.828	1.258	4.518	< 0.001
Visits to greenspaces daily[5]	2.448	0.918	0.642	4.254	0.008
Interaction environment: L_{AE}	0.119	0.043	0.034	0.204	0.006

[1]SE=standard error. [2]CI= confidence interval. [3]Referece for gender= female. [4]Reference for highest education= Primary/secondary modern/district school/lower grammar school & apprenticeship, vocational school, business school; trade school. [5]Reference for frequency of greenspace visits = fewer than 2–3 times a month.

S14 Table indicates no evidence for an association of environment with RNT. Substantial statistical uncertainty for a zero association of road traffic noise with RNT and for an interaction effect between environment and noise was found. Little evidence was found for the effects of all the covariates.

S14 Table. Effects of model with L_{AE} on change in repetitive negative thinking

	Estimate	SE[1]	Lower CI[2]	Upper CI[2]	p
Effect of environment averaged across L_{AE}	-0.067	0.115	-0.293	0.159	0.561
Effect of L_{AE} averaged across environment conditions	0.000	0.006	-0.011	0.011	>.999
Effect of L_{AE} in urban built conditions	0.001	0.011	-0.020	0.022	0.916
Effect of L_{AE} in forest conditions	-0.001	0.006	-0.012	0.011	0.887
Age	-0.001	0.003	-0.006	0.004	0.733
Gender (male)[3]	-0.118	0.088	-0.291	0.054	0.178
Gender (other)[3]	0.469	0.431	-0.379	1.317	0.277
Highest education high school, teacher training college[4]	-0.069	0.151	-0.366	0.228	0.647
Highest education higher technical or vocational education, art school[4]	0.060	0.161	-0.256	0.376	0.710
Highest education University of Applied Sciences or University of Teacher Education[4]	0.069	0.125	-0.177	0.315	0.580
Highest education other[4]	0.304	0.547	-0.773	1.381	0.579
Visits to greenspaces 2–3 times a month	-0.006	0.252	-0.503	0.490	0.980
Visits to greenspaces once a week[5]	0.076	0.238	-0.393	0.544	0.751
Visits to greenspaces several times a week[5]	0.057	0.232	-0.400	0.514	0.807
Visits to greenspaces daily[5]	0.058	0.258	-0.449	0.564	0.823
Interaction environment: L_{AE}	-0.002	0.012	-0.026	0.022	0.872

[1]SE=standard error. [2]CI= confidence interval. [3]Referece for gender= female.
[4]Reference for highest education= Primary/secondary modern/district school/lower grammar school & apprenticeship, vocational school, business school; trade school.
[5]Reference for frequency of greenspace visits = fewer than 2–3 times a month.

S15 Table shows little evidence for an association of environment with nature relatedness. The point estimates suggest that nature relatedness increased slightly more in forests, compared with urban built environments. The confidence intervals include zero, indicating that the association may be negligible or absent. Given the imprecision of the estimate, there is no evidence of an association. The point estimate indicated no association of road traffic noise with nature relatedness. Additionally, little evidence for an interaction between environment and L_{AE} on nature relatedness was found. Little evidence was found for the effects of all the covariates.

S15 Table. Effects of model with L_{AE} on change in nature relatedness

	Estimate	SE[1]	Lower CI[2]	Upper CI[2]	p
Effect of environment averaged across L_{AE}	-0.080	0.054	-0.185	0.026	0.140
Effect of L_{AE} averaged across environment conditions	0.000	0.003	-0.005	0.005	0.911
Effect of L_{AE} in urban built conditions	-0.002	0.005	-0.012	0.007	0.649
Effect of L_{AE} in forest conditions	0.001	0.003	-0.004	0.006	0.670
Age	-0.000	0.001	-0.002	0.002	0.964
Gender (male)[3]	0.055	0.041	-0.026	0.135	0.181
Gender (other)[3]	0.151	0.201	-0.244	0.547	0.452
Highest education high school, teacher training college[4]	0.097	0.070	-0.042	0.235	0.170
Highest education higher technical or vocational education, art school[4]	0.114	0.075	-0.033	0.261	0.129
Highest education University of Applied Sciences or University of Teacher Education[4]	0.050	0.058	-0.065	0.164	0.391
Highest education other[4]	-0.457	0.255	-0.959	0.045	0.074
Visits to greenspaces 2–3 times a month	-0.106	0.118	-0.337	0.125	0.367
Visits to greenspaces once a week[5]	-0.142	0.111	-0.361	0.076	0.200
Visits to greenspaces several times a week[5]	-0.150	0.108	-0.363	0.063	0.168
Visits to greenspaces daily[5]	-0.128	0.120	-0.364	0.108	0.287
Interaction environment: L_{AE}	0.003	0.006	-0.008	0.015	0.547

[1]SE=standard error. [2]CI= confidence interval. [3]Referece for gender= female.
[4]Reference for highest education= Primary/secondary modern/district school/lower grammar school & apprenticeship, vocational school, business school; trade school.
[5]Reference for frequency of greenspace visits = fewer than 2–3 times a month.

S16 Table indicates substantial statistical uncertainty for an association of environment and noise with the Love and Care for Nature Scale. The point estimates indicate that love and care for nature increased more in forests, compared with urban built environments, and decreased with increasing L_{AE}. The confidence intervals include zero, indicating that the association may be negligible or absent. Given the imprecision of the estimate, there is no evidence of an association. Additionally, little evidence for an interaction between environment and noise on love and care for nature was found. Strong evidence was found that participants indicating higher technical or vocational education or art school as their highest level of education reported a greater decrease in love and care for nature than those indicating primary or secondary school, modern or district school, lower grammar school, apprenticeship, vocational school, business school or trade school as their highest education level.

S16 Table. Effects of model with L_{AE} on change in love and care for nature

	Estimate	SE[1]	Lower CI[2]	Upper CI[2]	p
Effect of environment averaged across L_{AE}	-0.003	0.080	-0.159	0.154	0.975
Effect of L_{AE} averaged across environment conditions	0.003	0.004	-0.004	0.011	0.422
Effect of L_{AE} in urban built conditions	0.005	0.007	-0.009	0.019	0.493
Effect of L_{AE} in forest conditions	0.002	0.004	-0.006	0.010	0.676
Age	0.002	0.002	-0.001	0.006	0.172
Gender (male)[3]	0.061	0.061	-0.058	0.180	0.314
Gender (other)[3]	-0.191	0.298	-0.777	0.395	0.523
Highest education high school, teacher training college[4]	0.182	0.104	-0.024	0.387	0.083
Highest education higher technical or vocational education, art school[4]	0.266	0.111	0.048	0.485	0.017
Highest education University of Applied Sciences or University of Teacher Education[4]	0.119	0.086	-0.051	0.289	0.169
Highest education other[4]	-0.385	0.378	-1.130	0.359	0.309
Visits to greenspaces 2–3 times a month	0.021	0.174	-0.322	0.364	0.904
Visits to greenspaces once a week[5]	0.042	0.165	-0.282	0.366	0.798
Visits to greenspaces several times a week[5]	-0.036	0.161	-0.352	0.280	0.825
Visits to greenspaces daily[5]	-0.008	0.178	-0.359	0.342	0.963
Interaction environment: L_{AE}	-0.003	0.008	-0.020	0.013	0.691

[1]SE=standard error. [2]CI= confidence interval. [3]Referece for gender= female. [4]Reference for highest education= Primary/secondary modern/district school/lower grammar school & apprenticeship, vocational school, business school; trade school. [5]Reference for frequency of greenspace visits = fewer than 2–3 times a month.

Results of the linear mixed-effects models with traffic noise included as relative quiet time (RQT)

S17–S20 Tables depict the results of the linear mixed-effects models with traffic noise included as RQT with covariates. S17 Table indicates strong evidence for an association of environment with road traffic noise annoyance and between RQT and noise annoyance. Road traffic noise annoyance was lower in forests than in urban built environments. In addition, noise annoyance decreased with increasing RQT. Furthermore, strong evidence for an interaction between environment and RQT on noise annoyance was found. The decreasing effect of RQT noise annoyance was considerably stronger in forests than in urban built environments. Participants who indicated that they visit greenspaces once or several times a week showed greater noise annoyance than those who reported visiting greenspaces fewer than 2–3 times a month.

S17 Table. Effects of model with RQT on noise annoyance

	Estimate	SE[1]	Lower CI[2]	Upper CI[2]	p
Effect of environment averaged across RQT	-1.531	0.385	-2.286	-0.775	< 0.001
Effect of RQT averaged across environment conditions	-0.064	0.006	-0.077	-0.052	< 0.001
Effect of RQT in urban built conditions	-0.047	0.013	-0.072	-0.022	< 0.001
Effect of RQT in forest conditions	-0.077	0.006	-0.089	-0.065	< 0.001
Age	-0.008	0.010	-0.028	0.012	0.425
Gender (male)[3]	-0.268	0.337	-0.931	0.395	0.427
Gender (other)[3]	0.789	1.663	-2.482	4.060	0.635
Highest education high school, teacher training college[4]	0.750	0.580	-0.390	1.891	0.197
Highest education higher technical or vocational education, art school[4]	0.817	0.620	-0.403	2.037	0.189
Highest education University of Applied Sciences or University of Teacher Education[4]	0.836	0.481	-0.110	1.781	0.083
Highest education other[4]	2.165	2.108	-1.982	6.311	0.305
Visits to greenspaces 2–3 times a month	1.188	0.967	-0.714	3.091	0.220
Visits to greenspaces once a week[5]	1.861	0.911	0.069	3.653	0.042
Visits to greenspaces several times a week[5]	1.962	0.890	0.211	3.713	0.028
Visits to greenspaces daily[5]	1.608	0.988	-0.335	3.551	0.104
Interaction environment: RQT	-0.030	0.014	-0.058	-0.002	0.033

[1]SE=standard error. [2]CI= confidence interval. [3]Referece for gender= female. [4]Reference for highest education= Primary/secondary modern/district school/lower grammar school & apprenticeship, vocational school, business school; trade school. [5]Reference for frequency of greenspace visits = less than 2–3 times a month.

S18 Table indicates considerable statistical uncertainty for an association of environment with RNT. In addition, no evidence for an association of road traffic noise with RNT or for an interaction between environment and RQT was found. No evidence was found for the effects of all the covariates.

S18 Table. Effects of model with RQT on repetitive negative thinking

	Estimate	SE[1]	Lower CI[2]	Upper CI[2]	p
Effect of environment averaged across RQT	-0.057	0.100	-0.254	0.139	0.567
Effect of RQT averaged across environment conditions	0.000	0.002	-0.004	0.003	0.783
Effect of RQT in urban built conditions	-0.001	0.003	-0.007	0.006	0.858
Effect of RQT in forest conditions	-0.000	0.002	-0.003	0.003	0.818
Age	-0.001	0.003	-0.006	0.004	0.734
Gender (male)[3]	-0.118	0.088	-0.290	0.054	0.178
Gender (other)[3]	0.471	0.432	-0.379	1.320	0.277
Highest education high school, teacher training college[4]	-0.066	0.151	-0.363	0.230	0.660
Highest education higher technical or vocational education, art school[4]	0.057	0.161	-0.259	0.374	0.722
Highest education University of Applied Sciences or University of Teacher Education[4]	0.071	0.125	-0.174	0.317	0.569
Highest education other[4]	0.301	0.547	-0.776	1.378	0.583
Frequency of greenspace visits 2–3 times a month[5]	-0.002	0.251	-0.496	0.492	0.994
Frequency of greenspace visits once a week[5]	0.082	0.237	-0.384	0.547	0.730
Frequency of greenspace visits several times a week[5]	0.062	0.231	-0.393	0.517	0.789
Frequency of greenspace visits daily[5]	0.062	0.257	-0.442	0.567	0.808
Interaction environment: RQT	0.000	0.004	-0.007	0.007	0.951

[1]SE=standard error. [2]CI= confidence interval. [3]Referece for gender= female. [4]Reference for highest education= Primary/secondary modern/district school/lower grammar school & apprenticeship, vocational school, business school; trade school. [5]Reference for frequency of greenspace visits = fewer than 2–3 times a month.

S19 Table indicates no evidence for an association of environment and noise with nature relatedness. The point estimates indicate that nature relatedness increased slightly more in forests, compared with urban built environments with substantial statistical uncertainty. The confidence intervals include zero, indicating that the true association may be negligible or absent. Given the imprecision of the estimate, there is no evidence of an association. Furthermore, no evidence for an association between RQT and nature relatedness was found. Additionally, no evidence for an interaction between environment and noise on nature relatedness was shown. Further, little evidence was found for the effects of all the covariates.

S19 Table. Effects of model with RQT on nature relatedness

	Estimate	SE[1]	Lower CI[2]	Upper CI[2]	p
Effect of environment averaged across RQT	-0.044	0.047	-0.135	0.048	0.350
Effect of RQT averaged across environment conditions	-0.001	0.001	-0.002	0.001	0.283
Effect of RQT in urban built conditions	-0.001	0.002	-0.004	0.002	0.424
Effect of RQT in forest conditions	-0.001	0.001	-0.002	0.001	0.464
Age	-0.000	0.001	-0.002	0.002	0.949
Gender (male)[3]	0.054	0.041	-0.026	0.134	0.185
Gender (other)[3]	0.132	0.201	-0.264	0.527	0.512
Highest education high school, teacher training college[4]	0.090	0.070	-0.048	0.228	0.200
Highest education higher technical or vocational education, art school[4]	0.109	0.075	-0.039	0.256	0.148
Highest education University of Applied Sciences or University of Teacher Education[4]	0.050	0.058	-0.065	0.164	0.393
Highest education other	-0.462	0.255	-0.963	0.040	0.071
Frequency of greenspace visits 2–3 times a month5	-0.112	0.117	-0.342	0.118	0.340
Frequency of greenspace visits once a week[5]	-0.146	0.110	-0.363	0.071	0.186
Frequency of greenspace visits several times a week[5]	-0.154	0.108	-0.365	0.058	0.154
Frequency of greenspace visits daily[5]	-0.134	0.119	-0.369	0.101	0.264
Interaction environment: RQT	0.001	0.002	-0.003	0.004	0.685

[1]SE=standard error. [2]CI= confidence interval. [3]Referece for gender= female. [4]Reference for highest education= Primary/secondary modern/district school/lower grammar school & apprenticeship, vocational school, business school; trade school. [5]Reference for frequency of greenspace visits = fewer than 2–3 times a month.

S20 Table indicates substantial statistical uncertainty for an association of environment and noise with the Love and Care for Nature Scale. The point estimates suggest that love and care for nature decreased slightly more in forests, compared with urban built environments and increased slightly with increasing RQT with substantial statistical uncertainty. However, the confidence intervals include zero, indicating that there is no evidence for an association. Additionally, no evidence for an interaction between environment and RQT on love and care for nature was shown. Strong evidence was found that participants indicating higher technical or vocational education or art school as their highest education level reported a greater decrease in love and care for nature than those indicating primary or secondary school, modern or district school, lower grammar school, apprenticeship, vocational school, business or trade school as their highest level of education.

S10 Table. Effects of model with RQT on love and care for nature

	Estimate	SE[1]	Lower CI[2]	Upper CI[2]	p
Effect of environment averaged across RQT	0.015	0.069	-0.120	0.150	0.830
Effect of RQT averaged across environment conditions	-0.002	0.001	-0.004	0.000	0.101
Effect of RQT in urban built conditions	-0.003	0.002	-0.008	0.001	0.169
Effect of RQT in forest conditions	-0.001	0.001	-0.003	0.001	0.379
Age	0.002	0.002	-0.001	0.006	0.176
Gender (male)[3]	0.059	0.060	-0.060	0.177	0.330
Gender (other)[3]	-0.208	0.298	-0.793	0.378	0.486
Highest education high school, teacher training college[4]	0.179	0.104	-0.026	0.383	0.086
Highest education higher technical or vocational education, art school[4]	0.257	0.111	0.038	0.475	0.021
Highest education University of Applied Sciences or University of Teacher Education[4]	0.121	0.086	-0.048	0.290	0.161
Highest education other[4]	-0.395	0.377	-1.137	0.347	0.296
Frequency of greenspace visits 2–3 times a month[5]	0.021	0.173	-0.320	0.362	0.904
Frequency of greenspace visits once a week[5]	0.038	0.163	-0.283	0.359	0.817
Frequency of greenspace visits several times a week[5]	-0.039	0.159	-0.352	0.275	0.807
Frequency of greenspace visits daily[5]	-0.011	0.177	-0.358	0.337	0.952
Interaction environment: RQT	0.002	0.003	-0.003	0.007	0.390

[1]SE=standard error. [2]CI= confidence interval. [3]Referece for gender= female.
[4]Reference for highest education= Primary/secondary modern/district school/lower grammar school & apprenticeship, vocational school, business school; trade school.
[5]Reference for frequency of greenspace visits = fewer than 2–3 times a month.
