# Supplementary material for: Road traffic noise affects annoyance during urban built and forest walks, but not repetitive negative thinking or connectedness with non-human nature: A randomized controlled trial
Source: PLoS One. 2026 Mar 18;21(3):e0342906. doi: 10.1371/journal.pone.0342906 (PMC12998852; doi:10.1371/journal.pone.0342906)
Supplement: S6 File — (PDF) [file pone.0342906.s006.pdf]

S6. Coding scheme for “How thoughts change when you go for a walk”

Translated from the original German coding scheme

1. **Changes in thought flow and thought intensity**

   1.1 Reduced thought flow and thought intensity

   - Thoughts become fewer/ recede into the background/ become less intrusive
   - Less rumination
   - Thoughts slow down
   - Thoughts are replaced by observations

   1.2 Increased thought flow and thought intensity

   - More rumination
   - More intense
   - More intrusive thoughts
   - thoughts do not stop/ I think a lot/ thoughts become more/ deeper

2. **Change in thought valence**

   2.1 Thoughts become more positive

   - Thoughts become more positive/ I think about nice things
   - I think less about (personal) problems
   - Accepting/ less critical/ judgmental attitude towards thoughts
   - Interesting/ make me curious
   - More grateful

   2.2 become more negative

   - Become more negative
   - More problem-oriented
   - More distressing

3. **Thoughts and their flow**

   - More fleeting/ come and go at will/thoughts can spread/natural flow of thoughts/ more fluid/ I let them wander
   - Thoughts wander

4. **Change of perspective and relativisation of thoughts**

- Put things into perspective/ less important/ less serious
- Take a different perspective

5. Space for thoughts

- Can perceive thoughts better/ thoughts are heard
- Can think things through/ think things through to the end/ time for reflection
- Thoughts are not disturbed/distracted

6. Creativity and solution orientation

- Come up with different/new thoughts
- diverse/flexible/more free/liberated/more creative/more open
- More profound
- Find solutions/answers/solution orientation/more constructive

7. Gain distance

7.1. Mental distancing

- Can take a step back
- I can let go
- **I** can let go of thoughts more easily/detach myself from them

7.2 Emotional distancing

- Distraction from negative thoughts
- Less emotional

8. Inner peace and relaxation

- Thoughts calm down/become more relaxed
- Calm
- More peaceful
- Thoughts become less nervous/tense/erratic Erholung
- Meditative

9. Thought structure and clarity

- Thoughts become clearer and more organised/sorted
- More attentive/focused/concentrated

10. Change in thought content/focus

10.1. Internal focus

- Things that are currently on my mind come up

- I think about what I still have to gt done
- I hink about overarching topics/global issues

- 10.2 External focus
    - Thoughts focus on the environment
    - Thoughts are influenced by new/different surroundings

10.3 Present focus

- Thoughts focus on the moment

11. Unspecified categories

11.1 Unspecified change

- Thoughts change, not further specified in what way
- A little
- Thoughts do not change

11.2 No specific thoughts or relevance

- Did not think about anything
- Not relevant

11.3 Dependence on situation

- Depends on the day
