# Supplementary material for: Road traffic noise affects annoyance during urban built and forest walks, but not repetitive negative thinking or connectedness with non-human nature: A randomized controlled trial
Source: PLoS One. 2026 Mar 18;21(3):e0342906. doi: 10.1371/journal.pone.0342906 (PMC12998852; doi:10.1371/journal.pone.0342906)
Supplement: S7 File — (PDF) [file pone.0342906.s007.pdf]

S7. Coding scheme for “How does going for a walk help you to think through personal problems?”

Translated from the original German coding scheme

1. **Clear the mind and gain some distance**
   - Clear the mind /free your mind
   - Take your mind off things /think about other things
   - Gain perspective /distance (from everyday life, from problems)
   - Distraction (from problems, from everyday life)

2. **Promotion of clarity, order and reflection**
   - Gaining clarity
   - Organising thoughts /becoming more structured
   - Escaping the merry-go-round of thoughts /fewer thoughts spinning around
   - Able to think better /concentrate better
   - Time /space to think
   - Keep your mind flowing

3. **Gaining new perspectives and creative thinking**
   - Gaining a different perspective /a different point of view
   - More diverse /flexible /creative /new thoughts/ideas
   - Broader/ more open view
   - Get inspiration
   - Let thoughts run free /let them take their course

4. **Providing emotional relief and stress reduction**
   - Reduces stress
   - Fewer negative feelings /better coping with them
   - Worry less
   - Find peace
   - Calming
   - Becoming calmer/thinking more calmly/having peace and quiet
   - Serenity
   - Relaxation/recreation

5. **Experiencing nature and the surrounding area**
   - Fresh air/more oxygen
   - Nature gives me strength
   - I find nature comforting
   - I enjoy nature
   - Influence of flowers
   - Influence of sounds
   - Sun

6. **Self-awareness and inner balance**
   - Focus on myself /am more in tune with myself
   - Feel alive/free
   - Grounded
   - More self-confident
   - I am more in touch with my body
   - Perceive thoughts better
   - Make more conscious decisions
   - Greater awareness
   - Inner balance

7. **Put problems into perspective and find solutions**
   - I find solutions to problems/more solution-oriented
   - Approach problems differently
   - More rational/objective/less emotional
   - Problems are put into perspective/lose importance
   - See problems from a positive perspective
   - Helps with planning
   - Decide to do something about problems
   - More positive thinking

8. **Dependency on context and other influencing factors**
   - Depends on the environment
   - Depends on how long I walk
   - Depends on who I walk with
   - Depends on movement and walking speed
   - Discuss problems with colleagues

- Other factors (variety, other)

9. No or limited effect

- No effect
- Helps somewhat/sometimes
