# Supplementary material for: Road traffic noise affects annoyance during urban built and forest walks, but not repetitive negative thinking or connectedness with non-human nature: A randomized controlled trial
Source: PLoS One. 2026 Mar 18;21(3):e0342906. doi: 10.1371/journal.pone.0342906 (PMC12998852; doi:10.1371/journal.pone.0342906)
Supplement: S8 File — (PDF) [file pone.0342906.s008.pdf]

Gesuch an die Ethikkommission der ETH Zürich

Inhalt

A. Allgemeine Informationen 2
B. Wissenschaftliche Informationen 3
1. Abstract 3
2. Projekt 3
3. Ethische Erwägungen 10
4. Literatur 13
C. Anhang 15

A. Allgemeine Informationen

Projekttitel:

Restorative potential of green spaces in noise-polluted environments RESTORE<br>WP2 Field experiments

Projektleitung (PI):

Name	Titel	Gruppe / Professur / Institut	Hochschule
Silvia Tobias (PI)	Dr.	Landschaftsdynamik	WSL
Jean-Marc Wunderli (Co-PI)	Dr.	Akustik / Lärmbekämpfung	Empa
Nicole Bauer (Co-PI)	Dr.	Wirtschafts- und Sozialwissenschaften	WSL
Beat Schäffer (Co-PI)	Dr.	Akustik / Lärmbekämpfung	Empa

Beteiligte Mitarbeitende:

Name	Titel	Gruppe / Professur / Institut	Hochschule
Marcel Hunziker (Res.Partner)	Dr.	Wirtschafts- und Sozialwissenschaften	WSL
Christian Ginzler (Res.Partner)	DIng.	Landschaftsdynamik	WSL
Reto Pieren (Res.Partner)	Dr.	Akustik / Lärmbekämpfung	Empa
Natalia Kolecka (Wiss. MA)	Dr.	Landschaftsdynamik	WSL
María Garcia Martín (Postdoc)	Dr.	Landschaftsdynamik	WSL
Fotis Georgiou (Postdoc)	Dr.	Akustik / Lärmbekämpfung	Empa
Julia Schaupp (Dokotorandin)	MSc	Wirtschafts- und Sozialwissenschaften	WSL
Javier Dopico (Doktorand)	MSc	Akustik / Lärmbekämpfung	Empa

Finanzierungsquelle(n): SNF Sinergia grant, CRSII5_193847/1

Anzahl Teilnehmende:
Minimum: 270
Maximum: 300

Studiendauer:
Beginn: 1.11.2020
Ende: 31.10.2024

Projektart:
☒ Forschungsprojekt
☒ Bachelor-/Master-/Doktorarbeit

B. Wissenschaftliche Informationen

Die Studie beinhaltet:

☒ Online-Erhebung ☐ Smartphonebasierte Datenerhebung ☒ Interviews / Gruppendiskussionen

☒ Physische Aktivitäten ☐ Klinische Versuche im Ausland

1. Abstract

Das vorliegende Gesuch an die Ethikkommission bezieht sich auf das Arbeitspaket 2 des Projekts «Restorative Potential of green spaces in noise-polluted environments». Das Gesamtprojekt umfasst vier Arbeitspakete (WP1-WP4) und untersucht das Potenzial von Grünräumen für die Erholung von Stress und die Verbesserung von Wohlbefinden sowie Lärm als einschränkenden Faktor für Erholung. Ein Gesuch zur Beurteilung von WP3 und WP4 wurde unter der Nummer EK 2021-N-116 bereits im August durch die Ethikkommission der ETHZ genehmigt.

Die geplante Studie in WP2 untersucht, ob Personen sich in unterschiedlich lärmbelasteten Umgebungen in der Schweiz von Stress erholen können. Die Studie umfasst Feldexperimente, um die Effekte von 30-minütigen Spaziergängen in naturnahen (Wald) versus urbanen Umgebungen (Strassen) mit unterschiedlichem Verkehrslärmpegel auf Stress, Wohlbefinden, Erholung und Aufmerksamkeit zu vergleichen. Neben Verkehrslärm als beeinträchtigenden Faktor beschäftigt sich die Studie mit Ansätzen zur Steigerung der positiven Wirkung naturbasierter Interventionen. Frühere Studien zeigen positive Effekte von achtsamkeitsbasierten Interventionen auf Stress und Wohlbefinden. Der Natur mit einer Haltung der Achtsamkeit zu begegnen, könnte so zu einer stärkeren Verbesserung des subjektiven Wohlbefindens führen. Entsprechend wird in der aktuellen Studie untersucht, ob eine Achtsamkeitsintervention den Effekt von Spazierengehen in der Natur auf Stress und Wohlbefinden erhöht.

Bei den Feldexperimenten finden standardisierte Spaziergänge in naturnahen und urbanen Umgebungen statt, bei denen Speichel-Cortisol und Hautleitfähigkeit als physiologische Indikatoren für Stress erhoben werden. Zudem werden vor und nach dem Spaziergang ein Aufmerksamkeitstest und Fragebögen zu Wohlbefinden, Erholung, Lärmempfindlichkeit, Lärmbelästigung sowie Naturverbundenheit eingesetzt. Die Achtsamkeitsintervention wird in standardisierter Art und Weise umgesetzt.

Das Gesamtprojekt wird die Zusammenhänge zwischen Lärmbelastung und Stress in Abhängigkeit von Grünflächen aufzeigen. Es wird feststellen, wie gut die Schweizer Bevölkerung mit erholungswirksamen Grünräumen versorgt ist und wo Verbesserungsbedarf besteht. Schliesslich wird es Hinweise zur Umsetzung der schweizerischen Lärmschutzgesetzgebung und des revidierten Raumplanungsgesetzes liefern. Die Ergebnisse der Studie in WP2 werden im Speziellen Aufschluss über die restaurative Wirkung verschiedener Umwelten und den Einfluss unterschiedlicher Lärmpegel in diesen Settings geben. Darüber hinaus wird die Studie Erkenntnisse dazu liefern, inwiefern eine Veränderung der Haltung, mit der Menschen der Natur begegnen, die positiven Effekte von Naturkontakt erhöhen kann.

2. Projekt

2.1 Zielsetzung

Stress ist ein weit verbreitetes und belastendes Phänomen in unserer Gesellschaft. Angesichts dessen bedarf es Forschung zu Stressreduktion sowie zu Ansätzen, die auf die Prävention

psychischer Belastungen und die Erhöhung von Wohlbefinden ausgerichtet sind. Entsprechend weist eine zunehmende Anzahl wissenschaftlicher Studien darauf hin, dass Kontakt mit der Natur Stress reduzieren und das Wohlbefinden steigern kann. Naturexposition und naturbasierte Interventionen sind demnach vielversprechende Ansätze. Gleichwohl leben heute immer mehr Menschen in urbanen Regionen, in denen, verglichen mit dem ländlichen Raum, mehr Umweltstressoren wie Lärm und weniger direkter Zugang zu Natur zu finden sind. Obwohl verschiedene frühere Studien die positiven Gesundheitseffekte von Kontakt mit natürlichen versus urbanen Umwelten vergleichen, mangelt es bisher an Studien, die die spezifischen Auswirkungen von Lärm in diesem Zusammenhang erforschen.
Die geplante Studie in WP2 umfasst die Untersuchung der Effekte von 30-minütigen Spaziergängen in naturnahen versus urbanen Umgebungen mit unterschiedlichem Verkehrslärmpegel auf Stress, Wohlbefinden, Erholung und Aufmerksamkeit. Darüber hinaus wird in der Studie untersucht, ob eine Achtsamkeitsintervention den Effekt von Spazierengehen in der Natur auf Stress und Wohlbefinden verändert. Bisher mangelt es an Feldstudien zu den Effekten von Lärm als einschränkendem Faktor für Erholung. Ziel der Studie ist es, anhand physiologischer Parameter, Fragebogen-Daten sowie mit Hilfe eines Aufmerksamkeitstests die Effekte von Spaziergängen in unterschiedlich lärmbelasteten Umgebungen zu untersuchen, um Einsichten in die restaurative Wirkung verschiedener Umwelten (urban vs. naturnah) und den Einfluss unterschiedlicher Lärmpegel in diesen Settings zu gewinnen. Darüber hinaus soll mit der Implementierung der Achtsamkeitsintervention untersucht werden, inwiefern eine Veränderung der Haltung, mit der Menschen der Natur begegnen, die positiven Effekte von Naturkontakt erhöhen kann. Auf diese Weise wird in WP2 die Wirkung von Achtsamkeitsintervention in naturnahen Umgebungen untersucht.

2.2 Zeitliche Planung

Das vorliegende Gesuch wird unabhängig von den anderen Arbeitspaketen eingereicht, da im Voraus des Gesuchs eine Achtsamkeitsintervention entwickelt wurde. Zudem reichen vorhandene Lärmdaten nicht aus, um geeignete Settings für die Feldexperimente auszusuchen. Daher müssen vor-Ort Messungen des Lärmpegels in potenziellen Settings durchgeführt werden, um zu prüfen, ob diese im Hinblick auf den gemessenen Lärmpegel stark genug differenzieren, was mehr Zeit in Anspruch nimmt. Die geplanten Pretests sollen neben der Testung des Fragebogens darüber aufklären, welche Settings passend sind.

Zeit	WP2
Nov 20	Projektstart
Nov 20 – Jan 21	Konzeptionelle Planung des Arbeitspakets<br>Konzeption der Schnittstellen zwischen den WPs
Feb – Aug 21	Konzeption und Entwurf des Fragebogens<br>Entwicklung der Achtsamkeitsintervention<br>Vorauswahl der urbanen Testgebiete
Aug – Dez 21	Prüfung der Vorauswahl der urbanen Testgebiete: Audioaufnahmen und Analyse potenzieller Testsettings zur Prüfung der Eignung aufgrund auditiver Merkmale.<br>Auswahl korrespondierender naturnaher Settings<br>Explorative Befragung zu Effekten von Spazierengehen in unterschiedlich lärmbelasteten Umgebungen.

	Weiterentwicklung der Achtsamkeitsintervention<br>Ethikantrag
Dez- Feb 22	Qualitative Befragung zu Effekten von Spazierengehen in unterschiedlich lärmbelasteten Umgebungen.<br>Pretests<br>Auswertung der Pretests<br>Einarbeitung der Ergebnisse des Pretests in die Planung und Konzeption
Mär – Apr 22	Vorbereitung Feldexperimente 1
Mai – Sept 22	Feldexperimente 1
Okt 22 – Feb 23	Datenauswertung
Mär - Apr 23	Vorbereitung Feldexperimente 2
Mai – Sept 23	Feldexperimente 2
Okt 23- Okt. 24	Datenauswertung<br>Publikationen, Abschluss Dissertation
Okt 24	Projektende

2.3 Methoden

In WP2 wird zum einen eine explorative Befragung durchgeführt (a) und zum anderen eine Feldstudie (b).

Ad a: Bei der explorativen Befragung werden Teilnehmende gebeten eine halbe Stunde an einem Ort ihrer Wahl in einer naturnahen (Bedingung 1) oder urbanen (Bedingung 2) Umgebung spazieren zu gehen. Für den Einladungsbrief zur Befragung siehe Anhang C1. Im Anschluss an den Spaziergang beantworten die Teilnehmenden offene Fragen zu ihren Erfahrungen beim Spazierengehen (siehe Fragebögen im Anhang C2 und C3). Teilnehmende müssen dabei nicht angeben, wo genau sie spazieren gegangen sind. Die Antworten der explorativen Befragung werden zum einen Informationen darüber geben, ob weitere wichtige Faktoren für die Erholung beim Spazieren in unterschiedlichen Umgebungen eine Rolle spielen, zum anderen werden die Antworten aus der Befragung die Erkenntnisse aus der Feldstudie in Form von freieren Berichten der Proband*innen ergänzen.

Ad b: Bei der Feldstudie geht ein Teil der Proband*innen in einem naturnahen Setting spazieren, ein anderer Teil der Proband*innen spaziert in einem urbanen Setting. Bei der Feldstudie handelt es sich um eine randomisierte Interventionsstudie mit Feldexperimenten, basierend auf einem Pretest-Posttest Design. Das Studiendesign ist ein between-subjects Design, jede/r Proband*in durchläuft nur eine Bedingung im Experiment. Teilnehmende werden randomisiert einer der Gruppen zugewiesen. Eine Gruppe wird in einem urbanen Setting mit viel Verkehrslärm spazieren gehen, eine zweite Gruppe in einem urbanen Setting mit wenig Verkehrslärm. Eine dritte Gruppe wird in einem naturnahen Setting mit viel Verkehrslärm und eine Vierte in einem naturnahen Setting mit wenig Verkehrslärm spazieren. Eine fünfte Gruppe wird einen Spaziergang in einem naturnahen Setting mit wenig Verkehrslärm mit einer Achtsamkeitsintervention spazieren gehen (siehe Abb. 1). Die Achtsamkeitsintervention umfasst die Fokussierung auf unterschiedliche Sinneseindrücke (z.B. was sehen die Proband*innen aktuell? Was hören sie?) (siehe Anhang C10).

Abb. 1. Design der Feldexperimente

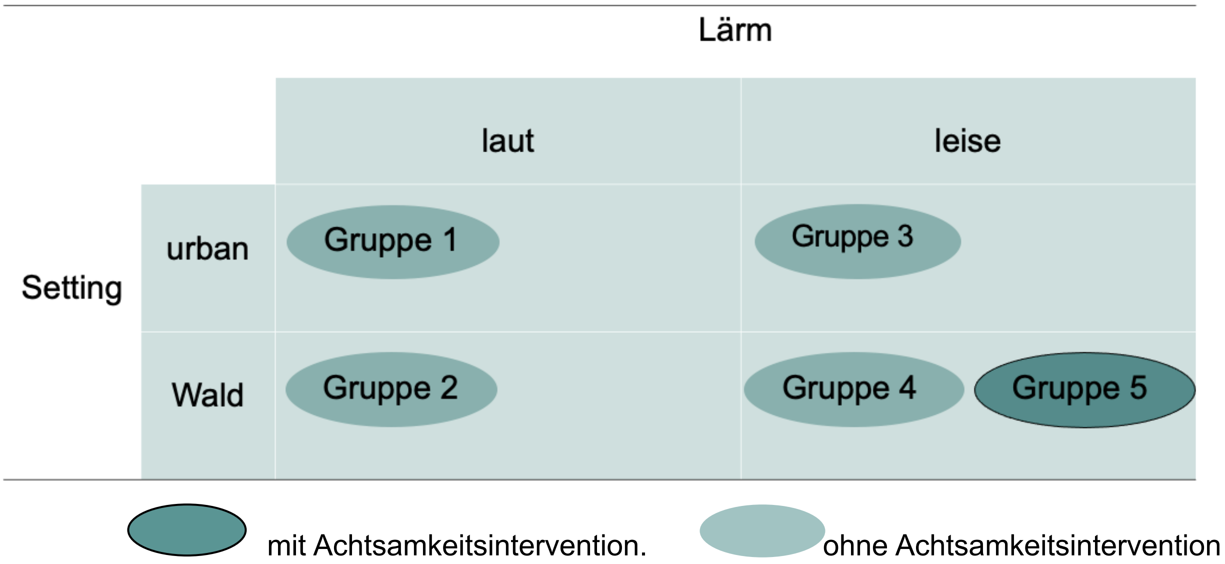

Die Testsettings werden anhand von GIS-Analysen, basierend auf modellierten Lärmdaten und Daten zu Vegetationshöhen und dem Normalised Difference Vegetation Index (NDVI) sowie der Inspektion von Verkehrszählungsdaten der Stadt Zürich zum motorisierten Individualverkehr mit Zählstellen an unterschiedlichen Standorten in Zürich vorausgesucht. Mit Hilfe der modellierten Lärmdaten werden drei Klassen der Lärmbelastung gebildet: (1) dB< 35, (2) dB=35-44, (3) dB ≥ 45. Anhand von Vegetationshöhen und dem NDVI werden drei Klassen an Vegetationsstruktur bestimmt (kaum Grünflächen; Grünflächen mit niedriger Vegetation, z.B. Rasen; Grünflächen mit hoher Vegetation, z.B. Baumbestände). Basierend auf den Kombinationen der Lärmklassen und der Vegetationsklassen sowie den Verkehrszählungsdaten der Stadt Zürich wird eine Vorauswahl der Testsettings getroffen. Die potenziellen Settings werden mit Hilfe von Sattelitenbildern und schließlich durch Vor-Ort-Begehungen weiter erkundet. Daraufhin wird die Vorauswahl potenzieller Settings weiter reduziert. Im Anschluss finden vor Ort Messungen des Lärmpegels in den potenziellen Testsettings statt um zu prüfen, ob diese im Hinblick auf den gemessenen Lärmpegel stark genug differenzieren. Schließlich werden geeignete Settings ausgesucht.

Im Feldexperiment selbst werden die Proband*innen in Gruppen von 2-6 Personen im entsprechenden Setting auf einer standardisierten Strecke spazieren. Feldexperimente zu Spazierengehen in naturnahen Umgebungen finden meist in Gruppen statt (Vgl. beispielsweise Tyvräinen et al., 2014). Der Spaziergang wird ca. 30 Min. dauern, während des Spaziergangs werden die Proband*innen von einer Testleitung begleitet. In den Gruppen mit Achtsamkeitsintervention werden die Proband*innen während des Spaziergangs eine geleitete Achtsamkeitsübung machen (siehe Anhang C10). Proband*innen werden gebeten, sich während des Spaziergangs nicht zu unterhalten. Spaziergänge werden nur bei stabilen Wetterbedingungen stattfinden.

Daten werden anhand physiologischer Messung (Speichelkortisol, Hautleitwert), online Fragebögen und einem Aufmerksamkeitstest erfasst (siehe Abb. 2). Die Daten werden vor (t1) und nach (t2) dem Spaziergang erhoben. Im Anschluss an das Experiment vor Ort werden die Proband*innen gebeten, in den folgenden 10 Tagen noch drei Mal eine halbe Studie allein, an einem Ort ihrer Wahl in der Natur oder in einem urbanen Setting sowie mit oder ohne Achtsamkeitsintervention (je nach zugeordneter Bedingung) spazieren zu gehen. 10 Tage nach der Teilnahme am Feldexperiment bekommen die Proband*innen erneut einen Link mit der Bitte,

einen online Fragebogen auszufüllen zugeschickt (siehe Abb. 2). Proband*innen in den Bedingungen mit Achtsamkeitsintervention werden für die individuellen Spaziergänge zw. T2 und T3 nach dem Spaziergang und der Datenerhebung (t2) eine kurze schriftliche Anleitung mit der Achtsamkeitsübung bekommen. Zusätzlich wird diese digital an die Teilnehmenden weitergegeben (siehe Kapitel 3 zu Datenschutz).

Die Datenerhebung ist in zwei Wellen, von April 2022– Oktober 2022 sowie von April 2023 – Oktober 2023 geplant, um Verzerrungen der Resultate aufgrund spezieller Bedingungen in einzelnen Jahren zu vermeiden.

Abb. 2. Studienablauf

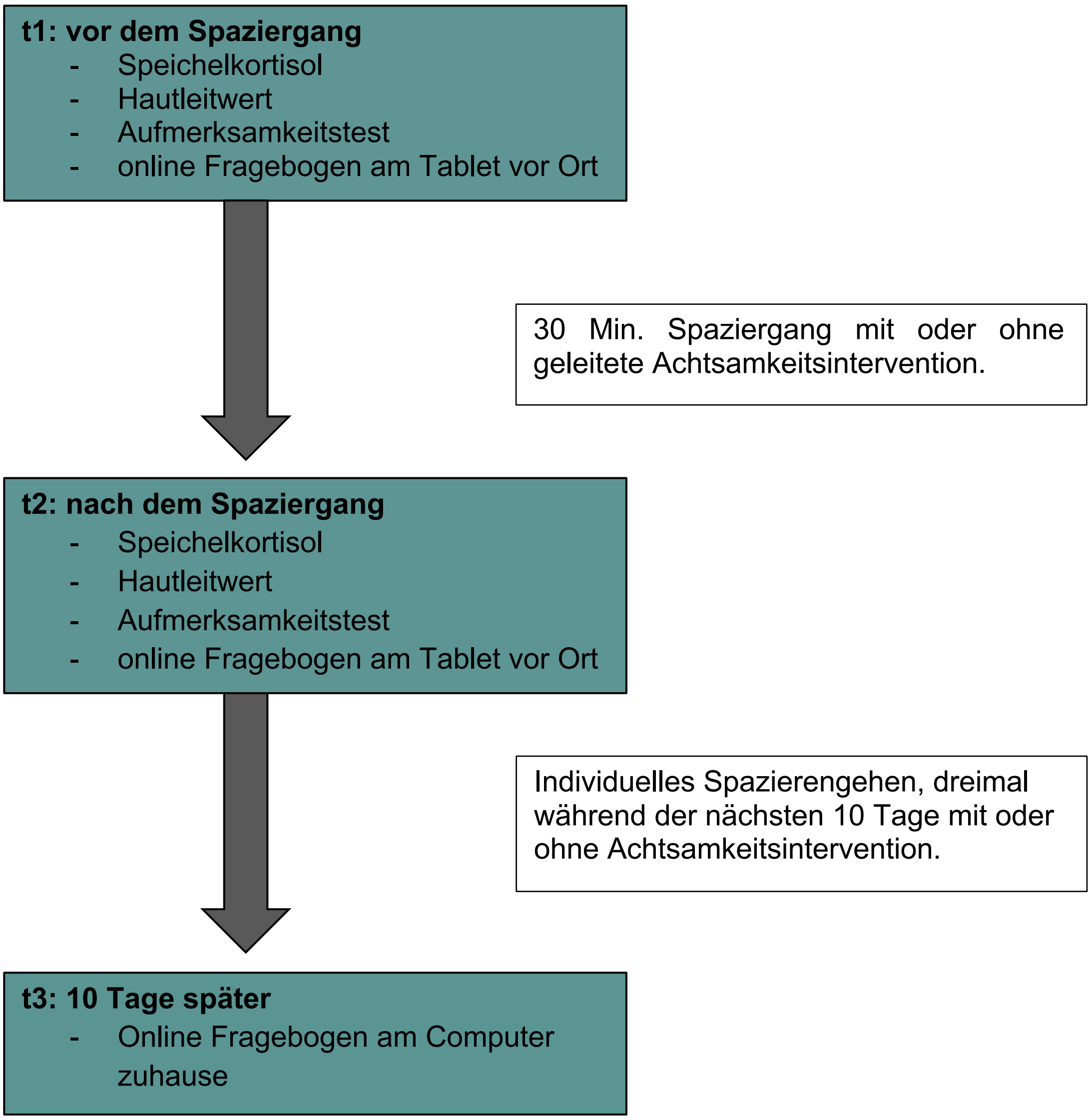

Im Zuge der Untersuchung des Spaziergangs vor Ort (t1 und t2) werden die Teilnehmenden zunächst begrüßt und über den Ablauf und Zweck der Studie informiert (siehe Teilnehmendeninformation im Anhang C5). Im Anschluss werden Speichelkortisol und Hautleitwert als physiologische Indikatoren für Stress und körperliche Anspannung erhoben, um zu erfassen, inwiefern sich die Teilnehmenden im Zuge des Spaziergangs erholen. Speichelkortisol wird zweimal erhoben, zu t1 und zu t2. Teilnehmende kauen dafür zu t1 und t2 jeweils ca. 1 Min. auf einem kleinen Wattebausch (Sarstedt Cortisol Salivette; Sarstedt AG & Co, Germany). Nachdem die Teilnehmenden zu t1 die Speichelkortisolprobe abgegeben haben, findet die physiologische Messung mit Hilfe eines Geräts zur Erfassung des Hautleitwerts (z.B. Shimmer3 GSR+ sensor, Simmer sensing, Dublin, Irland) statt. Entsprechende Elektroden zur

Erfassung des Hautleitwerts werden an der nicht-dominanten Hand der Teilnehmenden angeklebt und während des 30-minütigen Spaziergangs vor Ort getragen. Nach dem Spaziergang werden diese wieder entfernt. Nach der Applikation des Geräts zur Erfassung des Hautleitwerts wird den Teilnehmenden ein Aufmerksamkeitstest vorgelegt (Necker Cube Pattern Control Test; De Young, 2016). Für die genaue Instruktion zum Test siehe Anhang C6. Diesen beantworten die Teilnehmende in Papierform. Dabei wird die Anzahl der Perspektivwechsel mit Hilfe eines Handzählgeräts durch die Teilnehmenden erfasst. Im Anschluss daran beantworten die Teilnehmenden einen online Fragebogen.

Die Fragebogen-Daten werden voraussichtlich mit einer Software des Dienstleisters Socscisurvey (Leiner, 2014) erhoben. In den Fragebögen werden folgende Themenbereiche adressiert: (a) (j) State-Achtsamkeit, (b) Trait-Achtsamkeit, (c) Wohlbefinden, (d) Naturverbundenheit, (e) chronischer Stress, (f) Lebenszufriedenheit, (g) Rumination, (h) Gedankenfokus, (i) soziodemografische Daten, (j) auditiver Eindruck während des Spaziergangs, (k) Belästigung durch Lärm während des Spaziergangs, (l) Erholung, (m) Voerfahrung mit Achtsamkeit, (n) Vegetation, in der Umgebung des Spaziergangs.

In den Fragebögen werden wo möglich standardisierte Skalen verwendet. Im Folgenden werden die untersuchten Konzepte und die entsprechenden zur Messung verwendeten standardisierten Skalen erläutert.

Achtsamkeit. Nach Creswell (2017) tauchen zwei Aspekte in einer Vielzahl an Definitionen von Achtsamkeit auf: Erstens bezieht Achtsamkeit sich auf die Fokussierung der Aufmerksamkeit und des Bewusstseins auf den gegenwärtigen Moment (Creswell, 2017). Dies umfasst das aufmerksame Wahrnehmen von Körperempfindungen, emotionalen Reaktionen, Kognitionen und Sinneserfahrungen. Der zweite Aspekt bezieht sich auf eine durch Offenheit und Akzeptanz geprägte Haltung gegenüber augenblicklichen Erfahrungen. Darunter wird die Fähigkeit verstanden, gegenwärtige Empfindungen und Gedanken auf eine nicht-bewertende Art und Weise wahrzunehmen, ohne sich mit ihnen zu identifizieren und die Tendenz, alle mentalen Inhalte und Wahrnehmungen automatisch zu kategorisieren anzuhalten (Bishop, 2004). In WP2 wird Achtsamkeit sowohl als State (Zustand), als auch als Trait (Persönlichkeitseigenschaft) erfasst. *States* sind vorübergehende Zustände, also aktuelle Stimmungen oder das momentane Befinden. Die State-Achtsamkeit wird in WP2 mit der State Mindfulness Scale (Tanay & Bernstein, 2013) erfasst. Diese quantifiziert das Ausmaß der momentanen Aufmerksamkeit und der bewussten Wahrnehmung im Hinblick auf Körperempfindungen und mentale Ereignisse in einem spezifizierten Zeitraum. Die State Mindfulness Scale wurde entwickelt, um das Ausmaß der Achtsamkeit direkt nach einer Achtsamkeitsintervention zu messen. *Traits* sind im Vergleich zu *States* zeitlich stabilere Eigenschaften einer Person. Im Hinblick auf Achtsamkeit wird angenommen, dass über einen Anstieg der State-Achtsamkeit durch wiederholtes Praktizieren von Achtsamkeit die Trait-Achtsamkeit von Praktizierenden steigt. Die Trait-Achtsamkeit wird in WP2 mit der Kurzform des Kentucky Inventory of Mindfulness Skills (Höfling et al., 2011.) erfasst.

Wohlbefinden. Das momentane Wohlbefinden im Sinne der emotionalen Befindlichkeit wird mit der deutschen Version der Positive and Negative Affect Schedule (PANAS, Watson et al., 1988.) erfasst. Diese besteht aus 20 Adjektiven, die unterschiedliche Empfindungen und Gefühle auf den Dimensionen Positiver und negativer Affekt beschreiben.

Naturverbundenheit. Zylstra (2014) beschreibt Naturverbundenheit als „stable state of consciousness comprising symbiotic cognitive, affective, and experiential traits that reflect, through consistent attitudes and behaviours, a sustained awareness of the interrelatedness between one's self and the rest of nature". Bisherige Studien deuten auf einen positiven Effekt von Naturaufenthalt auf die subjektive Naturverbundenheit hin (Mayer & Frantz, 2009). In WP2

wird die emotionale Komponente der Naturverbundenheit mit Hilfe der Love and are for nature scale (Perkins, 2010) erfasst, die kognitive Komponente der Naturverbundenheit wird mit der Kurzform der Nature Relatedness Scale (Nisbet, Zelenski & Murphy, 2009) erhoben.

Chronischer Stress. Frühere Studien haben einen Stress reduzierenden Effekt von Aufenthalt in der Natur bei Personen mit geringem chronischem Stress gezeigt, aber einen umgekehrten Zusammenhang für Menschen mit hohem Level an chronischem Stress (Hofmann et al., 2018). Aus diesem Grund wird in WP2 das chronische Stress Level als Moderator für den Effekt von Naturaufenthalt auf momentanen Stress erfasst. Chronischer Stress wird mit der Screening Skala für chronischen Stress (SSCS) des Trierer Inventars für chronischen Stress erhoben (TICS-SSCS; Schulz et al., 2004), ein weitverbreitetes Stressinventar.

Lebenszufriedenheit. Die Lebenszufriedenheit wird mit der Kurzskala Lebenszufriedenheit-1 (Beierlein et al., 2014.) erfasst.

Rumination. Nolen-Hoeksema und Kolleg*innen (2008) definieren Rumination als “mode of responding to distress that involves repetitively and passively focusing on symptoms of distress and on the possible causes and consequences of these symptoms”. Rumination resultiert nicht in aktivem Problemlösen, sondern in wiederholtem und wiederkehrendem Grübeln über Gefühle und Probleme. Nach Ehring et al. (2011) sind die Hauptkomponenten von repetitivem negativem Denken die hohe Wiederholungshäufigkeit, die Aufdringlichkeit der Gedanken und die Schwierigkeit, sich von negativen Gedanken zu lösen. In WP2 wird das repetitive negative Denken im Sinne von Rumination mit dem Perseverative Thinking Questionnaire (Ehring, et al., 2011.) erhoben.

Gedankenfokus. WP2 untersucht unter anderem, ob die Proband*innen im Zuge eines 30-minütigen Spaziergangs eine Veränderung im Gedankenfokus berichten. Dieser wird mit der Situaltional Self-Awareness Scale (Govern, & Marsch, 2001) erhoben. Diese unterscheidet zwischen private awareness, public awareness und awareness of immediate environment (Govern & Marsch, 2001).

Erholung. Nach Person und Craig (2014) wird in WP2 zwischen der wahrgenommenen restaurativen Qualität der Umgebung und der tatsächlichen Erholung nach dem Naturaufenthalt unterschieden. Die wahrgenommene restaurative Qualität der Umgebung wird mit der Perceived Restorativeness Scale (PRS-11; Pasini et al., 2014) erfasst. Die Erholung nach dem Spaziergang wird mit der Restoration Outcome Scale (ROS; Korpela et al., 2008) erhoben.

Auditiver Eindruck. Der auditive Eindruck wird mit Hilfe der Soundscape Skala nach der Empfehlung der ISO/TS erhoben (ISO/TS 12913-2:2018, 2018).

Die restlichen Fragen werden ohne standardisierte Skalen erfasst. Im Fragebogen zu T3 werden die Teilnehmenden außerdem gebeten, zwischen verschiedenen Fotos zu vergleichen und anzugeben, welches im Hinblick auf das Ausmaß der Vegetation am ehesten der Umgebung ähnelt, in der der/ die Proband*in in den letzten 10 Tagen spazieren war. Die Fotos werden von Expert*innen hinsichtlich der Ausprägung des NDVI geratet.

Der vollständige Fragebogen ist dem Gesuch im Anhang C7-C9 beigelegt. Dort befinden sich Verweise darauf, wo es sich um standardisierte Skalen handelt.

Die Datenerhebung ist in zwei Wellen, von April 2022– Oktober 2022 sowie von April 2023 – Oktober 2023 geplant, um Verzerrungen der Resultate aufgrund spezieller Bedingungen in einzelnen Jahren zu vermeiden.

2.4 Projektpartner

Finanzierung: SNF Sinergia grant CRSII5_193847/1
Am Projekt beteiligte Institutionen: Eidgenössische Forschungsanstalt WSL, Eidgenössisch Materialprüf- und Forschungsanstalt Empa

3. Ethische Erwägungen

3.1 Teilnehmende

Die Rekrutierung der Proband*innen für die Feldstudie erfolgt möglichst breit gestreut, um ein möglichst breites Bild der Grundgesamtheit der Population (bspw. hinsichtlich Bildungsstand, Einkommen, Alter usw.) abzudecken. Die Rekrutierung erfolgt über örtliche Hochschulen (Universität Zürich, ETH Zürich und Fachhochschulen im Raum Zürich (ZHAW, PHZH, ZHdK)), über weitere Institutionen wie EMPA, Eawag, die Kantonale Verwaltung, Gewerkschaften, Volkshochschulen, über Verbände (bspw. Züricher Senioren und Rentnerverband), Vereine und Firmen. Zudem wird über das Einwohnermeldeamt der Stadt Zürich eine Stichprobe mit Personendaten aus dem Einwohnerregister (Name, Adresse) gezogen und per Post kontaktiert. In diesem Fall wird eine Autorisierungsanfrage an die Stadt Zürich gestellt. Die Daten werden nur bezogen, wenn der zuständige Rechtsdienst das Personenmeldeamt zur Herausgabe der Adressen autorisiert. Die potenziellen Proband*innen werden in diesem Fall entsprechend des Rekrutierungsschreibens im Anhang C4 rekrutiert.

Teilnehmende müssen mindestens 18 Jahre alt sein, körperlich dazu in der Lage sein, eine halbe Stunde in moderatem Tempo spazieren zu gehen und dürfen keine diagnostizierten Hörprobleme haben. Aufgrund der Cortisol-Erhebungen dürfen die Teilnehmenden darüber hinaus kein Cortison einnehmen, es darf kein Cushing Syndrom vorliegen und Personen mit BMI über 35 können nicht an der Studie teilnehmen, da dies die Cortisolwerte verfälschen kann. Teilnehmende dürfen 24 Stunden vor der Studienteilnahme keine Drogen konsumieren und zwei Stunden vor der Studienteilnahme keinen Kaffee oder Alkohol trinken und keinen anstrengenden Sport betreiben.

Es werden 45 Teilnehmende pro Bedingung angestrebt, mind. 270 insgesamt. Proband*innen bekommen für die Teilnahme an der Studie eine Aufwandsentschädigung. Nach Möglichkeit bekommen die Versuchspersonen außerdem Versuchspersonen-Stunden angerechnet, die sie innerhalb ihres Studiums obligatorisch erbringen müssen (für Psychologiestudierende der Fall).

Für die explorative Studie werden die Teilnehmenden über verschiedene Verteiler per Mail rekrutiert (z.B. Universitätsverteiler der Universität Basel, Verteiler der Eidg. Anstalt für Wald, Schnee und Landschaft, Initiative Psychologie im Umweltschutz, Fachgruppe Psychologie Universität Basel). Nach Möglichkeit bekommen die Proband*innen für die Teilnahme an der Studie ebenfalls Versuchspersonen-Stunden angerechnet, die sie innerhalb ihres Studiums obligatorisch erbringen müssen.

Alle Teilnehmenden bekommen eine allgemeine Teilnehmendeninformation für die Feldstudie und unterschrieben eine Einwilligungserklärung zur Teilnahme an der Studie (siehe C5).

3.2 Risiken und Datenschutz

Gesundheitsschutz
Die Studie ist mit keinerlei Gesundheitsrisiken für die Befragten verbunden. Die Cortisolmessung und die Erfassung des Hautleitwerts sind nicht-invasive Verfahren, die schmerzfrei erhoben werden. Der Klebestreifen, mit dem die Elektroden befestigt sind, kann nach dem Spaziergang schmerzfrei entfernt werden. Es werden keine gesellschaftlichen oder politischen Risiken erwartet, da die Studie die Effekte von Erholung bei Spaziergängen in unterschiedlich

lärmbelasteten Umgebungen zum Thema hat. Die Achtsamkeitsintervention beruht darauf, dass die Teilnehmenden momentane sensorische und emotionale Erfahrungen wahrnehmen. Achtsamkeitsinterventionen berufen dabei auf dem Prinzip, dass nicht nur angenehme, sondern auch unangenehme Erfahrungen wahrgenommen werden sollen. Theoretisch besteht daher die Möglichkeit, dass sich Teilnehmende durch die Achtsamkeitsintervention negativer Gefühle bewusster werden. Die Forschung zu Achtsamkeitsinterventionen zeigt jedoch einen Anstieg des Wohlbefindens und eine Reduktion von Stress durch das Praktizieren von Achtsamkeit sowie positive Effekte auf gesundheitliche Outcomevariablen (Nyklíček & Kuijpers, 2008; Shapiro et al., 2008; Brown & Ryan, 2003; Gotnik et al., 2015; Grossman et al., 2010; Hanley, Warner & Garland, 2015; Parsons et al., 2017), weshalb dies nicht zu erwarten ist.

Datenschutz

Vor Ort erhalten die Teilnehmenden einen persönlichen Identifikations-Code (PID), der zufällig generiert und zugeteilt wird (https://www.voucherify.io/generator). Das Dokument, das die Namen der Individuen mit ihren PID verbindet, wird verschlüsselt und nur die jeweiligen WP-Mitarbeitenden erhalten Zugang zu diesem Schlüssel. Teilnehmede tragen ihre PID in die online Fragebögen ein, die Daten im online Fragebogen werden also pseudonomisiert erhoben.

Ein Teil der Teilnehmenden wird der Bedingung mit Achtsamkeitsintervention zugewiesen werden. Vor und im Anschluss an den geleiteten Spaziergang vor Ort mit Achtsamkeitsintervention füllen die Teilnehmenden den online Fragebogen aus. Nach dem Spaziergang bekommen die Teilnehmenden ein Papierdokument mit einer Anleitung für die Achtsamkeitsübung, die sie während mind. drei halbstündigen individuellen Spaziergängen in den nächsten 10 Tagen durchführen sollen. Zusätzlich werden die Teilnehmenden gebeten am Ende des Fragebogens nach dem geleiteten Spaziergang mit Achtsamkeitsintervention ihre Emailadresse anzugeben. Den Teilnehmenden in der Achtsamkeitsbedingung wird die Achtsamkeitsübung noch einmal per Mail zugeschickt. Zudem wird allen Teilnehmenden der Link zum Fragebogen zu t3 (10 Tage nach dem Spaziergang vor Ort) zugesandt. Die Emailadresse wird getrennt von den Angaben im Fragebogen gespeichert, die Daten im Fragebogen bleiben dabei weiterhin anonym. Die Emailadressen werden nicht an Dritte weitergeben. Sie sind lediglich für die Dauer der Erhebung und nur für die projektdurchführende Person zugänglich. Nach Beendigung der Erhebung bzw. spätestens bei Projektende werden die Emailadressen der Befragten gelöscht. Da mit der Emailadresse personenbezogene Daten erhoben werden, wird mit dem Fragebogenportal ein Datenverarbeitungsvertrag im Auftrag (AVV) nach EU-Datenschutzgrundverordnung (DSGVO) vereinbart, der für die getrennte Erhebung von Kontaktdaten gilt (siehe Mustervertrag in Anhang C11).

Anonymisierungsstrategie

Mit einem Zufallsgenerator (https://www.voucherify.io/generator) werden Codes generiert, die den Proband*innen zugeordnet werden. Dabei handelt es sich um vierstellige Kombinationen von Zahlen und Buchstaben der Form ##-## (# steht für jeweils eine Zahl oder einen Buchstaben). Die Unterteilung durch Bindestriche soll es den Teilnehmenden erleichtern, ihre PID ohne Schreibfehler in die online-Fragebögen einzutragen. Es werden lediglich Telefonnummer und Emailadresse der Proband*innen erfragt, keine Adressdaten oder andere personenbezogene Daten. Um zu verhindern, dass Rückschlüsse auf einzelne Personen oder Personengruppen gezogen werden können, findet die Rekrutierung möglichst breit gestreut statt und werden zudem innerhalb der Hochschulen Studierende und Mitarbeitende unterschiedlicher Fachrichtungen und Hochschulen rekrutiert.

3.3 Güterabwägung

Von den geplanten Untersuchungen sind für die Probanden bzw. Befragten keine gesundheitlichen Risiken zu erwarten, weder von den Cortisol Proben und der Erfassung des

Hautleitwerts, noch von den Umfragen. Es werden keine anderen Parameter als Cortisol bei der Speichelanalyse bestimmt. Die Anonymität der Probanden/Befragten wird durch die individuellen Codes gewährleistet, so dass zu keinem Zeitpunkt des Projekts auf die Namen der Beteiligten geschlossen werden kann.

Frühere Forschung zeigt einige Evidenz für die Effekte von Lärm auf Stress und kognitive Leistung, aber es mangelt an Feldstudien zu den Effekten von Lärm auf Stress. Zudem hat bisher keine Studie die Effekte von Lärm auf Naturverbundenheit untersucht. Obwohl verschiedene frühere Studien den Effekt von Aufenthalten und Spaziergängen in naturnahen und urbanen Umgebungen verglichen haben, hat keine bisherige Studie die spezifischen Effekte von Lärm in diesem Kontext untersucht. Urbane und naturnahe Umgebungen in früheren Studien zum Einfluss von Natur könnten sich nicht nur anhand des Ausmaßes der Vegetation unterschieden haben, sondern auch im Hinblick auf Lärm. Unterschiede in Effekten, die der Vegetation zugesprochen werden, könnten demnach auch auf unterschiedliche Lärmpegel in urbanen und naturnahen Settings zurückgeführt werden. Die vorliegende Studie soll hierüber Aufschluss geben. Im Hinblick auf Achtsamkeit existiert eine Vielzahl an Studien, die positive Effekte von Achtsamkeitsintervention auf Stress und Wohlbefinden zeigen. Bisher mangelt es jedoch an Studien, die Achtsamkeitsinterventionen in naturnahen Settings untersuchen. In diesem Zusammenhang zielt die vorgelegte Studie darauf ab herauszufinden, inwiefern es nicht nur darauf ankommt *ob* Menschen sich in der Natur aufhalten, sondern *wie* sie sich in dieser aufhalten.

4. Literatur

Beierlein, C.; Kovaleva, A.; László, Z.; Kemper, C. J. & Rammstedt, B. (2014). Eine Single-Item-Skala zur Erfassung der Allgemeinen Lebenszufriedenheit: Die Kurzskala Lebenszufriedenheit-1 (L-1). GESIS Working Papers 33. Köln: GESIS – Leibniz-Institut für Sozialwissenschaften.

Bishop, S., Lau, M., Shapiro, S., Carlson, L., Anderson, N., Carmody, J., et al. (2004). Mindfulness: a proposed operational definition. *Clinical Psychology* 11:230–241.

Brown, W. & Ryan, M. (2003). The Benefits of Being Present: Mindfulness and Its Role in Psychological Well-Being. *Journal of Personality and Social Psychology* 84(4):822–848.

Creswell, D. (2017). Mindfulness Interventions. *Annual Review of Psychology* 68:491–516.

De Young, R. (2016). Necker Cube Test Introduction. Available online at: http:// seas.umich.edu/eplab/demos/nt0/neckerintro.html (accessed May 4, 2021).

Ehring, T.; Zetsche, U.; Weidacker, K.; Wahl, K.; Schönfeld, S. & Ehlers, A. (2011). The Perseverative Thinking Questionnaire (PTQ): Validation of a content-independent measure of repetitive negative thinking. Journal of Behavioral Therapy and Experimental Psychiatry 42(2):225–232. https://www.psy.lmu.de/klin/instrumente/ptq/index.htm

Gotnik, R., Chu, P., Busschbach, J., Benson, H., Fricchione, G. & Hunik, M. (2015). Standardised Mindfulness-Based Interventions in Healthcare: An Overview of Systematic Reviews and Meta-Analyses of RCTs. *PLoS ONE* 10(4):e0124344.

Govern, J. M. & Marsch, L. A. (2001). Development and Validation of the Situational Self-Awareness Scale. Consciousness and Cognition 10:366–378.

Grossman, P., Kappos, L., Gensicke, H., D'Souza, M., Mohr, D.C., Penner, I.K. & Steiner, C. (2010). MS quality of life, depression, and fatigue improve after mindfulness training: A randomized trial. *Neurology* 75(13):1141–1149.

Guski, R., Schreckenberg, D., Schuemer, R., (2017). WHO environmental noise guidelines for the European region: a systematic review on environmental noise and annoyance. *Int. J. Environ. Res. Public Health* 14, 1539, 1-41.

Hanley, A. Warner, A. & Garland, E.L. (2015). Associations Between Mindfulness, Psychological Well-Being, and Subjective Well-Being with Respect to Contemplative Practice. *Journal of Happiness Studies* 16:1423–1436.

Hofmann, M.; Young, C.; Binz, T. M.; Baumgartner, M. R. & Bauer, N. (2018). Contact to Nature Benefits Health: Mixed Effectiveness of Different Mechanisms. *International Journal of Environmental Research and Public Health 15*(1), 31(16 pp.). https://doi.org/10.3390/ijerph15010031

Höfling, V.; Ströhle, G.; Michalak, J. & Heidenreich, T. (2011). A Short Version of the Kentucky Inventory of Mindfulness Skills. Journal of Clinical Psychology 67(6):639-645.

Leiner, D. J. (2014). SoSci Survey [Computersoftware]. Verfügbar unter: https://www.soscisurvey.de.

Mayer, F. S. & Frantz, C. M. (2009). Why is Nature Beneficial? The Role of Connectedness to Nature. Environment and Behavior 41: 607. DOI: 10.1177/0013916508319745

Nyklíček, I. & Kuijpers, K. (2008). Effects of Mindfulness-Based Stress Reduction Intervention on Psychological Well-being and Quality of Life: Is Increased Mindfulness Indeed the Mechanism? *Annals of Behavioral Medicine* 35:331–340.

Nisbet, E.K., Zelenski, J.M. & Murphy, S.A. (2009): The nature relatedness scale. Linking individuals'connection with nature to environmental concern and behavior. Environment and Behavior, 5 (41), 715-740.

Nolen-Hoeksema ,S.; Wisco, B.E. & Lyubomirsky S. (2008). Rethinking Rumination. *Perspect Psychol Sci*. 3(5):400-24. doi: 10.1111/j.1745-6924.2008.00088.x. PMID: 26158958.

Parsons, C., Crane, C., Parsons, L., Fjorback, L. & Kuyken, W. (2017). Home practice in Mindfulness-Based Cognitive Therapy and Stress Reduction: A systematic review and meta-analysis of participants' mindfulness practice and its association with outcomes. *Behaviour Rerearch and Therapy* 95:29–41.

Perkins, H. E. (2010). Measuring love and care for nature. *Journal of Environmental Psychology, 30,* 455-463.

Schäffer, B., Brink, M., Schlatter, F., Vienneau, D., Wunderli, J.-M., (2020). Residential green is associated with reduced annoyance to road traffic and railway noise but increased annoyance to aircraft noise exposure. *Environment International* 42, 105885. DOI: 10.1016/j.envint.2020.105885

Schulz, P.; Schlotz, W. & Becker, P. (2004). TICS. Trierer Inventar zum chronischen Stress. Manual. Göttingen: Hogrefe Verlag.

Shapiro, S., Oman, D., Thoresen, C., Plante, T. & Flinders, T. (2008). Cultivating mindfulness: Effects on wellbeing. *Journal of Clinical Psychology* 64:840–862.

Tanay, G., & Bernstein, A. (2013). State mindfulness scale (SMS): Development and initial validation. *Psychological Assessment*, 25(4), 1286-1299.

Tyvräinen, L., Ojala, A., Korpela, K., Lanki, T., Tsunetsugu, Y. & Kagawa, T. (2014). The influence of urban green environments on stress relief measures: A field experiment. *Journal of Environmental Psychology,* 38:1-9.

Watson, D., Clark, L. A., & Tellegen, A. (1988). Development and validation of brief measures of positive and negative affect: The PANAS scales. *Journal of Personality and Social Psychology* 54(6), 1063-1070. doi: 10.1037/0022-3514.54.6.1063

Zylstra, M. J.; Knight, A. T.; Karen, J. E. & Lesley L.G. (2014). Connectedness as a Core Conservation Concern: An Interdisciplinary Review of Theory and a Call for Practice. Springer Science Reviews (2014) 2:119–143 DOI 10.1007/s40362-014-0021-3

C. Anhang

Das vorliegende Gesuch enthält die folgenden Beilagen:

C1: Einladungsbrief zur explorativen Umfrage in WP2

C2: Offene Fragen für Teilnehmende in der Naturbedingung der explorativen Umfrage in WP2

C3: Offene Fragen für Teilnehmende in der urbanen Bedingung der explorativen Umfrage in WP2

C4: Rekrutierungsschreiben für Feldstudie

C5: Teilnehmendeninformation für Feldstudie

C6: Necker-Cube Aufmerksamkeitstest

C7: T1 Fragebogen für online-Umfrage in WP2

C8: T2 Fragebogen für online-Umfrage in WP2

C9: T3 Fragebogen für online-Umfrage in WP2

C10: Achtsamkeitsintervention

C11: Mustervertrag Datenverarbeitungsvertrag im Auftrag nach EU-Datenschutzgrundverordnung (DSGVO)

C1: Einladungsbrief zur explorativen Befragung in WP2

Liebe Teilnehmerin, lieber Teilnehmer,

Im Rahmen eines Forschungsprojekts zum Einfluss von Natur und Geräuschen auf das menschliche Wohlbefinden, führen wir eine **Studie zu den Effekten von Spazierengehen in unterschiedlich lärmbelasteten Umgebungen** durch. In Vorbereitung auf die Studie befragen wir dafür Menschen nach ihren **Erfahrungen beim Spazierengehen** in verschiedenen Umgebungen. **Wir möchten Sie herzlich einladen, an der Studie mitzuwirken.**

Um an der Voruntersuchung teilzunehmen, gehen Sie bitte 20-30 Min. an einem Ort Ihrer Wahl in der Natur spazieren, den Sie selbst wählen können (beispielsweise im Wald). Sie sollten allein und ohne Kopfhörer im Ohr unterwegs sein. Im Anschluss berichten Sie bitte unter nachfolgenden Link von Ihren Erfahrungen beim Spaziergang: https://www.soscisurvey.de/RESTOREvu/

Die Untersuchung ist **anonym** und **vertraulich**. Durch die Beteiligung an der Studie können Sie an der Erforschung von förderlichen und hinderlichen Faktoren für Erholung bei Spaziergängen mitwirken und einen wertvollen Beitrag zur aktuellen Forschung leisten.

Bei Fragen kontaktiert uns gerne. Vielen Dank für eure Mithilfe!

Julia Schaupp

Julia Schaupp M.Sc. | Doktorandin

Universität Basel | Fakultät für Psychologie | Klinische Psychologie und Psychotherapie
julia.schaupp@unibas.ch

Eidg. Forschungsanstalt für Wald, Schnee und Landschaft WSL
Zürcherstrasse 111 | CH-8903 Birmensdorf
Telefon +41-44-739 2484 | julia.schaupp@wsl.ch | http://www.wsl.ch/

C2: Offene Fragen für Teilnehmende in der Naturbedingung der explorativen Umfrage in WP2

Liebe Teilnehmerin, lieber Teilnehmer,

herzlich willkommen und vielen Dank, dass Sie an unserer Studie mitwirken möchten!

Dies ist eine Voruntersuchung für eine Studie zu den Effekten von Spazierengehen in unterschiedlich lärmbelasteten Umgebungen. Die Studie ist Teil des Forschungsprojekts RESTORE, das von der Eidg. Forschungsanstalt für Wald, Schnee und Landschaft (WSL) koordiniert und durch den Schweizerischer Nationalfonds (SNF) finanziert wird. Das Projekt soll Aufschluss über das Erholungspotenzial unterschiedlicher Umgebungen geben.

Um an der Voruntersuchung teilzunehmen, gehen Sie bitte ca. 20 Min. an einem Ort Ihrer Wahl in der Natur spazieren. Im Anschluss beantworten Sie bitte die nachfolgenden Fragen. Alle erhobenen Daten werden anonym und vertraulich behandelt.

Herzlichen Dank für Ihre Bemühungen!

<u>Für Rückfragen zum Forschungsprojekt wenden Sie sich an:</u>

M.Sc. Julia Schaupp, Eidg. Forschungsanstalt für Wald, Schnee und Landschaft WSL, Zürcherstr. 111, CH-8903 Birmensdorf. julia.schaupp@wsl.ch, Telefon +41-44-739 2484.

Teilnahmebedingungen und Datenschutz

Die Teilnahme an dieser Studie ist freiwillig, die Ergebnisse der Studie werden für wissenschaftliche Zwecke verwendet. Es sind keine Risiken mit dieser Studie verbunden. Sie können die Studie jederzeit ohne Angaben von Gründen abbrechen. Hierdurch entstehen Ihnen keine Nachteile. Alle Daten werden streng vertraulich gehalten. Es sind keine Rückschlüsse auf Ihre Person möglich.

☐ Ich bestätige, dass ich die Teilnahmebedingungen gelesen und verstanden habe und möchte an der Studie teilnehmen.

Zunächst interessieren wir uns für den Spaziergang, den Sie gerade unternommen haben.

Welche Wirkung hat der Spaziergang auf Sie gehabt? Wie fühlen Sie sich danach im Vergleich zu vor dem Spaziergang?

Hat der Spaziergang Ihnen gutgetan? Falls ja, woran merken Sie, dass er Ihnen gutgetan hat? Falls nein, woran merken Sie, dass er Ihnen nicht gutgetan hat?

Wie haben Sie die Umgebung, in der Sie spazieren gegangen sind erlebt?

Was haben Sie während des Spaziergangs gehört?

Welche Gefühle hat die Geräuschkulisse während des Spaziergangs bei Ihnen ausgelöst?

Wie hat sich die Geräuschkulisse während des Spaziergangs auf Ihre Beziehung zur Natur ausgewirkt?

Worüber denken Sie üblicherweise bei Alltagstätigkeiten nach? Worüber haben Sie beim Spaziergang nachgedacht? Haben Sie Unterschiede in den Inhalten oder der Art der Gedanken bemerkt?

Nun interessieren wir uns für Ihren Alltag im Allgemeinen. Was sind Stressfaktoren in Ihrem Alltag?

Was tun Sie normalerweise, um sich von Stress zu erholen?

Welche Bedeutung hat in der Natur sein für Sie im Alltag?

Was hilft Ihnen bei Umgang mit persönlichen Problemen und Sorgen?

Hilft Spazierengehen in der Natur Ihnen beim Umgang mit persönlichen Problemen? Falls ja, inwiefern hilft es und woran merken Sie, dass es hilft? Falls nein, woran merken Sie, dass es nicht hilft?

Hat sich die Bedeutung von Natur für Sie in der Corona-Zeit verändert? Fall ja, inwiefern hat sie sich verändert?

Hat sich Ihre Nutzung von Grünräumen im Zuge der Corona-Situation verändert?

Möchten Sie sonst noch etwas anmerken?

Vielen Dank für Ihre Teilnahme!

Wir möchten uns ganz herzlich für Ihre Mithilfe bedanken.

Ihre Antworten wurden gespeichert, Sie können das Browser-Fenster nun schließen.

C3: Offene Fragen für Teilnehmende in der urbanen Bedingung der explorativen Umfrage in WP2

Liebe Teilnehmerin, lieber Teilnehmer,

herzlich willkommen und vielen Dank, dass Sie an unserer Studie mitwirken möchten!

Dies ist eine Voruntersuchung für eine Studie zu den Effekten von Spazierengehen in unterschiedlich lärmbelasteten Umgebungen. Die Studie ist Teil des Forschungsprojekts RESTORE, das von der Eidg. Forschungsanstalt für Wald, Schnee und Landschaft (WSL) koordiniert und durch den Schweizerischer Nationalfonds (SNF) finanziert wird. Das Projekt soll Aufschluss über das Erholungspotenzial unterschiedlicher Umgebungen geben.

Um an der Voruntersuchung teilzunehmen, gehen Sie bitte ca. 20 Min. an einem Ort Ihrer Wahl in der Stadt spazieren. Im Anschluss beantworten Sie bitte die nachfolgenden Fragen. Alle erhobenen Daten werden anonym und vertraulich behandelt.

Herzlichen Dank für Ihre Bemühungen!

Für Rückfragen zum Forschungsprojekt wenden Sie sich an:

M.Sc. Julia Schaupp, Eidg. Forschungsanstalt für Wald, Schnee und Landschaft WSL, Zürcherstr. 111, CH-8903 Birmensdorf. julia.schaupp@wsl.ch, Telefon +41-44-739 2484.

Teilnahmebedingungen und Datenschutz

Die Teilnahme an dieser Studie ist freiwillig, die Ergebnisse der Studie werden für wissenschaftliche Zwecke verwendet. Es sind keine Risiken mit dieser Studie verbunden. Sie können die Studie jederzeit ohne Angaben von Gründen abbrechen. Hierdurch entstehen Ihnen keine Nachteile. Alle Daten werden streng vertraulich gehalten. Es sind keine Rückschlüsse auf Ihre Person möglich.

☐ Ich bestätige, dass ich die Teilnahmebedingungen gelesen und verstanden habe und möchte an der Studie teilnehmen.

Zunächst interessieren wir uns für den Spaziergang, den Sie gerade unternommen haben.

Welche Wirkung hat der Spaziergang auf Sie gehabt? Wie fühlen Sie sich danach im Vergleich zu vor dem Spaziergang?

Hat der Spaziergang Ihnen gut getan? Falls ja, woran merken Sie, dass er Ihnen gut getan hat? Falls nein, woran merken Sie, dass er Ihnen nicht gut getan hat?

Wie haben Sie die Umgebung, in der Sie spazieren gegangen sind erlebt?

Was haben Sie während des Spaziergangs gehört?

Welche Gefühle hat die Geräuschkulisse während des Spaziergangs bei Ihnen ausgelöst?

Wie hat sich die Geräuschkulisse während des Spaziergangs auf Ihre Beziehung zur Umgebung ausgewirkt?

Worüber denken Sie üblicherweise bei Alltagstätigkeiten nach? Worüber haben Sie beim Spaziergang nachgedacht? Haben Sie Unterschiede in den Inhalten oder der Art der Gedanken bemerkt?

Nun interessieren wir uns für Ihren Alltag im Allgemeinen. Was sind Stressfaktoren in Ihrem Alltag?

Was tun Sie normalerweise, um sich von Stress zu erholen?

Was hilft Ihnen bei Umgang mit persönlichen Problemen und Sorgen?

Welche Bedeutung hat in der Natur sein für Sie im Alltag?

Hilft Spazierengehen in der Natur Ihnen beim Umgang mit persönlichen Problemen? Falls ja, inwiefern hilft es und woran merken Sie, dass es hilft? Falls nein, woran merken Sie, dass es nicht hilft?

Hat sich die Bedeutung von Natur für Sie in der Corona-Zeit verändert? Fall ja, inwiefern hat sie sich verändert?

Hat sich Ihre Nutzung von Grünräumen im Zuge der Coronakrise verändert?

Möchten Sie sonst noch etwas anmerken?

Vielen Dank für Ihre Teilnahme!

Wir möchten uns ganz herzlich für Ihre Mithilfe bedanken.

Ihre Antworten wurden gespeichert, Sie können das Browser-Fenster nun schließen.

C4: Rekrutierungsschreiben für die Feldstudie

Studie zu förderlichen und hinderlichen Faktoren für die Erholung bei halbstündigen Spaziergängen

Sehr geehrte Damen und Herren, liebe Studierende, liebe Mitarbeitende,

Im Rahmen eines vom Schweizerischen Nationalfonds finanzierten Forschungsprojekts zum Einfluss von Natur und Geräuschen auf das menschliche Wohlbefinden, das von der Eidg. Forschungsanstalt für Wald, Schnee und Landschaft (WSL) koordiniert wird, führen wir eine Studie zu den Effekten von Spazierengehen in unterschiedlich lärmbelasteten Umgebungen durch.

Wir möchten Sie herzlich einladen, an der Studie teilzunehmen!

Die Studie erforscht, welchen Einfluss Verkehrslärm und Vegetation für die Erholung bei halbstündigen Spaziergängen haben. Zudem wird in der Studie untersucht, inwiefern neben Umgebungsfaktoren auch die innere Haltung, mit der Menschen der Umgebung beim Spazierengehen begegnen, einen Einfluss auf die Erholung bei Spaziergängen hat.

Helfen Sie uns mit Ihrer Studienteilname dabei herauszufinden, welche Faktoren die Erholung bei Spaziergängen ermöglichen oder behindern. Dank Ihrer Studienteilnahme können die Ergebnisse der Allgemeinbevölkerung zugutekommen – vor allem dann, wenn über städtische Natur und die Zukunft städtischer Flächen für Erholungszwecke verhandelt wird.

Bei der Studie geht es darum, einen halbstündigen, geleiteten Spaziergang in einer kleinen Gruppe (2-6 Personen) an einem vorgegebenen Ort zu machen. Vor, während und nach dem Spaziergang erfassen wir Ihre Erholung. Neben Angaben in einem Fragebogen messen wir dafür vor und nach dem Spaziergang das Hormon Cortisol, welches das Ausmass an Stress anzeigt. Zudem wird während des Spaziergangs die körperliche Anspannung mit Hilfe eines Geräts zur Erfassung des Hautleitwerts gemessen. Dafür werden Elektroden zur Erfassung des Hautleitwerts an einer Ihrer Hände angeklebt und während des Spaziergangs vor Ort getragen. Im Anschluss an den Spaziergang vor Ort werden wir Sie bitten, in den nächsten 10 Tagen noch dreimal individuell für eine halbe Stunde in einer Umgebung mit bestimmten Eigenschaften spazieren zu gehen.

Für die Teilnahme an der Studie erhalten Sie eine Aufwandsentschädigung in Höhe von 50 CHF oder falls Sie im Fach Psychologie studieren können Sie sich Versuchspersonen Stunden gutschreiben lassen.

Sie können an dieser Studie teilnehmen, wenn Sie

- älter als 18 Jahre sind.
- Sehr gut Deutsch verstehen.
- körperlich in der Lage sind, eine halbe Stunde in moderatem Tempo spazieren zu gehen.
- keine diagnostizierten Hörprobleme haben.
- kein Cortison einnehmen.
- bei Ihnen kein Cushing Syndrom vorliegt.
- Ihr BMI nicht über 35 liegt.

Haben Sie Interesse und/ oder sind noch Fragen offen? Dann melden Sie sich unter: restore-termin@wsl.ch oder per Telefon unter 0041447392484. Alternativ können Sie sich auch direkt unter folgendem Link mit Namen, E-Mailadresse und Telefonnummer **verbindlich** für einen Termin eintragen: Link einfügen.
Wenn Sie sich für die Teilnahme an der Studie entscheiden willigen Sie ein, dass wir Ihren Namen, Emailadresse und Telefonnummer für die Dauer des Experiments speichern. Ihre Angaben können nur von Projektmitarbeitenden gesehen werden und werden nicht an Dritte weitergegeben. Sie werden lediglich zur Kontaktaufnahme zwecks der Studienteilnahme verwendet.

Wir freuen uns auf Ihre Kontaktaufnahme!!!

Julia Schaupp und Nicole Bauer

Für Rückfragen zum Forschungsprojekt wenden Sie sich an:

Projektdurchführung: M.Sc. Julia Schaupp, Eidg. Forschungsanstalt für Wald, Schnee und Landschaft WSL, Zürcherstr. 111, CH-8903 Birmensdorf. Mail: julia.schaupp@wsl.ch, Tel: 044 739 2484)

Leiterin der Studie: Dr. Nicole Bauer, Eidg. Forschungsanstalt für Wald, Schnee und Landschaft WSL, Zürcherstr. 111, CH-8903 Birmensdorf. Mail: nicole.bauer@wsl.ch, Tel: 044 739 2458)

C5: Teilnehmendeninformation für Feldstudie

Allgemeine Teilnehmendeninformation „Studie zu förderlichen und hinderlichen Faktoren für die Erholung bei halbstündigen Spaziergängen“

1. Was ist das Ziel der Studie?

Verschiedene Studien deuten darauf hin, dass Spazierengehen in Abhängigkeit von der Umgebung, in der Menschen spazieren gehen sowie je nach Haltung, mit der Menschen der Umgebung begegnen unterschiedlich stark zu Erholung beitragen kann. In diesem Sinne untersucht die Studie unter welchen Bedingungen Spaziergänge eine erholsame Wirkung haben. Die Studie ist Teil des Forschungsprojekts RESTORE, das von der Eidg. Forschungsanstalt für Wald, Schnee und Landschaft (WSL) koordiniert und durch den Schweizerischen Nationalfonds (SNF) finanziert wird.
Das *erste Untersuchungsziel* der Studie besteht darin zu erforschen, welchen Einfluss Verkehrslärm und Vegetation für die Erholung beim Spazierengehen haben. Die Ergebnisse sollen Aufschluss über das Erholungspotenzial unterschiedlicher Umgebungen geben. Im Rahmen *des zweiten Untersuchungsziels* wird untersucht, ob neben Umgebungsfaktoren auch die innere Haltung, mit der Menschen der Umgebung beim Spazierengehen begegnen, einen Einfluss auf die Erholung bei Spaziergängen hat.

2. Wie sieht der Ablauf der Studie aus?

Die Studie umfasst einen halbstündigen, geleiteten Spaziergang in einer kleinen Gruppe (2-6 Personen) auf einer von uns vorgegebenen Strecke. Wenn Sie Interesse haben an der Studie teilzunehmen und wir einen Termin vereinbart haben, informieren wir Sie über den Treffpunkt, von dem aus der Spaziergang starten wird.
Vor Ort lesen Sie zunächst aufmerksam diese Teilnehmerinformation und werden dann aufgefordert Ihr Einverständnis zur Studienteilnahme zu geben. Anhand von Speichelproben messen wir vor und nach dem Spaziergang das Hormon Cortisol, welches das Ausmass an Stress anzeigt. Zudem wird während des Spaziergangs die körperliche Anspannung mit Hilfe eines Geräts zur Erfassung des Hautleitwerts gemessen. Dafür werden Elektroden zur Erfassung des Hautleitwerts an einer Ihrer Hände angeklebt und während des 30-minütigen Spaziergangs vor Ort getragen. Nach dem Spaziergang werden diese schmerzfrei wieder entfernt. Vor und nach dem Spaziergang bitten wir Sie ausserdem eine Aufgabe zu bearbeiten, die Aufmerksamkeit erfordert und fragen sie in einem Fragebogen nach Ihrem Befinden, mentalen Zuständen, Persönlichkeitseigenschaften, momentaner Aufmerksamkeit, Formen des Nachdenkens, demographischen Informationen und ihrem Eindruck von der Umgebung. Sie erhalten von uns eine persönliche ID-Nummer, die Sie in alle Fragebögen eintragen. Während des Spaziergangs folgen Sie einfach den Anweisungen der Studienleitung, sie weist Ihnen auch den Weg.
Im Anschluss an den Spaziergang vor Ort bitten wir Sie, in den nächsten 10 Tagen noch drei Mal für eine halbe Stunde in einer Umgebung mit bestimmten Eigenschaften spazieren zu gehen. Sie bekommen von der Studienleitung vor Ort genauere Informationen hierzu. Nach 10 Tagen senden wir Ihnen per E-Mail eine Einladung mit einem Link zu einem dritten Fragebogen zu, in den es erneut um Ihre Befindlichkeit geht. Wir führen die Studie im Einklang mit den Schweizer Gesetzen durch. Die Ethikkommission der ETH hat die Studie geprüft und bewilligt.

3. Welchen Nutzen hat die Studienteilnahme für Sie?

Die Eidgenössische Forschungsanstalt für Wald, Schnee und Landschaft WSL erarbeitet Grundlagen für eine bedürfnisgerechte, nachhaltige Landschaftsentwicklung und Raumplanung. Durch Ihre Teilnahme ermöglichen Sie uns die Untersuchung von förderlichen und hinderlichen

Faktoren wie beispielsweise Verkehrslärm und Vegetation für die Erholung bei Spaziergängen. Mit den Ergebnissen hoffen wir einen Beitrag leisten zu können, dass die Landschaft so gestaltet wird, dass auch wir Menschen uns darin wohl fühlen und erholen können. Dank Ihrer Studienteilnahme können die Ergebnisse der Allgemeinbevölkerung zugutekommen – vor allem dann, wenn über städtische Natur und die Zukunft städtischer Flächen für Erholungszwecke verhandelt wird. Mit der Studie sind keine bekannten Risiken oder Belastungen verbunden.

4. Rechte und Pflichten der Teilnehmenden

Sie nehmen an der Studie nur teil, wenn *Sie* es wollen. Sie können Ihren Entscheid jederzeit und ohne Begründung zurücknehmen. Sie dürfen jederzeit Fragen zur Studie stellen; unsere Kontaktdaten finden Sie am Ende dieser Studieninformation.

5. Vertraulichkeit der Daten und Proben

Für diese Studie erfassen wir Daten zu Ihrer Befindlichkeit, Persönlichkeit und weiteren Eigenschaften (siehe oben). Zudem untersuchen wir Ihren Cortisol- und Hautleitwert. Diese Daten und Proben werden anonymisiert. Das bedeutet, dass wir alle Angaben, die Sie identifizieren könnten durch einen Code ersetzen, der keine Rückschlüsse auf Ihre Person ermöglicht. Wir werden Ihnen deshalb Ihre persönlichen Ergebnisse nicht mitteilen können. Ihren Namen werden wir nicht veröffentlichen, weder in einem Bericht, einer Publikation, noch in sonst einer Weise gedruckt oder im Internet. Alle Personen, die mit der Studie in irgendeiner Weise zu tun haben, verpflichten sich zu absoluter Vertraulichkeit. Am Ende der Studie vernichten wir die Proben, die Daten bewahren wir nach den gesetzlichen Vorgaben auf. Die Teilnahme an der Studie ist freiwillig.

6. Deckung von Schäden

Allfällige Gesundheitsschäden, die in direktem Zusammenhang mit der Studie entstehen und auf Verschulden der WSL zurückzuführen sind, sind durch eine Betriebshaftpflichtversicherung gedeckt (Basler Versicherungen, Police Nr. 30 5 007 450 6). Der Abschluss einer Unfall- /Krankenversicherung liegt in der Verantwortung des Probanden. Wenn Sie einen Schaden erlitten haben, wenden Sie sich bitte an die Leiterin der Studie.

7. Kontaktpersonen

Bei Unklarheiten oder Bedenken, die während oder nach der Studie auftreten, können Sie sich jederzeit an eine der folgenden Kontaktpersonen wenden:

Ansprechpersonen für Fragen zur Studie:
Studiendurchführung: Julia Schaupp (julia.schaupp@wsl.ch, Tel.: 044 739 24 84)
Leiterin der Studie: Dr. Nicole Bauer (nicole.bauer@wsl.ch, Tel: 044 739 24 58)

Adresse:
Eidg. Forschungsanstalt für Wald, Schnee und Landschaft WSL
Forschungsgruppe «Sozialwissenschaftliche Landschaftsforschung»
Zürcherstrasse 111, 8903 Birmensdorf

Schriftliche Einwilligungserklärung zur Teilnahme an der Studie zu förderlichen und hinderlichen Faktoren für die Erholung bei halbstündigen Spaziergängen

- Bitte lesen Sie diese Seite sorgfältig durch.
- Bitte fragen Sie, wenn Sie etwas nicht verstehen. Wir geben gerne Auskunft.

**Titel der Studie**	„Hinderliche und förderliche Faktoren für die Erholung bei Spaziergängen in unterschiedlichen Umgebungen"
**Verantwortliche Institution**	Eidg. Forschungsanstalt für Wald, Schnee und Landschaft WSL, Zürcherstrasse 111, 8903 Birmensdorf
**Ort der Durchführung**	Raum Zürich
**Leiterin der Studie**	Dr. Nicole Bauer
**Teilnehmerin/Teilnehmer**	Vollständiger Name:<br>Geburtsdatum:

- Ich wurde über den Zweck und Ablauf der Studie sowie über eventuelle Risiken informiert.
- Ich nehme an dieser Studie freiwillig teil. Ich kann jederzeit und ohne Angabe von Gründen meine Zustimmung zur Teilnahme widerrufen, ohne dass mir deswegen Nachteile entstehen.
- Ich habe das Dokument mit der Teilnehmendeninformation gelesen. Meine Fragen im Zusammenhang mit der Teilnahme an dieser Studie sind mir zufriedenstellend beantwortet worden. Ich kann die schriftliche Studieninformation behalten, ebenso wie eine Kopie dieser Einwilligungserklärung.
- Ich akzeptiere den Inhalt der zur oben genannten Studie abgegebenen schriftlichen Studieninformation.
- Ich hatte genügend Zeit, meine Entscheidung zu treffen.
- Ich bin darüber informiert, dass eine Versicherung Schäden deckt, die mir durch Teilnahme an dieser Studie entstanden sind.
- Mit meiner Unterschrift bestätige ich, dass ich die im Informationsblatt genannten Voraussetzungen für die Studienteilnahme erfülle.
- Ich bin darüber informiert, dass eine Versicherung Schäden deckt, die in direktem Zusammenhang mit der Studie entstehen und auf Verschulden der WSL zurückzuführen sind.
- Ich weiss, dass meine persönlichen Daten und Proben nur in anonymisierter Form für dieses Forschungsprojekt genutzt werden. Ich bin einverstanden, dass die zuständigen Fachleute des Auftraggebers der Studie, der Behörden und der für diese Studie zuständigen Ethikkommission zu Prüf- und Kontrollzwecken in meine Originaldaten Einsicht nehmen dürfen, jedoch unter strikter Einhaltung der Vertraulichkeit.

Ort, Datum	Unterschrift Studienteilnehmerin/Studienteilnehmer

C6: Necker Cube Pattern Control Test

Instruktion:

Das ist ein Würfel ohne Tiefeninformation. Man kann den Würfel aus unterschiedlichen Perspektiven sehen. Die Perspektive tendiert dazu zu springen, wenn man länger auf den Würfel schaut. Die Vorder- und Hinterseite des Würfels ändern ihre relative Position.

Bitte schauen Sie jetzt im Anschluss auf die Würfelfigur und klopfen Sie jedes Mal, wenn der Würfel aus Ihrer Sicht die Perspektive wechselt mit dem Stift auf den Tisch. Ich werde Ihnen sagen, wann Sie beginnen und wann Sie aufhören sollen.

Haben Sie Fragen? Starten Sie.

Dieses Mal versuchen Sie, so lange wie möglich die aktuelle Perspektive des Würfels zu halten. Klopfen Sie jedes Mal, wenn die Perspektive wechselt. Ich sage Ihnen wann Sie anfangen und aufhören sollen. Also: Versuchen Sie die Perspektive die Sie aktuell sehen jeweils so lange wie möglich zu halten, klopfen Sie bei jedem Wechsel.

Haben Sie Fragen? Starten Sie.

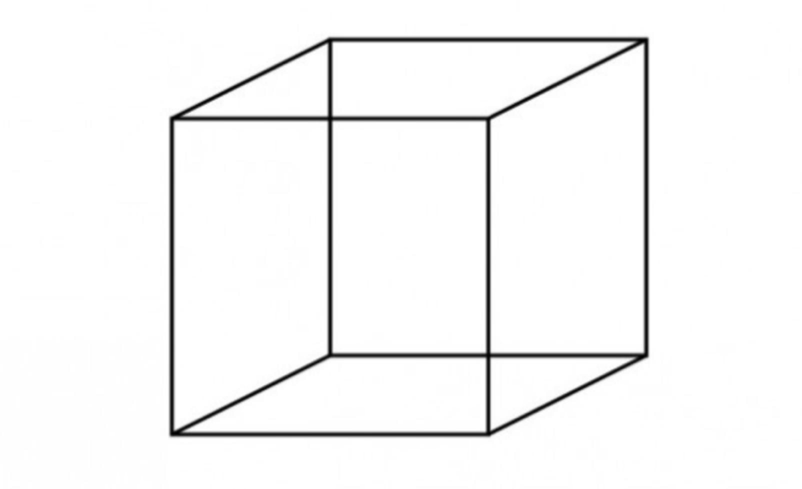

Beispiele der unterschiedlichen Perspektiven:

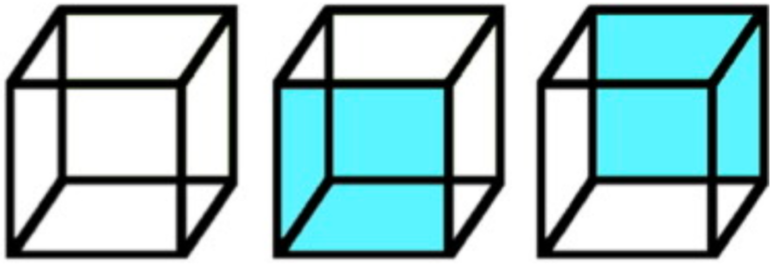

C7: T1 Fragebogen für online-Umfrage in WP2

Liebe Teilnehmerin, lieber Teilnehmer,

herzlich willkommen und vielen Dank, dass Sie an unserer Studie mitwirken!

Dies ist eine Studie zu den Effekten von Spazierengehen in unterschiedlich lärmbelasteten Umgebungen. Die Studie ist Teil des Forschungsprojekts RESTORE, das von der Eidg. Forschungsanstalt für Wald, Schnee und Landschaft (WSL) koordiniert wird. Das Projekt soll Aufschluss über das Erholungspotenzial unterschiedlicher Umgebungen geben.

Um Veränderungen und Entwicklungen über die Zeit zu messen, bitten wir Sie **dreimal** Fragen zu beantworten: **vor Beginn des Spaziergangs** heute, zum **Ende des Spaziergangs** sowie ein weiteres Mal **in 10 Tagen.**

Die Bearbeitung des Fragebogens wird etwa 15-20 Minuten in Anspruch nehmen. Alle erhobenen Daten werden anonym und vertraulich behandelt.

Herzlichen Dank für Ihre Bemühungen!

<u>Für Rückfragen zum Forschungsprojekt wenden Sie sich an:</u>

M.Sc. Julia Schaupp, Eidg. Forschungsanstalt für Wald, Schnee und Landschaft WSL, Zürcherstr. 111, CH-8903 Birmensdorf. julia.schaupp@wsl.ch, Telefon +41-44-739 2484.

Teilnahmebedingungen und Datenschutz

Die Teilnahme an dieser Studie ist freiwillig, die Ergebnisse der Studie werden für wissenschaftliche Zwecke verwendet. Es sind keine Risiken mit dieser Studie verbunden. Sie können die Studie jederzeit ohne Angaben von Gründen abbrechen. Hierdurch entstehen Ihnen keine Nachteile.

Alle Daten werden streng vertraulich gehalten. Es sind keine Rückschlüsse auf Ihre Person möglich. Am Ende des Fragebogens werden Sie aufgefordert, Ihre Emailadresse anzugeben. An diese Emailadresse wird Ihnen in zwei Wochen eine Einladung zu einer weiteren Befragung zugeschickt. Die Emailadresse wird getrennt von Ihren sonstigen Daten zum Fragebogen verarbeitet.

☐ Ich bestätige, dass ich die Teilnahmebedingungen gelesen und verstanden habe und möchte an der Studie teilnehmen.

Bitte geben Sie Ihren vierstelligen persönlichen ID-Code an. Achten Sie dabei auf Groß- und Kleinschreibung.

ID-Code

Kommentar für Ethikkommission. *Erfasstes Konzept: Achtsamkeit State. Standardisierte Skala (State Mindfulness Scale; Tanay & Bernstein, 2013)*

Im Folgenden sehen Sie eine Liste von Aussagen. Bitte geben Sie an, in welchem Ausmaß die folgenden Aussagen *während der letzten halben Stunde* auf sie zugetroffen haben.

	überhaupt nicht				sehr
Ich war mir der verschiedenen Gefühle bewusst, die in mir aufkamen.	○	○	○	○	○
Ich habe versucht, meine Aufmerksamkeit auf angenehme und unangenehme Empfindungen zu richten.	○	○	○	○	○
Ich fand einige meiner Erfahrungen interessant.	○	○	○	○	○
Ich habe viele kleine Details meiner Erfahrung wahrgenommen.	○	○	○	○	○
Ich war mir dessen bewusst, was in mir vorging.	○	○	○	○	○
Ich habe angenehme und unangenehme Gefühle wahrgenommen.	○	○	○	○	○
Ich habe aktiv meine Erfahrung im aktuellen Moment erforscht.	○	○	○	○	○
Ich habe klar gespürt, was in meinem Körper vor sich ging.	○	○	○	○	○
Ich habe meine Körperhaltung verändert und den körperlichen Prozess der Bewegung wahrgenommen.	○	○	○	○	○
Ich hatte das Gefühl, den gegenwärtigen Moment vollständig zu erleben.	○	○	○	○	○
Ich habe angenehme und unangenehme Gedanken wahrgenommen.	○	○	○	○	○
Ich habe wahrgenommen, wie Gefühle kommen und gehen.	○	○	○	○	○
Ich habe verschiedene Empfindungen wahrgenommen, die durch meine Umgebung hervorgerufen wurden (z.B. Hitze, Kälte, den Wind auf meinem Gesicht).	○	○	○	○	○
Ich habe körperliche Empfindungen kommen und gehen gespürt.	○	○	○	○	○
Ich hatte Momente, an denen ich mich wach und bewusst gefühlt habe.	○	○	○	○	○
Ich habe mich eng mit dem gegenwärtigen Moment verbunden gefühlt.	○	○	○	○	○
Ich habe Gedanken wahrgenommen, die kommen und gehen.	○	○	○	○	○
Ich habe mich mit meinem Körper in Kontakt gefühlt.	○	○	○	○	○
Ich war mir dessen, was in meinem Geist vor sich ging bewusst.	○	○	○	○	○
Es war interessant, meine Denkmuster wahrzunehmen.	○	○	○	○	○
Ich habe manche angenehmen und unangenehmen körperlichen Erfahrungen bemerkt.	○	○	○	○	○

Kommentar für Ethikkommission. Erfasstes Konzept: Wohlbefinden. Standardisierte Skala (Positive and Negative Affect Schedule; Watson et al., 1988).

Nun möchten wir gerne von Ihnen wissen, wie Sie sich fühlen.

Die folgenden Wörter beschreiben unterschiedliche Gefühle und Empfindungen. Lesen Sie jedes Wort und tragen Sie dann in die Skala neben jedem Wort die Intensität ein. Sie haben die Möglichkeit, zwischen fünf Abstufungen zu wählen. Geben Sie bitte an, wie Sie sich momentan fühlen.

	gar nicht	ein bisschen	einigermaßen	erheblich	äußerst
aktiv	○	○	○	○	○
bekümmert	○	○	○	○	○
interessiert	○	○	○	○	○
freudig erregt	○	○	○	○	○
verärgert	○	○	○	○	○
stark	○	○	○	○	○
schuldig	○	○	○	○	○
erschrocken	○	○	○	○	○
feindselig	○	○	○	○	○
angeregt	○	○	○	○	○
stolz	○	○	○	○	○
gereizt	○	○	○	○	○
begeistert	○	○	○	○	○
beschämt	○	○	○	○	○
wach	○	○	○	○	○
nervös	○	○	○	○	○
entschlossen	○	○	○	○	○
aufmerksam	○	○	○	○	○
durcheinander	○	○	○	○	○
ängstlich	○	○	○	○	○

*Kommentar für Ethikkommission. Erfasstes Konzept: Trait-Achtsamkeit. Standardisierte Skala (*Kentucky Inventory for Mindfulness Skills Short; Höfling et al., 2011*).*

Schätzen Sie bitte jede der folgenden Aussagen mit Hilfe der bereitgestellten Skala ein. Wählen Sie dabei die Ziffer aus, die im Allgemeinen am ehesten auf Sie zutrifft. Bitte antworten Sie spontan, ohne lange darüber nachzudenken, und so, wie Sie die Dinge tatsächlich erleben und nicht, wie Sie sie gerne erleben würden.

	trifft nie oder sehr selten zu	trifft selten zu	trifft manchmal zu	trifft oft zu	trifft sehr oft oder immer zu
Ich kann meine Gefühle gut in Worte fassen.	○	○	○	○	○
Ich kritisiere mich dafür, irrationale oder unangebrachte Gefühle zu haben.	○	○	○	○	○
Ich konzentriere mich nur auf das, was ich gerade tue und auf nichts anderes.	○	○	○	○	○
Wenn ich gehe, dann nehme ich ganz bewusst wahr, wie sich die Bewegungen meines Körpers anfühlen.	○	○	○	○	○
Wenn ich dusche oder bade, bin ich mir dieses Gefühls des Wassers auf meinem Körper bewusst.	○	○	○	○	○
Es fällt mir schwer, das, was ich denke, in Worte zu fassen.	○	○	○	○	○
Ich glaube, dass einige meiner Gedanken unnormal sind und dass ich nicht so denken sollte.	○	○	○	○	○
Ich habe Schwierigkeiten, die richtigen Worte zu finden, um meine Gefühle auszudrücken.	○	○	○	○	○
Wenn ich etwas tue, dann bin ich davon völlig eingenommen und denke an nichts anderes mehr.	○	○	○	○	○
Ich urteile darüber, ob meine Gedanken gut oder schlecht sind.	○	○	○	○	○
Ich achte auf meine Empfindungen, wie zum Beispiel Wind in meinem Haar oder Sonnenschein auf meinem Gesicht.	○	○	○	○	○
Körperliche Empfindungen sind für mich schwer zu beschreiben, weil mir die richtigen Worte dazu fehlen.	○	○	○	○	○
Ich achte auf Geräusche, wie beispielsweise das Ticken von Uhren, Vogelzwitschern oder das Geräusch vorüberfahrender Autos.	○	○	○	○	○
Sogar wenn ich schrecklich verärgert bin, kann ich das in Worte fassen.	○	○	○	○	○
Ich sage mir, dass ich nicht so denken sollte, wie ich denke.	○	○	○	○	○
Ich nehme Gerüche und Düfte der Dinge wahr.	○	○	○	○	○
Ich neige dazu mehrere Dinge gleichzeitig zu tun, anstatt mich nur auf eine Sache zu konzentrieren.	○	○	○	○	○
Ich denke, dass manche meiner Gefühle schlecht oder unangebracht sind und dass ich sie nicht haben sollte.	○	○	○	○	○
Ich bemerke visuelle Elemente sowohl in der Kunst als auch in der Natur, zum Beispiel Farben, Formen, Struktur oder Muster aus Licht und Schatten.	○	○	○	○	○
Wenn ich etwas tue, werde ich so davon eingenommen, dass meine ganze Aufmerksamkeit darauf gerichtet ist.	○	○	○	○	○

Kommentar für Ethikkommission. Erfasstes Konzept: emotionale Komponente Naturverbundenheit*. Standardisierte Skala (*Love and Care for Nature Scale; Perkins, 2010*).*

Inwiefern treffen die folgenden Aussagen auf Sie zu?

	stimme überhaupt nicht zu						stimme voll und ganz zu
Ich empfinde eine tiefe Liebe zur Natur.	○	○	○	○	○	○	○
Der Schutz der Natur und ihr Wohlergehen ist mir wichtig.	○	○	○	○	○	○	○
Ich empfinde oft ein Gefühl von Ehrfurcht und Erstaunen, wenn ich in unberührter Natur bin.	○	○	○	○	○	○	○
Ich empfinde oft ein starkes Bedürfnis der Fürsorge gegenüber der natürlichen Umgebung.	○	○	○	○	○	○	○
Ich fühle mich der Natur oft emotional nahe.	○	○	○	○	○	○	○

Kommentar für Ethikkommission. Erfasstes Konzept: kognitive Komponente Naturverbundenheit. Standardisierte Skala (Nature relatedness Scale Short; Zelenski & Murphy, 2009).

Inwiefern treffen die folgenden Aussagen auf Sie zu?

	stimme überhaupt nicht zu	stimme eher nicht zu	weder dafür noch dagegen	stimme etwas zu	stimme völlig zu
Mein idealer Urlaubsort wäre ein abgelegenes Wildnisgebiet.	○	○	○	○	○
Ich denke stets daran, welche Auswirkungen mein Verhalten auf die natürliche Umwelt hat.	○	○	○	○	○
Meine Verbindung zur Natur und der Umwelt ist Teil meiner Spiritualität.	○	○	○	○	○
Wo immer ich bin, nehme ich die Tierwelt wahr.	○	○	○	○	○
Meine Beziehung zur Natur ist ein wichtiger Teil dessen, wer ich bin.	○	○	○	○	○
Ich fühle mich sehr verbunden mit allen lebenden Dingen und der Erde.	○	○	○	○	○

*Kommentar für Ethikkommission. Erfasstes Konzept: Chronischer Stress. Standardisierte Skala (*Trierer Stress Inventar; Schulz, Schlotz, & Becker, 2004*).*

Wenn Sie nicht nur Ihre Arbeit, sondern Ihren gesamten Alltag betrachten: Wie häufig haben Sie in den letzten drei Monaten die folgenden Erfahrungen gemacht bzw. Situationen erlebt?

	nie	selten	manchmal	häufig	sehr häufig
Ich befürchte, dass etwas Unangenehmes passieren wird.	○	○	○	○	○
Ich versuche vergeblich, Anerkennung für gute Arbeit zu bekommen.	○	○	○	○	○
Manchmal habe ich zu viele Verpflichtungen zu erfüllen.	○	○	○	○	○
Manchmal kann ich negative Gedanken nicht unterdrücken.	○	○	○	○	○
Obwohl ich mein Bestes gebe, wird meine Arbeit nicht anerkannt.	○	○	○	○	○
Ich habe das Gefühl, dass alles, was ich tun muss, mir zu viel wird	○	○	○	○	○
Manchmal mache ich mir große Sorgen und kann es nicht aufhalten.	○	○	○	○	○
Manchmal erfülle ich nicht das, was von mir erwartet wird.	○	○	○	○	○
Manchmal fühle ich mich durch meine Verantwortung gegenüber anderen überfordert.	○	○	○	○	○
Manchmal wächst mir die Arbeit über den Kopf.	○	○	○	○	○
Ich habe Angst, meine Pflichten nicht erfüllen zu können.	○	○	○	○	○
Manchmal wachsen mir die Sorgen über den Kopf.	○	○	○	○	○

*Kommentar für Ethikkommission. Erfasstes Konzept: Lebenszufriedenheit. Standardisierte Skala (*Kurzskala Lebenszufriedenheit-1; Beierlein et al., 2014*).*

Nun geht es um Ihre allgemeine Lebenszufriedenheit.
Wie zufrieden sind Sie gegenwärtig, alles in allem, mit Ihrem Leben?

Überhaupt nicht zufrieden									völlig zufrieden
○	○	○	○	○	○	○	○	○	○

Kommentar für Ethikkommission. Erfasstes Konzept: Rumination. Standardisierte Skala (Perseverative Thinking Questionnaire; Ehring, et al., 2011).

Im Folgenden geht es darum, wie Sie *typischerweise im Alltag* über negative Erlebnisse oder Probleme nachdenken.

Bitte lesen Sie die folgenden Aussagen und geben Sie an, in welchem Ausmaß diese *typischerweise im Alltag* auf Sie zutreffen, wenn Sie über negative Erlebnisse oder Probleme nachdenken.

	nie	selten	manchmal	häufig	fast immer
Dieselben Gedanken gehen mir immer und immer wieder durch den Kopf.	○	○	○	○	○
Meine Gedanken drängen sich mir auf.	○	○	○	○	○
Ich kann nicht aufhören, darüber nachzudenken.	○	○	○	○	○
Ich denke an viele Probleme, ohne eines von ihnen zu lösen.	○	○	○	○	○
Wenn ich über meine Probleme nachdenke, kann ich gleichzeitig nichts anderes tun.	○	○	○	○	○
Meine Gedanken wiederholen sich.	○	○	○	○	○
Gedanken tauchen auf, ohne dass ich dies will.	○	○	○	○	○
Ich hänge an bestimmten Themen fest und kann mich nicht davon lösen.	○	○	○	○	○
Ich stelle mir immer wieder Fragen, ohne zu einer Antwort zu kommen.	○	○	○	○	○
Meine Gedanken verhindern, dass ich mich auf andere Dinge konzentrieren kann.	○	○	○	○	○
Ich denke die ganze Zeit über dasselbe Thema nach.	○	○	○	○	○
Meine Gedanken bringen mich nicht weiter.	○	○	○	○	○
Meine Gedanken nehmen meine volle Aufmerksamkeit in Anspruch.	○	○	○	○	○

Kommentar für Ethikkommission. Erfasstes Konzept: Gedankenfokus. Standardisierte Skala (Situaltional Self-Awareness Scale; Govern, & Marsch, 2001).

Bitte geben Sie im Hinblick auf die nachfolgenden Aussagen an, inwiefern diese *im Alltag* auf Sie zutreffen.

	stimme überhaupt nicht zu				stimme voll zu
Ich bin mir allen Dingen in meiner Umgebung sehr bewusst.	○	○	○	○	○
Ich bin mir meiner inneren Gefühle bewusst.	○	○	○	○	○
Ich bin damit beschäftigt, wie ich mich selbst präsentiere.	○	○	○	○	○
Ich bin selbstbewusst hinsichtlich meines Aussehens.	○	○	○	○	○
Ich bin mir dessen bewusst, was um mich herum passiert.	○	○	○	○	○
Ich reflektiere über mein Leben.	○	○	○	○	○
Ich bin damit beschäftigt, was andere Leute über mich denken.	○	○	○	○	○
Ich bin mir meiner innersten Gedanken bewusst.	○	○	○	○	○
Ich bin mir allen Objekten um mich herum bewusst.	○	○	○	○	○

Wie alt sind Sie?

Alter in Jahren ☐

Welchem Geschlecht ordnen Sie sich zu?

- ○ weiblich
- ○ männlich
- ○ anderes

Welches ist Ihr höchster Schulabschluss?

- ○ keiner
- ○ Primar-/ Read-/ Sekundar-/ Bezirksschule/ Untergymnasium
- ○ Berufslehre, Berufsschule, KV; Gewerbeschule
- ○ Maturitätsschule, Gymnasium, LehrerInnenseminar, Berufsmaturität
- ○ Höhere Fach-, oder Berufsausbildung, Kunstgewerbeschule
- ○ Fachhochschule (z.B. FHS, HTL, HWV) und Pädagogische Hochschule
- ○ Technische Hochschulen (ETH), Universität
- ○ Anderes

Wie sind Sie momentan tätig?

- ☐ Vollzeit erwerbstätig (mind. 37 h pro Woche)
- ☐ Teilzeit erwerbstätig (5- 37 h pro Woche)
- ☐ Teilzeit erwerbstätig (1-4 h pro Woche)
- ☐ Pensioniert/ in Rente (AHV, IV etc.)
- ☐ Hausfrau/ Hausmann
- ☐ Arbeitslos
- ☐ in Ausbildung/ Studium

Wenn Sie einmal in ein Restaurant <u>richtig</u> <u>gut</u> Essen gehen, wie viel geben Sie maximal pro Person – inklusive Getränke – aus? (bitte ungefähren Maximalbetrag angeben)

- ○ CHF ☐
- ○ Gehe nie in ein Restaurant
- ○ Werde immer eingeladen

Vielen Dank für Ihre Teilnahme!

Sie bekommen nun mündlich Informationen zum weiteren Vorgehen.

C8: T2 Fragebogen für online-Umfrage in WP2

C9: T3 Fragebogen für online-Umfrage in WP2

Liebe Teilnehmerin, lieber Teilnehmer,

herzlich willkommen und vielen Dank, dass Sie unsere Fragen ein letztes Mal beantworten!

Bitte lesen Sie die Fragen in aller Ruhe durch und geben Sie an, was Ihnen als erstes in den Sinn kommt.

Die Bearbeitung des Fragebogens wird etwa 10 Minuten in Anspruch nehmen.

Teilnahmebedingungen und Datenschutz

Die Teilnahme an dieser Studie ist freiwillig, die Ergebnisse der Studie werden für wissenschaftliche Zwecke verwendet. Es sind keine Risiken mit dieser Studie verbunden. Sie können die Studie jederzeit ohne Angaben von Gründen abbrechen. Hierdurch entstehen Ihnen keine Nachteile. Alle Daten werden streng vertraulich gehalten. Es sind keine Rückschlüsse auf Ihre Person möglich.

☐ Ich bestätige, dass ich die Teilnahmebedingungen gelesen und verstanden habe und möchte an der Studie teilnehmen.

Bitte geben Sie Ihren vierstelligen persönlichen ID-Code an. Achten Sie dabei auf Groß- und Kleinschreibung.

ID-Code

Kommentar für Ethikkommission. Erfasstes Konzept: Wohlbefinden. Standardisierte Skala (Positive and Negative Affect Schedule; Watson et al., 1988).

Nun möchten wir gerne von Ihnen wissen, wie Sie sich fühlen.

Die folgenden Wörter beschreiben unterschiedliche Gefühle und Empfindungen. Lesen Sie jedes Wort und tragen Sie dann in die Skala neben jedem Wort die Intensität ein. Sie haben die Möglichkeit, zwischen fünf Abstufungen zu wählen. Geben Sie bitte an, wie Sie sich in den letzten 10 Tagen gefühlt haben.

	gar nicht	ein bisschen	einigermaßen	erheblich	äußerst
aktiv	○	○	○	○	○
bekümmert	○	○	○	○	○
interessiert	○	○	○	○	○
freudig erregt	○	○	○	○	○
verärgert	○	○	○	○	○
stark	○	○	○	○	○
schuldig	○	○	○	○	○
erschrocken	○	○	○	○	○
feindselig	○	○	○	○	○
angeregt	○	○	○	○	○
stolz	○	○	○	○	○
gereizt	○	○	○	○	○
begeistert	○	○	○	○	○
beschämt	○	○	○	○	○
wach	○	○	○	○	○
nervös	○	○	○	○	○
entschlossen	○	○	○	○	○
aufmerksam	○	○	○	○	○
durcheinander	○	○	○	○	○
ängstlich	○	○	○	○	○

Kommentar für Ethikkommission. Erfasstes Konzept: akustischer Eindruck. Standardisierte Skala (Soundscape; ISO/TS 12913-2:2018, 2018).

Wenn Sie an die Geräuschsituation während der Spaziergänge in den letzten 10 Tagen denken, in welchem Ausmass haben Sie während der Spaziergänge die folgenden vier Geräuscharten gehört?

	überhaupt nicht	ein bisschen	moderat	sehr	komplett dominierend
Verkehrslärm (z.B. Autos, Busse, Züge, Flugzeuge)	○	○	○	○	○
Andere Geräusche (z.B. Sirenen, Bauarbeiten, Industrie, Ladung von Waren)	○	○	○	○	○
Geräusche von Menschen (z.B. Gespräche, Lachen, spielende Kinder, Schritte)	○	○	○	○	○
Naturgeräusche (z.B. Vogelzwitschern, Wasser, Wind)	○	○	○	○	○

Wenn Sie an die Geräuschsituation während der Spaziergänge in den letzten 10 Tagen denken, welche Begriffe beschreiben den Höreindruck am zutreffendsten?

	trifft nicht zu				trifft zu
angenehm	○	○	○	○	○
chaotisch	○	○	○	○	○
lebendig	○	○	○	○	○
ereignisarm	○	○	○	○	○
ruhig	○	○	○	○	○
störend	○	○	○	○	○
ereignisreich	○	○	○	○	○
eintönig	○	○	○	○	○
laut	○	○	○	○	○

Wie würden Sie die Geräuschumgebung während der Spaziergänge in den letzten 10 Tagen im Allgemeinen beschreiben?

	sehr gut	gut	weder gut noch schlecht	schlecht	sehr schlecht
Wie würden Sie die Geräuschumgebung während des Spaziergangs im Allgemeinen beschreiben?	○	○	○	○	○

Wie stark haben Sie sich während der Spaziergänge in den letzten 10 Tagen durch Lärm der folgenden Quellen gestört oder belästigt gefühlt?

	Überhaupt nicht	etwas	mittelmäßig	stark	äußerst
Straßenverkehr	○	○	○	○	○
Eisenbahnverkehr	○	○	○	○	○
Flugverkehr	○	○	○	○	○
Freizeitgeräusche	○	○	○	○	○
Naturgeräusche	○	○	○	○	○

Nun geht es um das Ausmass an Vegetation in der Umgebung, in der Sie in den letzten 10 Tagen spazieren gegangen sind.

	sehr wenig									sehr viel
Wie viel Vegetation gab es in der Umgebung?	○	○	○	○	○	○	○	○	○	○

Kommentar für Ethikkommission. Die Bilder entsprechen den potenziellen Settings und wurden selbst aufgenommen.

Denken Sie an die Spaziergänge in den letzten 10 Tagen. Welches der folgenden Bilder ähnelt der Vegetation in der Umgebung, in der Sie in den letzten 10 Tagen spazieren gegangen sind am meisten?

○ 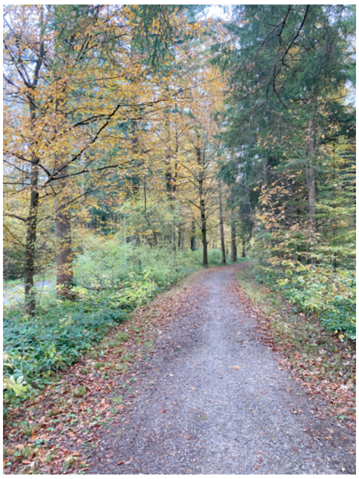

○ 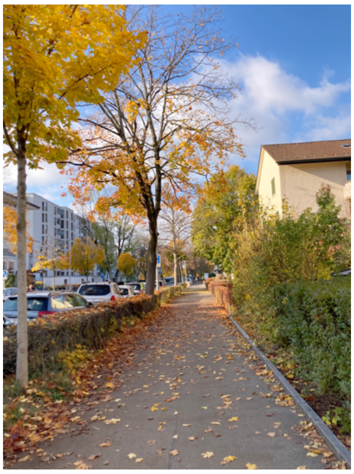

○ 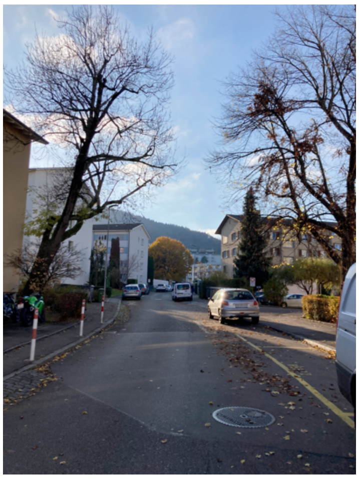

○ 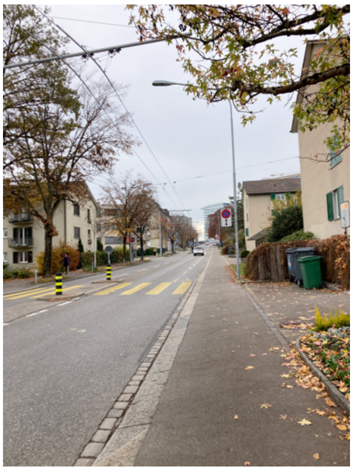

○ 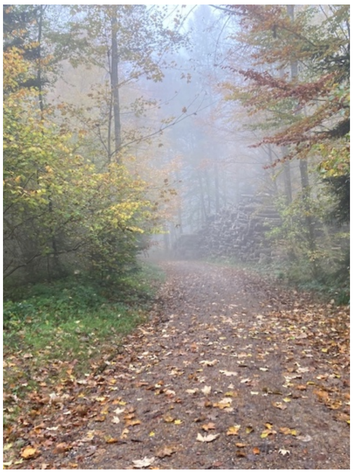

○ 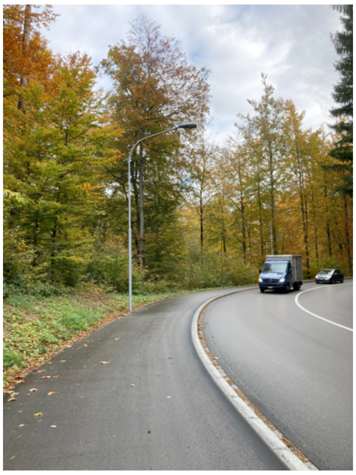

Kommentar für Ethikkommission. Erfasstes Konzept: Restorative Qualität der Umgebung. Standardisierte Skala (Perceived Restorativeness Scale-11; Pasini, et al., 2014).

Wir interessieren uns dafür, wie Sie die Umgebung, in der Sie in den letzten 10 Tagen spazieren gegangen sind wahrgenommen haben.

Inwiefern treffen die folgenden Aussagen auf Ihre Erfahrungen bei den Spaziergängen zu?

	überhaupt nicht						komplett
Orte wie dieser sind faszinierend.	○	○	○	○	○	○	○
An Orten wie diesem wird meine Aufmerksamkeit auf viele interessante Dinge gelenkt.	○	○	○	○	○	○	○
An Orten wie diesem ist es schwer, sich zu langweilen.	○	○	○	○	○	○	○
Orte wie dieser sind eine Zuflucht vor Ärgernissen.	○	○	○	○	○	○	○
Um von Dingen wegzukommen, die normalerweise meine Aufmerksamkeit fordern, gehe ich gerne an solche Orte.	○	○	○	○	○	○	○
Um aufzuhören, über die Dinge nachzudenken, die ich erledigen muss, gehe ich gerne an Orte wie diesen.	○	○	○	○	○	○	○
Es gibt eine klare Ordnung in der physischen Anordnung von Orten wie diesem.	○	○	○	○	○	○	○
An Orten wie diesem ist es einfach zu sehen, wie die Dinge organisiert sind.	○	○	○	○	○	○	○
An Orten wie diesem scheint alles seinen richtigen Platz zu haben.	○	○	○	○	○	○	○
Dieser Ort ist groß genug, um Erkundungen in viele Richtungen zu ermöglichen	○	○	○	○	○	○	○
An solchen Orten gibt es wenige Grenzen, die meine Bewegungsmöglichkeiten einschränken.	○	○	○	○	○	○	○

Kommentar für Ethikkommission. Erfasstes Konzept: Erholung. Standardisierte Skala (Restoration Outcome Scale; Korpela, M.; Ylén, M.; Tyrväinen, L. & Silvennoinen, H., 2008).

Denken Sie an die Umgebungen, durch die Sie während der letzten 10 Tage spaziert sind.

Inwieweit hat die Umgebung zu Folgendem beigetragen?

	trifft überhaupt nicht zu	trifft nicht zu	trifft eher nicht zu	trifft zu	trifft voll zu
Ich fühle mich ruhiger, nachdem ich dort war.	○	○	○	○	○
Ich fühle mich erholt und entspannt, nachdem ich dort war.	○	○	○	○	○
Ich kann in dieser Umgebung Energie tanken für meine täglichen Aufgaben.	○	○	○	○	○
Ich kann mich in dieser Umgebung besser konzentrieren und fühle mich wacher.	○	○	○	○	○
Ich kann in dieser Umgebung meine Alltagssorgen vergessen.	○	○	○	○	○
Ich kann in dieser Umgebung meine Gedanken ordnen und frei machen.	○	○	○	○	○

*Kommentar für Ethikkommission. Erfasstes Konzept: Trait-Achtsamkeit. Standardisierte Skala (*Kentucky Inventory for Mindfulness Skills Short; Höfling et al., 2011*).*

Schätzen Sie bitte jede der folgenden Aussagen mit Hilfe der bereitgestellten Skala ein. Wählen Sie dabei die Ziffer aus, die im Allgemeinen am besten/ehesten auf Sie zutrifft. Bitte antworten Sie spontan, ohne lange darüber nachzudenken, und so, wie Sie die Dinge tatsächlich erleben und nicht, wie Sie sie gerne erleben würden.

	trifft nie oder sehr selten zu	trifft selten zu	trifft manchmal zu	trifft oft zu	trifft sehr oft oder immer zu
Ich kann meine Gefühle gut in Worte fassen.	○	○	○	○	○
Ich kritisiere mich dafür, irrationale oder unangebrachte Gefühle zu haben.	○	○	○	○	○
Ich konzentriere mich nur auf das, was ich gerade tue und auf nichts anderes.	○	○	○	○	○
Wenn ich gehe, dann nehme ich ganz bewusst wahr, wie sich die Bewegungen meines Körpers anfühlen.	○	○	○	○	○
Wenn ich dusche oder bade, bin ich mir dieses Gefühls des Wassers auf meinem Körper bewusst.	○	○	○	○	○
Es fällt mir schwer, das, was ich denke, in Worte zu fassen.	○	○	○	○	○
Ich glaube, dass einige meiner Gedanken unnormal sind und dass ich nicht so denken sollte.	○	○	○	○	○
Ich habe Schwierigkeiten, die richtigen Worte zu finden, um meine Gefühle auszudrücken.	○	○	○	○	○
Wenn ich etwas tue, dann bin ich davon völlig eingenommen und denke an nichts anderes mehr.	○	○	○	○	○
Ich urteile darüber, ob meine Gedanken gut oder schlecht sind.	○	○	○	○	○
Ich achte auf meine Empfindungen, wie zum Beispiel Wind in meinem Haar oder Sonnenschein auf meinem Gesicht.	○	○	○	○	○
Körperliche Empfindungen sind für mich schwer zu beschreiben, weil mir die richtigen Worte dazu fehlen.	○	○	○	○	○
Ich achte auf Geräusche, wie beispielsweise das Ticken von Uhren, Vogelzwitschern oder das Geräusch vorüberfahrender Autos.	○	○	○	○	○
Sogar wenn ich schrecklich verärgert bin, kann ich das in Worte fassen.	○	○	○	○	○
Ich sage mir, dass ich nicht so denken sollte, wie ich denke.	○	○	○	○	○
Ich nehme Gerüche und Düfte der Dinge wahr.	○	○	○	○	○
Ich neige dazu mehrere Dinge gleichzeitig zu tun, anstatt mich nur auf eine Sache zu konzentrieren.	○	○	○	○	○
Ich denke, dass manche meiner Gefühle schlecht oder unangebracht sind und dass ich sie nicht haben sollte.	○	○	○	○	○
Ich bemerke visuelle Elemente sowohl in der Kunst als auch in der Natur, zum Beispiel Farben, Formen, Struktur oder Muster aus Licht und Schatten.	○	○	○	○	○
Wenn ich etwas tue, werde ich so davon eingenommen, dass meine ganze Aufmerksamkeit darauf gerichtet ist.	○	○	○	○	○

Kommentar für Ethikkommission. Erfasstes Konzept: emotionale Komponente Naturverbundenheit*. Standardisierte Skala (*Love and Care for Nature Scale; Perkins, 2010*).*

Inwiefern treffen die folgenden Aussagen für Sie zu?

	stimme überhaupt nicht zu						stimme voll und ganz zu
Ich empfinde eine tiefe Liebe zur Natur.	○	○	○	○	○	○	○
Der Schutz der Natur und ihr Wohlergehen ist mir wichtig.	○	○	○	○	○	○	○
Ich empfinde oft ein Gefühl von Ehrfurcht und Erstaunen, wenn ich in unberührter Natur bin.	○	○	○	○	○	○	○
Ich empfinde oft ein starkes Bedürfnis der Fürsorge gegenüber der natürlichen Umgebung.	○	○	○	○	○	○	○
Ich fühle mich der Natur oft emotional nahe.	○	○	○	○	○	○	○

Kommentar für Ethikkommission. Erfasstes Konzept: kognitive Komponente Naturverbundenheit. Standardisierte Skala (Nature relatedness Scale Short; Zelenski & Murphy, 2009).

Inwiefern treffen die folgenden Aussagen für Sie zu?

	stimme überhaupt nicht zu	stimme eher nicht zu	weder dafür noch dagegen	stimme etwas zu	stimme völlig zu
Mein idealer Urlaubsort wäre ein abgelegenes Wildnisgebiet.	○	○	○	○	○
Ich denke stets daran, welche Auswirkungen mein Verhalten auf die natürliche Umwelt hat.	○	○	○	○	○
Meine Verbindung zur Natur und der Umwelt ist Teil meiner Spiritualität.	○	○	○	○	○
Wo immer ich bin, nehme ich die Tierwelt wahr.	○	○	○	○	○
Meine Beziehung zur Natur ist ein wichtiger Teil dessen, wer ich bin.	○	○	○	○	○
Ich fühle mich sehr verbunden mit allen lebenden Dingen und der Erde.	○	○	○	○	○

Kommentar für Ethikkommission. Erfasstes Konzept: Rumination. Standardisierte Skala (Perseverative Thinking Questionnaire; Ehring, et al., 2011).

Im Folgenden geht es darum, wie Sie *in den letzten 10 Tagen* über negative Erlebnisse oder Probleme nachgedacht haben.

Bitte lesen Sie die folgenden Aussagen und geben Sie an, in welchem Ausmaß diese auf Sie zugetroffen haben, wenn Sie über negative Erlebnisse oder Probleme nachgedacht haben.

	nie	selten	manchmal	häufig	fast immer
Dieselben Gedanken gehen mir immer und immer wieder durch den Kopf.	○	○	○	○	○
Meine Gedanken drängen sich mir auf.	○	○	○	○	○
Ich kann nicht aufhören, darüber nachzudenken.	○	○	○	○	○
Ich denke an viele Probleme, ohne eines von ihnen zu lösen.	○	○	○	○	○
Wenn ich über meine Probleme nachdenke, kann ich gleichzeitig nichts anderes tun.	○	○	○	○	○
Meine Gedanken wiederholen sich.	○	○	○	○	○
Gedanken tauchen auf, ohne dass ich dies will.	○	○	○	○	○
Ich hänge an bestimmten Themen fest und kann mich nicht davon lösen.	○	○	○	○	○
Ich stelle mir immer wieder Fragen, ohne zu einer Antwort zu kommen.	○	○	○	○	○
Meine Gedanken verhindern, dass ich mich auf andere Dinge konzentrieren kann.	○	○	○	○	○
Ich denke die ganze Zeit über dasselbe Thema nach.	○	○	○	○	○
Meine Gedanken bringen mich nicht weiter.	○	○	○	○	○
Meine Gedanken nehmen meine volle Aufmerksamkeit in Anspruch.	○	○	○	○	○

Kommentar für Ethikkommission. Erfasstes Konzept: Gedankenfokus. Standardisierte Skala (Situaltional Self-Awareness Scale; Govern, & Marsch, 2001).

Bitte geben Sie im Hinblick auf die nachfolgenden Aussagen an, inwiefern diese *während der letzten 10 Tage* zugetroffen haben.

	stimme überhaupt nicht zu				stimme voll zu
Ich war mir allen Dingen in meiner Umgebung sehr bewusst.	○	○	○	○	○
Ich war mir meiner inneren Gefühle bewusst.	○	○	○	○	○
Ich war damit beschäftigt, wie ich mich selbst präsentiere.	○	○	○	○	○
Ich war selbstbewusst hinsichtlich meines Aussehens.	○	○	○	○	○
Ich bin mir dessen bewusst, was um mich herum passiert.	○	○	○	○	○
Ich habe über mein Leben reflektiert.	○	○	○	○	○
Ich war damit beschäftigt, was andere Leute über mich denken.	○	○	○	○	○
Ich war mir meiner innersten Gedanken bewusst.	○	○	○	○	○
Ich war mit allen Objekten um mich herum bewusst.	○	○	○	○	○

*Kommentar für Ethikkommission. Erfasstes Konzept: Lebenszufriedenheit. Standardisierte Skala (*Kurzskala Lebenszufriedenheit-1; Beierlein et al., 2014*).*

Nun geht es um Ihre allgemeine Lebenszufriedenheit.
Wie zufrieden sind Sie gegenwärtig, alles in allem, mit Ihrem Leben?

Überhaupt nicht zufrieden									völlig zufrieden
○	○	○	○	○	○	○	○	○	○

Wie oft haben Sie die Achtsamkeitsübung in den letzten 10 Tagen gemacht?

[Bitte auswählen]

Hat die Übung deinen Aufenthalt in der Natur verändert? Wenn ja wie?

Vielen Dank für Ihre Teilnahme!

Wir möchten uns ganz herzlich für Ihre Mithilfe bedanken.

Ihre Antworten wurden gespeichert, Sie können das Browser-Fenster nun schließen.

C10: Achtsamkeitsintervention

Einführung (*nachdem Teilnehmende den Fragebogen ausgefüllt haben und während Teilnehmende noch sitzen)*

- Wir werden ca. eine halbe Stunde Spazierengehen. Der Spaziergang findet im Schweigen statt, bitte unterhaltet euch nicht und schaltet bitte eure Handys aus oder auf lautlos.
- Passt eure Geschwindigkeit während des Spaziergangs meiner Geschwindigkeit an. Bleibt während des Spaziergangs zusammen und hinter mir, keiner sollte mich überholen.
- Wir werden zwischendurch einige Male kurz innehalten und ihr werdet Impulse bekommen, auf welche Elemente von dem, was ihr erlebt ihr in den nächsten Minuten achten könnt. Achtet darauf, dass ihr nicht zu viel Abstand zur Gruppe habt, damit ihr die Ansagen zwischendurch hören könnt.
- Während des Spaziergangs seid ihr eingeladen euch zu erlauben, eure Aufmerksamkeit auf unterschiedlichen momentanen Erfahrungen ruhen zu lassen, z.B. Dinge die ihr seht oder hört. Sei einfach offen für die Erfahrung und mache das Ganze mühelos.
- Mach das nicht als Übung. Es ist eine Einladung das zu erleben was auch immer gerade erfährst, an diesem Ort und in diesem Moment. Ihr werdet nach dem Spaziergang nicht abgefragt was ihr gesehen oder gehört habt.
- Wir werden die Übung jetzt starten. Bitte steht auf.

Während man in der Gruppe zusammensteht

- Halte deine Augen sanft offen. Beobachte was du siehst. Es geht nicht darum zu versuchen etwas Bestimmtes zu sehen. Schau einfach, ob du deinen Blick entspannt halten kannst und lass ihn sich frei bewegen wohin er möchte, während wir die ersten Minuten Gehen. Folgt mir einfach.

5 Minuten laufen

Während man in der Gruppe zusammensteht. Wenn Personen nicht nah genug stehen werden sie rangewunken.

Der Körper

- Erlaube dir mit deinen Fußsohlen in Verbindung zu gehen und nehme wahr, wie sie den Boden berühren.
- Sei neugierig auf das Gefühl, wie deine Füße mit jedem Schritt den Boden berühren.
- Breite deine Aufmerksamkeit auf deine Beine aus.
- Lass dich deine Beine spüren während sie sich bewegen und schau, was du in deinen Hüften, Knien uws. Wahrnimmst.
- Wie der Boden sich unter deinen Füßen anfühlt: angenehm, unangenehm….
- Lass deine Aufmerksamkeit sich auf den ganzen Körper ausbreiten.
- Was nimmst du sonst noch wahr in deinem Körper? Z.B. den Wind oder die Sonne auf deiner Haut?

5 Minuten laufen

Während man in der Gruppe zusammensteht. Wenn Personen nicht nah genug stehen werden sie rangewunken.

Geräusche

- Öffne dich für die Geräusche um dich herum.
- Weniger dafür, was die Geräusche macht, sondern mehr für die Geräusche selbst.
- Lass die Geräusche zu dir kommen wie sie kommen. Du brauchst nicht nach ihnen zu lauschen.
- Die schwachen Geräusche und die starken. Die nahen und die weiter weg. Die plötzlichen und die, die kontinuierlich da sind.

- Erlaube dir sie in dir aufzunehmen, so wie sie bei dir in jedem Moment bei dir ankommen.
- Lass dich erkunden wie die Geräusche sich um dich herum verändern, und vielleicht auch wie deine Erfahrung der Geräusche sich verändert.
- Heiße alle deine Gedanken und anderen Reaktionen auf die Geräusche willkommen und nehme sie in dir auf wenn sie kommen.
- Gedanken und Reaktionen entstehen als natürlicher Teil des Gehens und Hörens.
- Lass die Gedanken und Reaktionen kommen und gehen wenn sie kommen und gehen und lass sie genauso flüchtig sein wie die Geräusche selbst.

5 Minuten laufen

Während man in der Gruppe zusammensteht. Wenn Personen nicht nah genug stehen werden sie rangewunken.

Das Sehen
- Ich lade dich ein, dich in den nächsten Minuten für das zu öffnen was du siehst. Wo will deine Blick hinschweifen? Wo geht er hin und was zieht in an? Lass den Blick frei wandern.
- Lass dich auf die Farben ein, den Boden, die Bäume, die Bewegung der Blätter im Wind.
- Nimm die Gefühle war die dabei aufkommen was du siehst. Es ist okay wenn es angenehm ist, aber es ist auch okay wenn es nicht angenehm ist.
- Heise deine Gedanken und Reaktionen willkommen, wenn sie kommen und gehen. Erlaube dir dich damit wohlzufühlen. Und lasse das was du siehst und die Gefühle verblassen, wenn sie es tun.

5 Minuten laufen

Während man in der Gruppe zusammensteht. Wenn Personen nicht nah genug stehen werden sie rangewunken.

- Jetzt genieße 10 Min. Gehen in Stille.
- Öffne dich für die Erfahrung des gegenwärtigen Moments.
- Schau ob du diese offene Präsenz mitnehmen kannst in den nächsten 10 Minuten.
- Nimm wahr was für dich wichtig ist.
- Was fühlst du? Was hörst du? Riechst du etwas?
- Nimm wahr ob deine Wahrnehmung sich mit dieser offenen Präsenz verändert.

Während des Laufens
- Wir werden noch 1-2 Minuten Gehen und den Spaziergang dann beenden.

Wenn die Gruppe angekommen ist

- Hier sind wir wieder angekommen.
- Wir beenden den Spaziergang indem wir für ein paar Momente stehen, du kannst deine Augen schließen, wenn du willst. Nimm wahr ob es einen Unterschied gibt zwischen jetzt und als wir den Spaziergang begonnen haben.
- Nimm wahr was du fühlst.
- Danke für deine Teilnahme.

C11: Mustervertrag Datenverarbeitungsvertrag im Auftrag nach EU-Datenschutzgrundverordnung (DSGVO)

Vereinbarung über die Verarbeitung personenbezogener Daten im Auftrag gemäß Art. 28, 29 DSGVO

zwischen

Eidg. Forschungsanstalt WSL
Zürcherstr. 111
CH-8903 Birmensdorf

— Verantwortlicher, nachfolgend auch „**Auftraggeber**“ genannt —

und

xy

— Auftragsverarbeiter, nachfolgend auch „**Auftragnehmer**“ genannt —

Beide Vertragsparteien werden in nachstehender Vereinbarung auch einzeln als **Partei** und gemeinsam als **Parteien** gezeichnet.

Präambel

Zwischen Auftraggeber und Auftragnehmer wurde ein Hauptvertrag geschlossen, welcher die Nutzung der Dienstleistung des Auftragnehmers als Software-as-a-Service (SaaS, auch Cloud-Service) regelt. Gegenstand der Dienstleistung sind Onlineumfrageprojekte im Rahmen und auf Grundlage der hierfür vom Auftragnehmer zur Verfügung gestellten Software, wobei der Hauptvertrag eine oder mehrere Umfragen (Befragungsprojekte) umfassen kann.

Der Hauptvertrag kam durch Registrierung eines Benutzerkontos auf Basis der Allgemeinen Geschäftsbedingungen (AGB) der SoSci Survey GmbH zustande. Der Hauptvertrag wurde auf unbestimmte Zeit geschlossen. Die vertraglichen Vereinbarungen des Hauptvertrags ergeben sich aus den AGB.

Der Hauptvertrag sieht für die Vertragserfüllung notwendig unter anderem eine Verarbeitung von Daten durch den Auftragnehmer im Auftrag des Auftraggebers vor. Der Auftraggeber beauftragt den Auftragnehmer mit der Auftragsverarbeitung im Zusammenhang mit dem dieser Vereinbarung zugrundeliegenden Hauptvertrags, wie vorab beschrieben. Die im Zusammenhang mit dem Hauptvertrag erhobenen personenbezogenen Daten sind nach Art, Zweck und Umfang in Anlage 1 zu dieser Vereinbarung näher beschrieben.

Folgende Vereinbarung erläutert die datenschutzrechtlichen Verpflichtungen der Parteien, die sich aus der Beauftragung des Auftragnehmers durch den Hauptvertrag ergeben. Diese Vereinbarung zur Auftragsverarbeitung (im Folgenden kurz: „AVV“) ergänzt den Hauptvertrag/Haupttätigkeit in datenschutzrechtlicher Hinsicht. Diese AVV findet Anwendung auf sämtliche Tätigkeiten, bei denen der Auftragnehmer personenbezogene Daten des Auftraggebers verarbeitet. Begriffsdefinitionen richten sich nach der DSGVO und dem Bundesdatenschutzgesetz in seiner neuen Fassung oder alternativ den Landesdatenschutzgesetzen, sofern deren Anwendbarkeit eröffnet ist.

Die DSGVO gilt ab dem 25. Mai 2018. Sofern die Parteien bereits eine Vereinbarung über die Auftragsdatenverarbeitung (ADV) geschlossen haben, die zum Stichtag 25. Mai 2018 grundsätzlich noch Laufzeit hat, ersetzt dieser Vertrag die ADV ab Geltung der DSGVO.

Dies vorausgeschickt vereinbaren die Parteien wie folgt:

1. Anwendungsbereich, Auftragsgegenstand (Art. 28 Abs. 1 DSGVO)
    1. Im Rahmen der Leistungserbringung nach dem, dieser AVV zugrundeliegenden Hauptleistung, ist es erforderlich, dass der Auftragnehmer Zugriff auf personenbezogene Daten des Auftraggebers, seiner insoweit eingebundenen Angestellten, Umfrageteilnehmer oder sonstiger betroffener Dritter erhält oder bei Inanspruchnahme der Hauptleistung durch Nutzung der Software des Auftragnehmers personenbezogene Daten erhält. Diese Daten werden nachfolgend einheitlich (personenbezogene) Daten genannt. Im Zuge der Durchführung der Haupttätigkeit/Hauptvertrag wird der Auftragnehmer vom Auftraggeber mit der Verarbeitung der vertragsgegenständlichen Daten im Rahmen der angebotenen Softwarelösung beauftragt. Diese AVV konkretisiert die datenschutzrechtlichen Rechte und Pflichten der Vertragsparteien bei der Durchführung der Hauptleistung.
    2. Gegenstand der Tätigkeit des Auftragnehmers ist nicht die originäre Verarbeitung von personenbezogenen Daten. Im Zuge der Leistungserbringung des Auftragnehmers im Rahmen der Hauptleistung kann ein Zugriff auf personenbezogene Daten jedoch nicht ausgeschlossen werden.
    3. Alle Begrifflichkeiten dieser AVV werden im Sinn und im Verständnis nach der europäischen Datenschutzgrundverordnung (Verordnung (EU) 2016/679 des Europäischen Parlaments und des Rates, im Folgenden: kurz DSGVO) verwendet, wobei insbesondere
        - „personenbezogene Daten“ gemäß Art 4 Ziffer 1 DSGVO alle Informationen, die sich auf eine identifizierte oder identifizierbare natürliche Person (im Folgenden „betroffene Person“) beziehen bedeutet. Als identifizierbar wird eine natürliche Person angesehen, die direkt oder indirekt, insbesondere mittels Zuordnung zu einer Kennung wie einem Namen, zu einer Kennnummer, zu Standortdaten, zu einer Online-Kennung oder zu einem oder mehreren besonderen Merkmalen identifiziert werden kann, die Ausdruck der physischen, physiologischen, genetischen, psychischen, wirtschaftlichen, kulturellen oder sozialen Identität dieser natürlichen Person sind.
        - „Verarbeitung“ gemäß Art 4 Ziffer 2 DSGVO jeden mit oder ohne Hilfe automatisierter Verfahren ausgeführten Vorgang oder jede solche Vorgangsreihe im Zusammenhang mit personenbezogenen Daten wie das Erheben, das Erfassen, die Organisation, das Ordnen, die Speicherung, die Anpassung oder Veränderung, das Auslesen, das Abfragen, die Verwendung, die Offenlegung durch übermittlung, Verbreitung oder eine andere Form der Bereitstellung, den Abgleich oder die Verknüpfung, die Einschränkung, das Löschen oder die Vernichtung bedeutet.
    4. Die Vertragsparteien ergänzen und konkretisieren mit der gegenständlichen AVV die gegenseitigen Pflichten im generellen Umgang mit den vom Auftraggeber zur Verfügung gestellten Daten oder den für ihn erhobenen Daten. Im Falle eines Widerspruchs zwischen den Bestimmungen dieser Vereinbarung und denjenigen der Hauptleistung gehen die Bestimmungen dieser AVV denjenigen der Hauptleistungsvereinbarung vor.
    5. Der Geltungsbereich dieses AVV ist auf die Verarbeitung personenbezogener Daten im Rahmen des Befragungsprojekts RESTORE_t2acht beschränkt. Gegenstand ist, wie in Anlage 1 konkretisiert, im Wesentlichen die Verarbeitung von Adressdaten zum Zweck der elektronischen Kontaktaufnahme (Versand von Serienmails oder SMS) im Rahmen der oben genannten Onlinebefragung.
2. Bestimmung des Auftragsgegenstandes, Laufzeit
    1. Umfang, Art und Zweck der Aufgaben des Auftragnehmers zur Verarbeitung von Daten in Bezug auf den Auftragsgegenstand ergeben sich aus dem Hauptvertrag. Die Verarbeitung der Daten findet ausschließlich im Gebiet der

Bundesrepublik Deutschland, in einem Mitgliedsstaat der Europäischen Union oder in einem anderen Vertragsstaat des Abkommens über den Europäischen Wirtschaftsraum statt. Jede Verlagerung in ein Drittland bedarf der vorherigen schriftlichen Zustimmung des Auftraggebers und darf nur erfolgen, wenn die besonderen Voraussetzungen der einschlägigen datenschutzrechtlichen Vorschriften, insbesondere die Vorschriften zu übermittlungen personenbezogener Daten an Drittländer oder an internationale Organisationen, erfüllt sind.

2. Die vertragsgegenständlichen Daten werden vom Auftragnehmer ausschließlich im Auftrag und nach Weisungen des Auftraggebers im Sinne von Art. 28, 29 DSGVO (Auftragsverarbeitung) verarbeitet. Verantwortlicher im datenschutzrechtlichen Sinn bleibt der Auftraggeber und dieser trägt somit die Verantwortung für die Rechtmäßigkeit der auftragsgemäßen Verarbeitung der vertragsgegenständlichen Daten. Der Auftragnehmer wird diese Daten daher nur auf Weisung des Auftraggebers verarbeiten, wie nachstehend in Ziffer 5 weiter festgelegt. Die Verantwortlichkeit des Auftraggebers bezieht sich insbesondere darauf, dass die vertrags- und weisungsgemäße Datenverarbeitung rechtmäßig ist, die Grundsätze für die Verarbeitung personenbezogener Daten eingehalten werden und deren Einhaltung nachgewiesen werden kann.
3. Die Art der betroffenen vertragsgegenständlichen Daten und die Kategorien der durch die Verarbeitung betroffenen Personen sind in **Anlage 1** abschließend normiert.
4. Die Laufzeit dieser Vereinbarung richtet sich nach der Laufzeit des Befragungsprojekts, für welches dieser AVV gemäß Ziffer 1.5 Gültigkeit besitzt. Der Vertrag beginnt mit der Unterzeichnung der vorliegenden Vereinbarung, nicht jedoch vor Wirksamkeit der zugrunde liegenden Hauptleistungsvereinbarung. Ziffer 14.1 bleibt hiervon unberührt.
5. Die Parteien sind sich bewusst, dass die Auftragsverarbeitung nicht ohne wirksame AVV erfolgen darf, sodass die Auftragsverarbeitung im Falle der Beendigung der gegenständlichen AVV bis zum Abschluss einer neuen AVV über die Verarbeitung personenbezogener Daten im Auftrag trotz bestehender Hauptleistungsvereinbarung nicht erfolgen darf. Spiegelbildlich ist Gegenstand dieser AVV nicht die originäre Nutzung oder Verarbeitung von personenbezogenen Daten durch den Auftragnehmer, dennoch kann im Zuge der Hauptleistungserbringung ein Zugriff auf personenbezogene Daten nicht ausgeschlossen werden. Erfolgt damit keine zu erbringende Hauptleistung während der Laufzeit dieser AVV, berechtigt diese AVV allein den Auftragnehmer ebenfalls nicht zur Verarbeitung personenbezogener Daten im Auftrag. Hierfür bedarf es einer zugrundeliegenden Hauptleistung.

3. Technische und organisatorische Maßnahmen (TOM)
    1. Der Auftragnehmer gestaltet in seinem Verantwortungsbereich die innerbetriebliche Organisation so, dass sie den Anforderungen des Datenschutzes gerecht wird. Er trifft dabei technische und organisatorische Maßnahmen zur angemessenen Sicherung der Daten vor Missbrauch und Verlust, die den Anforderungen der DSGVO entsprechen. Soweit es den Parteien erforderlich erscheint, kann dem Auftraggeber ein Verzeichnis der technisch-organisatorischen Maßnahmen mit Vertragsschluss übergeben werden.
    2. Die technischen und organisatorischen Maßnahmen unterliegen dem technischen Fortschritt und der Weiterentwicklung. Der Auftragnehmer ist verpflichtet, die technischen und organisatorischen Maßnahmen dem Stand der Technik anzupassen. Insoweit ist es dem Auftragnehmer gestattet, alternative adäquate Maßnahmen umzusetzen. Dabei darf das Sicherheitsniveau der festgelegten Maßnahmen nicht unterschritten werden. Bei geringfügigen Änderungen an den technischen und organisatorischen Maßnahmen (z.B. Ersatz der Schließanlage durch eine neue, jedoch gleichwertige) ist die Änderung lediglich zu dokumentieren. Bei wesentlichen Änderungen (z.B. grundlegende Änderung von Verschlüsselungssystemen) ist vorab die schriftliche Zustimmung des Auftraggebers einzuholen. Der Auftragnehmer hat

auf Anforderung des Auftraggebers an der Erstellung der Verarbeitungsverzeichnisse des Auftragsgebers, die die Auftragsverarbeitung nach dieser Vereinbarung betreffen, mitzuwirken, insbesondere die hierfür erforderlichen Angaben des Auftraggebers zur Verfügung zu stellen.

3. Solange das angemessene und vereinbarte Schutzniveau nicht unterschritten wird und dem Stand der Technik entspricht, hat der Auftraggeber seine Zustimmung zu erteilen, außer wichtige Gründe stehen der Einführung entgegen. Geringfügige Änderungen werden nur als Ergänzung zu den technisch-organisatorischen Maßnahmen vom Auftragnehmer dokumentiert. Alle Vorabversionen der technisch-organisatorischen Maßnahmen werden vom Auftragnehmer zum Nachweis geringfügiger Abweichungen dokumentiert
4. Bei der Verarbeitung personenbezogener Daten ist der Auftragnehmer verpflichtet, die datenschutzrechtlichen Grundsätze einzuhalten sowie die Sicherheit herzustellen, die zum Schutz personenbezogener Daten erforderlich ist. Insgesamt handelt es sich bei allen zu treffenden Maßnahmen um Maßnahmen der Datensicherheit und zur Gewährleistung eines dem Risiko angemessenen Schutzniveaus hinsichtlich der Vertraulichkeit, der Integrität, der Verfügbarkeit sowie der Belastbarkeit der Systeme (Art. 32 Abs. 1 lit. b DSGVO). Dabei sind der Stand der Technik, die Implementierungskosten und die Art, der Umfang und die Zwecke der Verarbeitung sowie die unterschiedliche Eintrittswahrscheinlichkeit und Schwere des Risikos für die Rechte und Freiheiten natürlicher Personen zu berücksichtigen.

4. Qualitätsmanagement, Verpflichtungen des Auftragnehmers

Ergänzend zur Einhaltung der Regelungen dieser Vereinbarung hat der Auftragnehmer weitere datenschutzrechtliche Pflichten. Er gewährleistet insbesondere die Einhaltung folgender Vorgaben:

1. Soweit gesetzlich vorgeschrieben, die Bestellung eines Datenschutzbeauftragten (in schriftlicher Form), der seine Tätigkeit nach Maßgabe der datenschutzrechtlichen Vorschriften ausüben kann. Eine Neubesetzung des Datenschutzbeauftragten und/oder dessen Kontaktdaten während der Dauer dieser Vereinbarung ist dem Auftraggeber unverzüglich schriftlich mitzuteilen. Sofern keine Bestellung erfolgt, benennt der Auftragnehmer einen Ansprechpartner oder eine Ansprechpartnerin für den Datenschutz.
2. Die Wahrung der Vertraulichkeit, wobei der Auftragnehmer bei der Ausführung der Arbeiten ausschließlich Beschäftigte einsetzt, die auf die Vertraulichkeit verpflichtet und zuvor mit den für sie relevanten Bestimmungen zum Datenschutz vertraut gemacht wurden. Der Auftragnehmer und jede dem Auftragnehmer unterstellte Person, die Zugang zu personenbezogenen Daten hat, dürfen diese Daten ausschließlich entsprechend der Weisung des Auftraggebers verarbeiten einschließlich der in diesem Vertrag eingeräumten Befugnisse, es sei denn, dass sie gesetzlich zur Verarbeitung verpflichtet sind.
3. Die Umsetzung und Berücksichtigung aller für diese Vereinbarung notwendigen technischen und organisatorischen Maßnahmen entsprechend dem Stand der Technik.
4. Die unverzügliche Information des Auftraggebers über Kontrollhandlungen und Maßnahmen der Aufsichtsbehörde, soweit sie sich auf diese Vereinbarung beziehen. Dies gilt auch, soweit eine zuständige Behörde im Rahmen eines Ordnungswidrigkeits- oder Strafverfahrens in Bezug auf die Verarbeitung personenbezogener Daten bei der Auftragsverarbeitung beim Auftragnehmer ermittelt.
5. Durchführung der Auftragskontrolle mittels regelmäßiger Prüfungen durch den Auftragnehmer im Hinblick auf die Vertragsausführung bzw. -erfüllung, insbesondere Einhaltung und ggf. notwendige Anpassung von Regelungen und Maßnahmen zur Durchführung der AVV.
6. Auf Anfrage Auskunft über die getroffenen technischen und organisatorischen Maßnahmen gegenüber dem Auftraggeber. Hierfür kann der Auftragnehmer

auch geeignete und aktuelle Testate, Berichte oder Berichtsauszüge unabhängiger Instanzen (z.B. Wirtschaftsprüfer, Revision, Datenschutzbeauftragter, IT- Sicherheitsabteilung, Datenschutzauditoren, Qualitätsauditoren) oder eine geeignete und aktuelle Zertifizierung durch IT-Sicherheits- oder Datenschutzaudit (z.B. nach BSI-Grundschutz) vorlegen.

5. Weisungsbefugnis des Auftraggebers

1. Die Daten sind ausschließlich im Rahmen der getroffenen Vereinbarungen und nach Weisung gemäß Art 28, 29 DSGVO des Auftraggebers zu verarbeiten. Der Auftraggeber behält sich im Rahmen der in dieser Vereinbarung getroffenen Auftragsbeschreibung ein umfassendes Weisungsrecht über Art, Umfang und Verfahren der Datenverarbeitung vor, welches er durch Einzelweisungen näher bestimmen kann. Veränderungen des Verarbeitungsgegenstands und Verfahrensanpassungen sind zwischen den Parteien gemeinsam abzustimmen und zu dokumentieren. Auskünfte an Dritte oder den Betroffenen bedürfen der vorherigen schriftlichen Genehmigung seitens des Auftraggebers.
2. Weisungen des Auftraggebers erfolgen ausschließlich in Textform (schriftlich oder per E-Mail). Dem Auftragnehmer ist es untersagt, die Daten für andere Zwecke zu nutzen und er ist insbesondere nicht berechtigt, sie an Dritte weiterzugeben. Kopien und Duplikate dürfen ohne Wissen des Auftraggebers nicht erstellt werden, ausgenommen davon sind Sicherheitskopien, jedoch nur, sofern und soweit diese zur Gewährleistung einer ordnungsgemäßen Datenverarbeitung erforderlich sind, und Daten, die im Hinblick auf die Einhaltung gesetzlicher Aufbewahrungspflichten erforderlich sind.
3. Der Auftragnehmer hat den Auftraggeber unverzüglich zu informieren, wenn er der Meinung ist, eine Weisung verstoße gegen datenschutzrechtliche Vorschriften. Der Auftragnehmer ist berechtigt, die Durchführung dieser Weisung solange auszusetzen, bis sie durch den Verantwortlichen beim Auftraggeber bestätigt oder geändert wird.
4. Bei einer wesentlichen Änderung des Auftrags durch eine Weisung hinsichtlich der Datenverarbeitung steht dem Auftragnehmer ein Widerspruchsrecht zu. Besteht der Auftraggeber trotz des Widerspruchs des Auftragnehmers auf der Änderung, etwa die Umprogrammierung der Verarbeitungssoftware für Onlineumfragen, so ist diese Änderung als wichtiger Grund für den Auftragnehmer anzusehen und erlaubt eine fristlose Kündigung des von der Weisung betroffenen AVV sowie der von der AVV betroffenen Bestandteile des entsprechenden Hauptvertrages.
5. Ansprechpartner beim Auftraggeber für die Durchführung dieses Vertrages ist/sind

xy

Der Ansprechpartner ist zugleich die Person, die gegenüber dem Auftragnehmer berechtigt ist, datenschutzrechtliche Weisungen nach diesem Vertrag zu erteilen.

6. Ansprechpartner beim Auftragnehmer für die Durchführung dieses Vertrages ist:

xy

Der Ansprechpartner ist zugleich die Person, die gegenüber dem Auftraggeber berechtigt ist, datenschutzrechtliche Weisungen nach diesem Vertrag zu empfangen.

7. Die Parteien können ihre Ansprechpartner jederzeit ändern. Es können mehrere Ansprechpartner benannt werden, die jeweils einzeln weisungs- bzw. empfangsberechtigt sind. Ist der Ansprechpartner einer Partei mehr als nur vorübergehend nicht erreichbar, hat die Partei den Ansprechpartner jedenfalls

für die Dauer der Nichterreichbarkeit zu ändern. Die Änderung eines Ansprechpartners hat in dokumentierter Form zu erfolgen.

6. Überprüfungsrechte des Auftragsgebers, Kontrollrechte und Auftraggeberpflicht

Der Auftraggeber hat den Auftragnehmer unter dem Aspekt ausgewählt, dass dieser geeignete technische und organisatorische Maßnahmen aufgesetzt hat, dass die Verarbeitung im Einklang mit den Anforderungen der DSGVO erfolgt und den Schutz der Rechte der betroffenen Person gewährleistet. Der Auftraggeber ist befugt, im Vorfeld der Datenverarbeitung und sodann regelmäßig die Einhaltung der datenschutzrechtlichen Pflichten des Auftragnehmers zu kontrollieren oder durch im Einzelfall zu benennende Prüfer kontrollieren zu lassen. Die Kontrollen beziehen sich insbesondere auf die vom Auftragnehmer getroffenen technischen und organisatorischen Maßnahmen, die er gemäß den Bestimmungen dieser AVV treffen muss, um ein dem Risiko angemessenes Schutzniveau zu gewährleisten. Der Auftraggeber ist zudem befugt, durch Stichprobenkontrollen und sonstige, auch Vor-Ort-Kontrollen, die rechtzeitig anzumelden sind, die Einhaltung dieser AVV durch den Auftragnehmer in dessen Geschäftsbetrieb zu überprüfen. Der Auftragnehmer ist verpflicht, dem Auftraggeber auf Anforderung die erforderlichen Auskünfte zu geben und die entsprechenden Nachweise verfügbar zu machen.

7. Betroffenenrechte

Der Auftragnehmer ist verpflichtet, die Daten, die im Auftrag des Auftraggebers verarbeitet werden, nur nach dessen Weisung zu berichtigen, zu löschen, zu vernichten oder die Verarbeitung einzuschränken. Soweit sich ein Betroffener zur Wahrnehmung seiner Betroffenenrechte (z.B. auf Auskunft, Berichtigung oder Löschung) unmittelbar an den Auftragnehmer wenden sollte, wird der Auftragnehmer dieses Ersuchen unverzüglich an den Auftraggeber weiterleiten.

8. Unterstützungs- und Mitteilungspflichten, Datenschutz-Folgenabschätzung

Der Auftragnehmer hat den Auftraggeber bei der Erfüllung der datenschutzrechtlichen Pflichten zur Sicherheit personenbezogener Daten zu unterstützen, ebenso bei Datenschutz-Folgeabschätzungen und vorherige Konsultationen. Zudem hat er Meldepflichten bei Datenpannen. Zu seinen Pflichten im Zusammenhang gehören insbesondere:

1. die Wahrung eines angemessenen Schutzniveaus durch technische und organisatorische Maßnahmen, welche die Umstände und Zwecke der Verarbeitung sowie die prognostizierte Wahrscheinlichkeit und Schwere einer möglichen Rechtsverletzung durch Sicherheitslücken berücksichtigen und eine sofortige Feststellung von relevanten Verletzungsereignissen ermöglichen,
2. die Verpflichtung, Verletzungen insbesondere der Vertraulichkeit personenbezogener Daten unverzüglich an den Auftraggeber zu melden,
3. die Verpflichtung, den Auftraggeber im Rahmen seiner Informationspflicht gegenüber dem Betroffenen zu unterstützen und ihm in diesem Zusammenhang sämtliche relevante Informationen unverzüglich zur Verfügung zu stellen,
4. die Unterstützung des Auftraggebers bei dessen Datenschutz-Folgenabschätzung, sowie
5. die Unterstützung des Auftraggebers im Rahmen von Konsultationen der Aufsichtsbehörde. Soweit der Auftraggeber seinerseits einer Kontrolle der Aufsichtsbehörde, einem Ordnungswidrigkeits- oder Strafverfahren, dem Haftungsanspruch einer betroffenen Person oder eines Dritten oder einem anderen Anspruch im Zusammenhang mit der Auftragsverarbeitung beim Auftragnehmer ausgesetzt ist, hat der Auftragnehmer ihn nach besten Kräften zu unterstützen. Der Auftraggeber wird dem Auftragnehmer hierfür unverzüglich nach Erhalt eines Auskunftsersuchens, jedoch spätestens 14 Tage vor Ablauf der Monatsfrist gemäß Art. 12 Abs.3 DSGVO, mitteilen, wozu er konkret Auskünfte erteilen soll.

9. Verpflichtung zur Datenlöschung, Rückgabe von Datenträgern

1. Während eines laufenden Befragungsprojekts im Rahmen der Beauftragung durch den Hauptvertrag wird der Auftragnehmer die vertragsgegenständlichen Daten nur auf Anweisung des Auftraggebers berichtigen, löschen, vernichten oder deren Verarbeitung einschränken.
2. Der Auftraggeber legt die Maßnahmen zur Rückgabe der überlassenen Daten und/oder deren Löschung der gespeicherten Daten nach Beendigung einer Onlineumfrage im Rahmen der Beauftragung durch den Hauptvertrag vertraglich oder durch Weisung fest. Der Auftragnehmer berichtigt oder löscht demgemäß die vertragsgegenständlichen Daten, wenn der Auftraggeber dies anweist und dies von seinem Weisungsrahmen umfasst ist.
3. Dem Auftraggeber steht im System bei Zugängen, die ihm durch den Auftragnehmer eingerichtet worden sind, selbst die vollständige Löschmöglichkeit der Daten einer Onlineumfrage zur Verfügung, wofür ihm deswegen die eigene Datenlöschungspflicht obliegt. Sobald er seiner Löschungspflicht, die Daten in der software-as-a-service-Anwendung des Auftragnehmers zu löschen Gebrauch macht, hat der dies dem Auftragnehmer schriftlich zu bestätigen.
4. Mit Abschluss des Hauptvertrags oder früher nach Aufforderung durch den Auftraggeber – spätestens mit Beendigung der Auftragsverarbeitung – hat der Auftragnehmer dem Auftragsgeber auf Weisung alle Unterlagen in seinem Besitz, erstellte Verarbeitungs- und Nutzungsergebnisse sowie Datenbestände, die im Zusammenhang mit dem Auftragsverhältnis stehen, zu übergeben oder nach vorheriger schriftlicher Zustimmung des Auftraggebers datenschutzgerecht zu vernichten. Die Löschung bzw. Vernichtung von Datenträgern und Material mit personenbezogenen Daten hat der Auftragnehmer dem Auftraggeber mit Datumsangabe schriftlich zu bestätigen. Der Auftragnehmer ist dabei weiter verpflichtet sicherzustellen, dass Datenträger und Material mit personenbezogenen Daten entweder durch eigene Datenvernichter (Reißwolf) oder von qualifizierten Entsorgungsunternehmen vernichtet werden, welche die Vernichtung schriftlich garantieren und bestätigen. Gleiches gilt für Test- und Ausschussmaterial. Das Protokoll der Löschung ist auf Anforderung vorzulegen.
5. Dokumentationen, die dem Nachweis der auftrags- und ordnungsgemäßen Datenverarbeitung dienen, insbesondere aus Aufbewahrungsverpflichtungen aus Unionsrecht oder dem für den Auftragnehmer geltendem nationalen Recht folgen, sind durch den Auftragnehmer über die Beendigung der Vereinbarung hinaus aufzubewahren. Der entsprechende Zeitraum bestimmt sich nach den entsprechenden Aufbewahrungsfristen. Der Auftragnehmer kann sie zu seiner Entlastung bei Beendigung der Vereinbarung dem Auftraggeber übergeben. Dies gilt für die Rückgabe überlassener Datenträger und Equipment analog.
6. Der Auftragnehmer ist verpflichtet, ein Löschkonzept vorzuhalten und unmittelbar sicherzustellen, dass die Rechte auf Auskunft und auf Berichtigung sowie, soweit aufgrund datenschutzrechtlicher Bestimmungen vorgeschrieben, auf Vergessenwerden und Datenportabilität erfüllt werden können, es sei denn die Parteien haben dies ausdrücklich und schriftlich vom Leistungsumfang ausgeschlossen.
7. Entstehen nach Vertragsbeendigung oder dem Ende einer Onlineumfrage im Rahmen eines laufenden Hauptvertrags zusätzliche Kosten durch die Herausgabe oder Löschung der Daten, so trägt der Auftraggeber die hierdurch entstehenden Kosten, sofern und soweit es sich um Daten des Auftragsverhältnisses handelte, die er selbst löschen konnte. Die Parteien dieser Vereinbarung sind sich darüber einig, dass der Auftraggeber alle im Rahmen einer Onlineumfrage übermittelten oder erhobenen personenbezogenen Daten selbst in der zur Verfügung gestellten Software-as-a-Service-Lösung direkt löschen kann. Eine eventuelle Verlängerung der Aufbewahrungsdauer aufgrund von Sicherheitskopien (Backups) ist dem Löschkonzept des Auftragnehmers zu entnehmen.

10. Subunternehmer

1. Der Auftragnehmer nimmt zurzeit folgende weitere Auftragsverarbeiter als Subauftragnehmer in Anspruch:
    - PartnerGate GmbH (VPS-Hosting Webserver)
      Wilhelm-Wagenfeld-Str. 16
      80807 München
    - LOX24 GmbH (Versand von SMS)
      Seestraße 109
      13353 Berlin
2. Zum Zeitpunkt des Abschlusses dieser Vereinbarung sind die vorstehenden aufgeführten Unternehmen als Unterauftragnehmer für Teilleistungen für den Auftragnehmer tätig und verarbeiten und/oder nutzen in diesem Zusammenhang auch unmittelbar die Daten des Auftraggebers. Für diese Unterauftragnehmer gilt die Einwilligung für das Tätigwerden als erteilt. Eine Datenübermittlung in ein Drittland findet hierdurch nicht statt.
3. Der Auftragnehmer ist berechtigt, weitere Unterauftragnehmer hinzuzuziehen oder die in Anspruch genommenen Unterauftragnehmer durch andere Unterauftragnehmer zu ersetzen. Der Auftragnehmer informiert den Auftraggeber jedoch vorab über die beabsichtigte Änderung in Bezug auf die Hinzuziehung oder Ersetzung und darüber, ob hierdurch eine übermittlung in ein Drittland stattfindet. Der Auftraggeber kann gegen die beabsichtigte Änderung Widerspruch erheben. Der Widerspruch ist innerhalb einer Ausschlussfrist von sechs Wochen ab Erhalt der Information über die beabsichtigte Änderung zu erheben. Sowohl die Information als auch der Widerspruch bedürfen der Textform, wobei der Auftragnehmer den Auftraggeber in der Information noch einmal auf die Ausschlussfrist hinweisen wird. Erhebt der Auftraggeber ohne wichtigen Grund Widerspruch gegen die Änderung, ist der Auftragnehmer mit einer Frist von sechs Wochen zur vorzeitigen Kündigung sowohl dieses Vertrages als auch des Hauptvertrages berechtigt.
4. Die Beauftragung von Auftragnehmern/Subunternehmern außerhalb der EU/des EWR wird ausgeschlossen.
5. Der Auftragnehmer wird den Unterauftragnehmern im Wege eines Vertrages dieselben Datenschutzpflichten auferlegen, die in diesem Vertrag zwischen den Parteien festgelegt sind.
6. Dienstleistungen, die der Auftragnehmer bei Dritten als Nebenleistung zur Unterstützung bei der Durchführung der AVV in Anspruch nimmt, stellen keine Subunternehmerverhältnisse im Sinne dieser Regelung dar. Dazu zählen z.B. Telekommunikationsleistungen, Wartung und Benutzerservice, Reinigungskräfte oder Prüfer. Der Auftragnehmer ist jedoch verpflichtet, zur Gewährleistung des Schutzes und der Sicherheit der Daten den Auftraggeber auch bei fremd vergebenen Nebenleistungen angemessene und gesetzeskonforme vertragliche Vereinbarungen zu treffen sowie Kontrollmaßnahmen zu ergreifen.

11. Besondere Vorschriften bei Fernwartung

Folgende Bestimmungen und ergänzende Vorgaben finden Anwendung im Falle eines Fernwartungszugriffs durch den Auftragnehmer, sofern und soweit dies für die Vertragserfüllung des Hauptvertrags oder diese Vereinbarung erforderlich ist oder erforderlich werden kann.

1. Fernwartungsarbeiten dürfen nur mit Genehmigung des Auftraggebers erfolgen. Fernwartung erfolgt dergestalt, dass der Auftraggeber dem Auftragnehmer für ein Befragungsprojekt im Rahmen der Software des Auftragnehmers Verwaltungszugriff einräumt. Ein Fernwartungszugriff des Auftragnehmers auf Datenverarbeitungsanlagen des Auftraggebers selbst erfolgt hierbei nicht.
2. Die Fernwartung ist mindestens durch die gleichen Sicherheitsmaßnahmen (Benutzername und Passwort, verschlüsselte Datenübertragung) geschützt wie der Zugriff des Auftraggebers auf das Befragungsprojekt.
3. Dem Auftragnehmer werden durch den Auftraggeber Zugriffsrechte eingeräumt, die dieser zur Durchführung der Fernwartungsarbeiten tatsächlich benötigt. Der Auftraggeber stellt sicher, dass der Auftragnehmer nur insoweit auf gespeicherte

personenbezogene Daten zugreifen kann, als dies zur Durchführung der Fernwartungsarbeiten unerlässlich notwendig ist.

4. Der Auftragnehmer darf von den ihm eingeräumten Zugriffsrechten nur insoweit für die Durchführung der Fernwartungsarbeiten unerlässlich notwendigen Gebrauch machen.
5. Der Auftraggeber ist berechtigt, die Fernwartungsarbeiten von einem Kontrollbildschirm aus zu verfolgen und jederzeit abzubrechen. Soweit der Auftragnehmer daran mitwirken muss, gewährleistet er, dass dies möglich ist.

12. Haftung

1. Auftraggeber und Auftragnehmer haften für den Schaden, der durch eine nicht den Datenschutzgesetzen entsprechende Verarbeitung verursacht wird, gemeinsam im Außenverhältnis gegenüber dem jeweils Betroffenen. Der Auftragnehmer haftet dabei ausschließlich für Schäden, die auf einer von ihm durchgeführten Verarbeitung beruhen, bei der
    - er den aus der DSGVO resultierenden und speziell für Auftragsverarbeiter auferlegten Pflichten nicht nachgekommen ist oder
    - er unter Nichtbeachtung der rechtmäßig erteilten Anweisungen des Auftraggebers handelte oder
    - er gegen die rechtmäßig erteilten Anweisungen des Auftraggebers gehandelt hat.
2. Kommt ein weiterer Auftragsverarbeiter (Subunternehmer) seinen Datenschutzpflichten nicht nach, so haftet der erste Auftragsverarbeiter gegenüber dem Verantwortlichen für die Einhaltung der Pflichten jenes anderen Auftragsverarbeiters (Subunternehmers).
3. Soweit der Auftraggeber zum Schadensersatz gegenüber dem Betroffenen verpflichtet ist, bleibt ihm der Rückgriff auf den Auftragnehmer vorbehalten. Im Innenverhältnis zwischen Auftraggeber und Auftragnehmer haftet der Auftragnehmer für den durch eine Verarbeitung verursachten Schaden jedoch nur, wenn er
    - seinen ihm speziell durch die DSGVO auferlegten Pflichten nicht nachgekommen ist oder
    - unter Nichtbeachtung der rechtmäßig erteilten Anweisungen des Auftraggebers oder gegen diese Anweisungen gehandelt hat.
4. Weitergehende Haftungsansprüche nach den allgemeinen Gesetzen bleiben unberührt.

13. Kosten

1. Der Auftragnehmer erbringt die Umsetzung der durch den Hauptvertrag festgelegten Weisungen und sorgt für die Einhaltung der allgemeinen und technischen und organisatorischen Maßnahmen, ohne dem Auftraggeber dafür Kosten nach diesem Vertrag zu berechnen. Insoweit sind die Tätigkeiten des Auftragnehmers also schon durch die Vergütung nach Maßgabe des Hauptvertrages abgegolten. Das gleiche gilt für Einzelweisungen, die der Auftraggeber über das Verarbeitungssystem des Auftragnehmers nach dem Hauptvertrag selbst umsetzen kann und auch selbst umsetzt (bspw. eigene Löschungspflicht von Daten gemäß Ziffer 9.3).
2. Dagegen fallen Kosten für die Umsetzung von Einzelweisungen und sonstiger Verlangen, welche über den Regelbetrieb hinausgehen beziehungsweise nicht Gegenstand des Hauptvertrags sind, dem Auftraggeber zur Last. Dies gilt insbesondere für die Unterstützung bei der Beantwortung von Betroffenenanträgen und bei der Einhaltung sonstiger Pflichten, die dem Auftraggeber obliegen, für die Rückgabe und Vernichtung von Daten entsprechend Ziffer 9.7, soweit diese über eine Löschung im System des Auftragnehmers hinausgeht, für die Zurverfügungstellung von Informationen, soweit diese nicht überwiegend im Interesse des Auftragnehmers liegt, und für das Ermöglichen und Beitragen zu Prüfungen einschließlich Inspektionen, soweit diese über eine verhältnismäßige Prüfung beim Auftragnehmer hinausgehen.

3. Auf Verlangen wird der Auftragnehmer dem Auftraggeber vorab eine Kostenschätzung geben. Zu den Kosten gehört auch eine angemessene Vergütung des Arbeitsaufwands. Der Stundensatz beträgt **120 € zzgl. USt.** Abweichende Kostenregelungen aus dem Hauptvertrag oder einer in den Hauptvertrag einbezogenen Preisliste, die sich auf datenschutzrechtliche Maßnahmen beziehen, gehen dieser Kostenregelung vor. Ebenso fallen die Kosten für Maßnahmen, deren Erforderlichkeit eine Partei schuldhaft verursacht hat, dieser Partei zur Last. Ein Mitverschulden der jeweils anderen Partei ist jedoch zu berücksichtigen.

14. Vertragsbeendigung, Schlussbestimmungen

1. Unbeschadet sonstiger Bestimmungen des Vertrags, insbesondere Ziffer 2.4, ist der Auftraggeber berechtigt, den Hauptleistungsvertrag und diese AVV jederzeit ohne Einhaltung einer Frist zu kündigen, wenn der Auftragnehmer schwerwiegend gegen eine Bestimmung dieser AVV verstößt, eine datenschutzrechtliche Weisung gemäß Ziffer 5 dieser AVV nicht umsetzt oder Kontrollen des Auftraggebers gemäß vorstehender Ziffer 6 dieser AVV verweigert.
2. Weisungen des Auftraggebers, die als wesentliche Vertragsänderungen durch den Auftraggeber zu verstehen sind, insbesondere aber nicht abschließend bei einer Weisung entsprechend der Regelung in Ziffer 5.4, ist der Auftragnehmer seinerseits zur außerordentlichen Kündigung dieser AVV wie des zugrundeliegenden Hauptvertrags berechtigt.
3. Sollten die Daten des Auftraggebers bei dem Auftragnehmer durch Pfändung oder Beschlagnahme, durch ein Insolvenz- oder vergleichbare Verfahren oder durch sonstige Ereignisse oder Maßnahmen Dritter gefährdet werden, so hat der Auftragnehmer den Auftraggeber unverzüglich darüber zu informieren. Der Auftragnehmer wird alle in diesem Zusammenhang Verantwortlichen unverzüglich darüber informieren, dass die Hoheit und das Eigentum an den Daten ausschließlich beim Auftraggeber als Verantwortlichem im Sinne der Datenschutzgrundverordnung liegen.
4. Änderungen, Ergänzungen und die Aufhebung dieses Vertrages müssen in dokumentierter Form erfolgen. Dies gilt entsprechend für die Änderung dieser Formklausel. Dokumentierte Form im Sinne dieses Vertrages meint mindestens die Textform. Auf Verlangen einer Partei ist eine in Textform abgegebene Erklärung schriftlich zu bestätigen.

Für den Auftragnehmer

Für den Auftraggeber

Zur Online-Vereinbarung vorgemerkt
